# Topical formulation of repurposed FDA-approved compounds inhibits *Pseudomonas aeruginosa* ExoU and improves corneal infection outcomes

**DOI:** 10.1371/journal.ppat.1014474

**Published:** 2026-08-03

**Authors:** Daniel M. Foulkes, David G. Fernig, Keri McLean, Marie Held, John A. Harris, Yan Sun, Joanne L. Fothergill, Valerie Price, Dominic P. Byrne, Gabriela Czanner, Connie Tam, Stephen B. Kaye

**Affiliations:** 1 Department of Eye and Vision Sciences, Institute of Life Course and Medical Sciences (ILCAMS), University of Liverpool, Liverpool, United Kingdom; 2 Department of Biochemistry, Cell and Systems biology, Institute of Systems, Molecular and Integrative Biology, University of Liverpool, Liverpool, United Kingdom; 3 Centre for Cell Imaging, Technology, Infrastructure and Environment Directorate, Faculty of Health and Life Sciences, University of Liverpool, Liverpool, United Kingdom; 4 Cole Eye Institute, Cleveland Clinic, Cleveland, Ohio, United States of America; 5 Department of Ophthalmic Research, Cleveland Clinic Research, Cleveland, Ohio, United States of America; 6 Department of Clinical Infection, Microbiology and Immunology, Institute of Infection, Veterinary & Ecological Sciences, University of Liverpool, Liverpool, United Kingdom; 7 Faculty of Engineering and Physical Sciences, Faculty of Medicine, University of Southampton, Southampton, United Kingdom; 8 Department of Ophthalmology, Cleveland Clinic Lerner College of Medicine, Case Western Reserve University, Cleveland, Ohio, United States of America; Université de Reims Champagne-Ardenne: Universite de Reims Champagne-Ardenne, FRANCE

## Abstract

Microbial keratitis caused by Exotoxin U (ExoU)-producing strains of *Pseudomonas aeruginosa* is associated with poor clinical outcomes and reduced responsiveness to antimicrobial therapy. ExoU, a phospholipase, is secreted directly into host cells, causing their lysis. A screen of 3,034 FDA-approved compounds identified zinc pyrithione (Zp), bismuth subcitrate (Bis), and polymyxin B (Pol) as lead inhibitors of ExoU selectively inhibiting it without affecting human PLA2s or bacterial viability. The compounds have distinct inhibitory mechanisms: Zp disrupted ExoU oligomerization and protein stability; Bis impaired phosphatidylinositol 4,5-bisphosphate-dependent membrane association; Pol directly inhibited catalysis via its lipid-peptide architecture. Compound combinations enhanced ExoU inhibition *in vitro* to nanomolar concentrations. In mammalian cells, Bis and Pol promoted lysosomal trafficking and degradation of ExoU. A high content screen of 52 *P. aeruginosa* keratitis isolates demonstrated the broad efficacy of these inhibitors in *exoU*⁺ strains. Therapeutic efficacy was evaluated in *ex vivo* porcine corneas, *Galleria mellonella*, and *in vivo* mouse keratitis models. Topical delivery of ExoU inhibitors, particularly in combination, significantly reduced corneal opacity, ulceration, and stromal damage in porcine corneas without affecting bacterial load. In *Galleria*, compound combinations significantly maintained larval health, improved larval survival and delayed mortality. In a mouse eye infection model, combinatorial treatment reduced disease severity and preserved tissue viability without altering bacterial burden. These findings validate ExoU as a druggable virulence factor and support the repurposing of these compounds as an antivirulence strategy for the treatment of *P. aeruginosa* infections in human beings and in veterinary medicine.

## Author summary

*Pseudomonas aeruginosa* is a leading cause of microbial keratitis, a sight-threatening infection characterised by rapid tissue destruction. A major contributor to disease severity is the type III secretion system effector ExoU, a potent phospholipase that induces acute host cell lysis. Current treatment strategies rely on antimicrobials to control bacterial growth but do not directly address toxin-mediated damage. Here, we investigated an antivirulence approach aimed at inhibiting ExoU activity. Through screening of clinically approved compounds, we identified zinc pyrithione, bismuth subcitrate and polymyxin B as inhibitors of ExoU; each acting via distinct but complementary mechanisms. Across cellular, *ex vivo* porcine corneal, and *in vivo* infection models, these compounds, particularly in combination, significantly reduced ExoU-mediated cytotoxicity and preserved tissue integrity. Notably, these protective effects occurred without reductions in bacterial burden, consistent with a mechanism targeting virulence rather than viability. Our findings support the feasibility of repurposing approved drugs to neutralise bacterial toxins and highlight ExoU inhibition as a promising adjunctive strategy to limit tissue damage and improve outcomes in microbial keratitis.

## Introduction

The opportunistic pathogen *Pseudomonas aeruginosa* is the primary causative agent of bacterial keratitis and is a leading cause of clinical blindness, particularly in contact lens wearers and immunocompromised individuals [1]. It is also a leading cause of intensive care unit-acquired pneumonia (ICUAP) [2], and is the second most frequent colonising bacteria in patients with COVID19 [2,3]. Reflecting its substantial clinical burden and increasing antimicrobial resistance, the World Health Organization (WHO) 2024 Bacterial Priority Pathogens List designates carbapenem-resistant *Pseudomonas aeruginosa* (CRPA) as a high-priority pathogen for research and development of new antibacterial treatments [4].

The type III secretion system (T3SS) is a major virulence determinant in *P. aeruginosa* infections and is strongly associated with poor clinical outcomes, including in pneumonia and microbial keratitis [5,6]. Among the effectors secreted via this system, ExoU and ExoS are the principal toxins, and their presence is almost always mutually exclusive: strains encoding *exoU* typically lack *exoS* and *vice versa* [7]. ExoU, a patatin-like phospholipase A2, is considered the most cytotoxic T3SS effector in the eye and is strongly linked to rapid epithelial injury, tissue necrosis, and severe outcomes such as corneal ulceration and vision loss [8,9]. In contrast, ExoS is a bifunctional toxin with GTPase-activating and ADP-ribosyltransferase activities, which disrupts host cell signalling and deregulates actin dynamics in more chronic infections [8].

In microbial keratitis, *exoU*^+^ strains predominate, being detected in 61.5% of clinical isolates, and are consistently associated with worse clinical outcomes, increased antimicrobial resistance and heightened inflammation [10,11]. Thus, ExoU causes significant cell lysis, while other virulence factors may additionally contribute to the pathology. Mechanistically, after secretion by the T3SS into the host cell ExoU requires binding to ubiquitin and phosphatidylinositol 4,5-bisphosphate (PIP_2_) for activation [12,13]. PIP_2_ binding promotes ExoU oligomerisation, markedly enhancing its ubiquitin-dependent catalytic activity [13,14]. In mammalian cells, ExoU localises to the plasma membrane via its C-terminal four-helical bundle domain, where its phospholipase activity rapidly lyses host cells [15,16]. This enzymatic activity also liberates arachidonic acid, triggering NF-κB and mitogen-activated protein kinase (MAPK) signalling cascades [17–19]. The subsequent upregulation of proinflammatory mediators, including IL-8 and keratinocyte chemoattractant (KC), drives neutrophil recruitment and intense local inflammation, exacerbating tissue destruction [17,18].

Since antimicrobial agents fail to address ExoU-mediated host cell destruction, antivirulence agents are a promising therapeutic strategy. Indeed, several therapies are currently in clinical development [20], including the monoclonal antibody MEDI3902 which targets PcrV of the *P. aeruginosa* T3SS [21]. More broadly, a range of small-molecule inhibitors targeting the T3SS have been described, including compounds that disrupt secretion apparatus assembly, effector translocation, or regulatory pathways controlling T3SS expression. For example, phenoxyacetamide derivatives have been shown to inhibit T3SS function through structure-dependent interactions with secretion components, while other screening approaches have identified inhibitors that attenuate virulence without directly affecting bacterial viability [22,23]. These strategies highlight the therapeutic potential of targeting virulence mechanisms rather than bacterial survival, an approach that may reduce selective pressure for resistance, make pathogens more vulnerable to clearance by the immune system or render them more susceptible to traditional antibiotics [24].

Most T3SS-targeting strategies act upstream of effector delivery and do not directly inhibit toxin activity once translocated into host cells. In contrast, targeting ExoU directly within the host cell offers a precision antivirulence approach, with the potential to limit ongoing tissue damage even after infection has been established, while preserving host tissue integrity and avoiding disruption of the microbiota [8,25].

Repurposing FDA-approved drugs offers a pragmatic strategy for ExoU inhibition, which has the potential to bypass the lengthy and costly development timelines associated with developing novel small molecules. Here, we employed a high-throughput phospholipase activity screen to identify inhibitors of ExoU from a library of over 3,000 clinically approved compounds. Our screen identified three compounds, zinc pyrithione (Zp), bismuth subcitrate (Bis), and polymyxin B (Pol), that inhibit ExoU via distinct mechanisms, including direct catalytic blockade, conformational destabilisation, and toxin mislocalisation within host cells. Through a series of *in vitro*, cellular, *ex vivo*, and *in vivo* assays, we demonstrate that these agents, especially in combination, significantly reduce ExoU-driven cytotoxicity and preserve epithelial integrity in corneal infection models.

These findings establish the feasibility of targeting ExoU directly and lay the foundation for future therapeutic development aimed at mitigating *P. aeruginosa* virulence in ocular and other mucosal infections.

## Results

Previously we established a manual pipeline for screening inhibitors of ExoU phospholipase activity and determining their ability to protect human corneal cells, HCE-T in infection and cytotoxicity assays [25]. To identify ExoU inhibitors with translational potential, we employed our high throughput phospholipase assay to screen 3,034 compounds in the SelleckChem FDA approved library. The efficacy of compounds in host HCE-T cells was automated in high-content fluorescence microscopy assays to enable a broader screen of the *in vitro* active inhibitors against clinical strains of *P. aeruginosa*. Mechanistic information on the lead inhibitors was acquired by analysis of their pharmacophores and the effects on ExoU structure and localisation in transfected HEK293T cells. Finally, the efficacy of the lead inhibitors was tested in three more relevant models, *ex vivo* pig corneas, *G. mellonella* larvae and a mouse model of microbial keratitis.

### Repurposing FDA-approved drugs: a high-throughput screen for ExoU phospholipase inhibitors

Recombinant ExoU bearing a C-terminal 6 × His tag was expressed in *E. coli* and purified using immobilised metal affinity chromatography (IMAC) followed by size-exclusion chromatography, as described previously [25] (S1 Fig). ExoU *in vitro* phospholipase activity was analysed using a real-time, high-throughput phospholipase assay based on a modified version of the Cayman Chemical PLA_2_ kit, adapted for 96- and 384-well formats [25] (S1 Fig). The assay was conducted in the presence of PIP_2_ and ubiquitin, cofactors essential for ExoU activation and catalysis.

**Fig 1:**
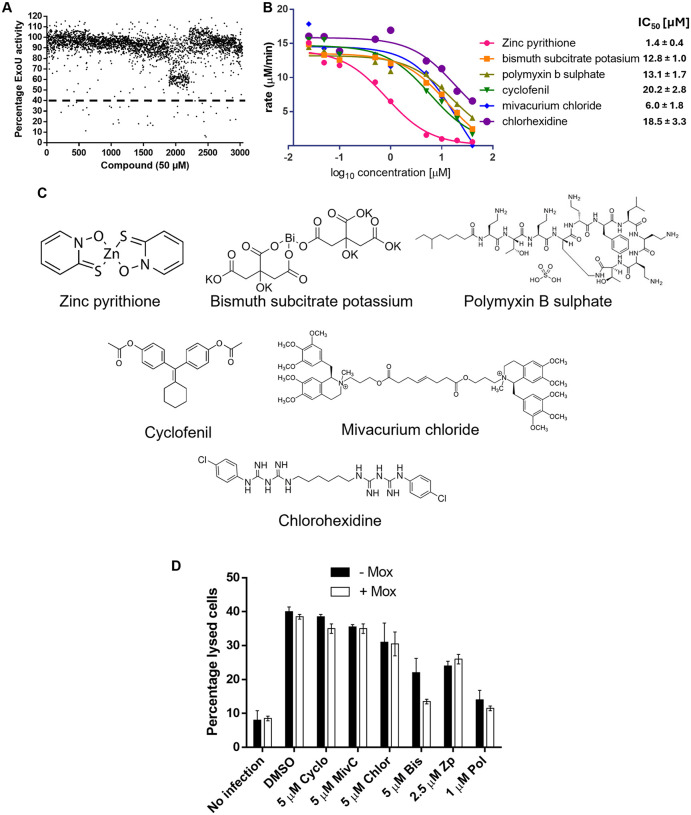
High-throughput screening identifies FDA-approved ExoU inhibitors with activity in vitro and in cell-based infection models. **(A)** A high-throughput *in vitro* phospholipase assay with 175 nM ExoU (specific activity 5.85 ± 0.26 μmol/min/μg) and absorbance read at 414 nm was used to screen 3,042 compounds (50 µM) from the Selleckchem FDA-approved drug library to identify novel inhibitors of ExoU. The plot shows percentage inhibition of ExoU phospholipase activity after a 1 h enzymatic reaction, with reference to 0.1% (v/v) DMSO controls (100% activity). **(B)** Dose-response curves of selected compounds were generated to determine IC₅₀ values for ExoU inhibition *in vitro*. **(C)** Chemical structures of lead compounds. **(D)** High-content imaging screen of lead ExoU inhibitors using a high-throughput scratch and infection assay. Human corneal epithelial (HCE-T) cells were infected with *P. aeruginosa* PA103 (MOI 10). After 4 h, cell lysis was quantified by ethidium homodimer staining. The bar graph shows the percentage of lysed cells, reflecting ExoU-mediated cytotoxicity, following treatment with cyclofenil (Cyclo), mivacurium chloride (MivC), chlorhexidine (Chlor), bismuth subcitrate (Bis), zinc pyrithione (ZP), and polymyxin B (Pol). White bars indicate compounds alone, while black bars indicate compounds in the presence of 2.5 µM moxifloxacin (mox). No infection indicates vehicle controls of 0.1% (v/v) DMSO control without the addition of PA103.

In the initial screen of 3,042 FDA-approved compounds from the Selleckchem library (Fig 1A), those that reduced ExoU activity to 40% or less, relative to DMSO-treated controls, were advanced to dose-response analysis. These were performed to eliminate false positives and determine IC₅₀ values of confirmed inhibitors (Fig 1B), with compound structures illustrated in Fig 1C.

Of the 35 initial screening hits, we first excluded any compounds that either absorbed at ~414 nm or otherwise interfered with the DTNB secondary reporter reaction or were unsuitable for therapeutic development (e.g., chemotherapy agents). Six showed clear inhibitory activity in the low micromolar range. Zp emerged as the most potent inhibitor identified to date, with an IC₅₀ of 1.4 ± 0.4 μM (Fig 1B) and a Kᵢ of 2.4 μM (S2 Fig). Several additional compounds showed strong to moderate inhibition, including mivacurium chloride (MivC; IC₅₀ = 6.0 ± 1.8 μM), bismuth subcitrate (Bis; 12.8 ± 1.0 μM), polymyxin B (Pol; 13.1 ± 1.7 μM), chlorhexidine (Chlor18.5 ± 3.3 μM), and cyclofenil (20.2 ± 2.8 μM).

With the exception of Cyclofenil, a selective estrogen receptor modulator with reported adverse effects [26] that could make it unsuitable for ocular application, the other hits represent attractive candidates for repurposing as treatments for *P. aeruginosa* keratitis. These included polymyxin B, a cationic lipopeptide antimicrobial, with dose-limiting neurotoxicity when administered systemically, and chlorhexidine, a broad-spectrum antiseptic used in ophthalmic and periocular disinfection. Notably, several compounds, including polymyxin B and chlorhexidine are already used topically in ophthalmology [1,27], enhancing their translational potential. Zp, although not currently used for eye infections has been used as an active ingredient in anti-dandruff shampoos. Its applications span across medical treatments (anti-fungal), household products, and industrial materials [28]. Zp exhibited promising potency against ExoU, prompting us to investigate it further. The full data from the initial screen comprising initial ‘hits’, compounds that interfered with assay readouts and dose response validated compounds are in S1 Table.

### Determining efficacy of lead ExoU inhibitors in a cellular wound infection model

ExoU is synthesised in *P.* aeruginosa as an inactive complex with the SpcU chaperone [29]. Dissociation from SpcU and significant unfolding are required for its translocation through the T3SS into host cells, where is it refolded and then exerts its activity. Thus, it was important to evaluate the efficacy of ExoU inhibitors in a cellular context. A high-throughput epithelial infection model was developed based on our previously reported scratch wound assay in HCE-T corneal epithelial cells [25]. The assay was adapted to a 96-well plate format compatible with high-content live-cell imaging using the IncuCyte S3 system. Uniform scratch wounds were generated using the IncuCyte WoundMaker, and HCE-T cells were infected with ExoU-producing *P. aeruginosa* PA103 (MOI 10). Cell viability and lysis were continuously monitored for 15 h at 30 min intervals using calcein-AM (viable cell marker) and ethidium homodimer-1 (membrane damage/lysis marker) (Fig 1D).

Because *P. aeruginosa* replicates rapidly *in vitro*, as before [25], we incorporated sub-MIC moxifloxacin (2.5 μM; 50% MIC) to slow bacterial growth, extend the infection window and enhance detection of ExoU-dependent cytotoxicity. Moxifloxacin does not inhibit ExoU nor the T3SS [30], ensuring that observed effects are not due to interference with toxin delivery or activity.

DMSO-treated, uninfected monolayers exhibited minimal background cell death (8.0 ± 1.8% ethidium-positive cells at 10 h). Sub-MIC Moxifloxacin was confirmed to be non-toxic to HCE-T cells, as ethidium homodimer uptake remained unchanged compared to untreated controls (<1% difference at 10 h). In contrast, infection with PA103 in DMSO-treated wells induced robust ExoU-dependent lysis, with ~40% of cells staining positive for ethidium homodimer at 10 h (Fig 1D). The inclusion of sub-MIC moxifloxacin did not appreciably reduce cytotoxicity (38.8% lysis), confirming, in line with our previous studies [30], that ExoU-mediated epithelial injury occurs even under conditions of partially restricted bacterial growth (Figs 1D and S3 Fig).

We next evaluated the six lead compounds in the infection model (Fig 1D). Cyclofenil, MivC, and chlorhexidine did not reduce ExoU-mediated lysis, whereas Bis, Zp, and Pol conferred clear protection of HCE-T cells.

To determine whether this protection reflected antibacterial activity, we assessed bacterial growth across a range of concentrations (S3 Fig). While Zp, chlorhexidine, and Pol exhibited dose-dependent bactericidal activity at higher concentrations, the concentrations used in the infection assays were below these thresholds and did not measurably affect bacterial viability. This was confirmed by both growth curves and endpoint CFU enumeration following IncuCyte imaging (S3 Fig), demonstrating that compounds combined with 2.5 µM moxifloxacin did not increase bactericidal activity (S3 Fig).

Polymyxin B exhibited an IC₅₀ of 0.75 µM in LB and 2.1 µM in DMEM/F-12 supplemented with 10% (v/v) FBS, likely reflecting serum interactions. The concentration used in the cell-based infection assays (1 µM) did not significantly reduce bacterial viability under these conditions (S3 Fig).

Collectively, these findings identified Pol, Bis, and Zp as ExoU targeting inhibitors capable of protecting corneal epithelial cells from *P. aeruginosa* cytotoxicity independent of bacterial killing. This also establishes the infection model as a robust platform for future screening of antivirulence therapeutics.

### Selectivity of the ExoU inhibitors

To establish the selectivity of the ExoU inhibitors, they were also screened against PLA2G7 and PLA2G4C which represent two distinct and major classes of human calcium-independent PLA_2_ [31,32], to evaluate the selectivity of our hits for bacterial ExoU over host phospholipases. Using 2-thio-PAF as the substrate for human PLA2G7 we found that neither Bis, Pol, nor Zp inhibited enzymatic activity, whereas darapladib, a clinical PLA2G7 inhibitor, effectively suppressed it (S4 Fig). In contrast, darapladib did not inhibit ExoU activity when arachidonoyl thio-PC was used as the substrate (S4 Fig). Similarly, using arachidonoyl thio-phosphatidylcholine (thio-BGPC) as a substrate for PLA2G4C we observed no inhibition by Bis, Pol or Zp (S4 Fig). The use of different thio-ester substrates also underscored this selectivity: while darapladib effectively blocked PLA2G7 with its preferred substrate, it had no effect on ExoU. The absence of inhibition by Bis, Pol and Zp of these enzymes suggests that they selectively targeted ExoU without inhibiting human PLA_2_ activity, reducing the likelihood of host toxicity.

The cytotoxicity of the compounds was measured directly in primary human corneal epithelial cells (S5 Fig). The LDH assays showed that Bis had no measurable toxicity up to 160 µM, while Zp and Pol had LD₅₀ of 32.6 ± 12.6 µM and 42.5 ± 10.6 µM, respectively. Importantly, these LD₅₀ values were an order of magnitude higher than the concentrations applied in the infection assays.

### ExoU is inhibited by zinc and bismuth ions

**Fig 2:**
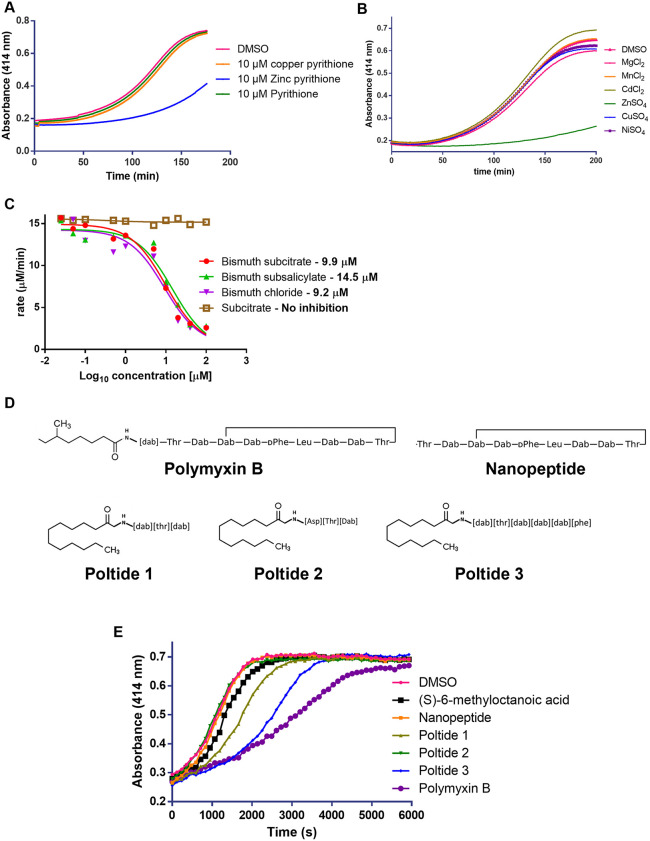
Chemical features associated with ExoU inhibition: metal ions and fatty acid peptides. **(A)** Real-time phospholipase assay showing ExoU inhibition in the presence of pyrithione and pyrithione-coordinated zinc or copper complexes. **(B)** ExoU phospholipase activity detected in the presence of a panel of selected metal salts. **(C)** Dose-response phospholipase assays used to determine IC₅₀ values for bismuth-based ExoU inhibitors and subcitrate alone. **(D)** Chemical structures of native polymyxin B, the cyclic peptide fragment of polymyxin B (nanopeptide), and custom-synthesized polymyxin-derived fatty acid peptides: Poltide 1 (truncated polymyxin mimic), Poltide 2 (negatively charged sequence), and Poltide 3 (linear polymyxin-like analog). **(E)** Real-time phospholipase activity assay demonstrating ExoU inhibitory profiles of each polymyxin compound.

To determine the ExoU inhibitory active pharmacophore in Zp, we assessed copper pyrithione, pyrithione alone and Zn^2+^ *in vitro* phospholipase assays (Fig 2A). Neither copper pyrithione, nor pyrithione inhibited ExoU (Fig 2A), suggesting that ExoU activity was sensitive to zinc. To further probe this, a panel of metal salts was tested and only zinc sulphate inhibited ExoU phospholipase activity (Fig 2B). We therefore, then determined whether Zn^2+^ alone could inhibit ExoU-mediated cytotoxicity (S6 Fig). LDH release assays in PA103-infected HCE-T cells showed that ZnSO_4_ (2.5 µM) did not reduce cell lysis compared to the DMSO control (mean lysis: 89% vs. 85%), whereas Zp (2.5 µM), comprising a pyrithione zinc ionophore, significantly reduced cytotoxicity (mean lysis: 55%). These findings indicate that zinc ions required a carrier to enter host cells and inhibit ExoU activity and that the Zn^2+^chelate Zp is itself active.

The ExoU inhibitory active pharmacophore in Bis was determined using other bismuth containing compounds, bismuth subsalicylate, bismuth chloride and subcitrate in phospholipase assays. All bismuth containing compounds inhibited ExoU activity, whereas subcitrate alone did not (Fig 2C). Thus, the Bi^3+^ ion was the pharmacophore in the chelate and the chelator subcitrate likely a carrier.

### Inhibition by Polymyxin B depends on its peptide and acyl tail combined

Pol comprises a cyclic cationic decapeptide linked to a hydrophobic fatty acyl tail (Fig 2D) and the inhibitory activity of these structural elements was assayed individually. The isolated hydrophobic tail ((S)-6-methyloctanoic acid) failed to inhibit ExoU (Fig 2E). The cyclic peptide portion of polymyxin B alone (nanopeptide) also did not inhibit ExoU. This indicated that Pol’s inhibitory activity likely arose from synergy between its cationic peptide and lipid tail.

The role of the fatty acyl-peptide linkage in ExoU inhibition, was examined by using three custom “poltide” analogues (Fig 2D), each bearing an N-terminal lauric acid (C₁_2_) lipid attached to a simplified polymyxin-inspired peptide. Poltide 1 contained an N-terminal lauric acid followed by –Dab–Thr–Dab. In Poltide 2, the charge of the first 2,4-diaminobutyric acid residue was reversed by replacing it with an aspartic acid (poltide 2: lauric acid–Asp–Thr–Dab), while Poltide 3 was extended by consisting of lauric acid followed by Dab–Thr–Dab–Dab–Dab–Phe. Poltide 1 inhibited ExoU, albeit more weakly than Pol (IC₅₀ 40 µM), whereas Poltide 2, bearing an opposite charge in the first amino acid residue, had no effect on ExoU activity (Fig 2E). Poltide 3 was more potent than Poltide 1 (IC₅₀ 20 µM) but did not recapitulate fully the full inhibitory activity of Pol (IC₅₀ 13 µM). These data highlight that both peptide length and charge, and potentially its cyclic structure are critical for ExoU inhibition.

### *In vitro* biochemical effects of ExoU inhibition by Bis, Pol and Zp

The effects of Bis, Pol and Zp on the thermal stability of ExoU, a proxy for its structure/conformation, and oligomeric assembly were measured using nanoDSF and BS^3^ crosslinking.

### Thermal stability of ExoU

ExoU had a melting temperature (Tₘ) of 40.8 °C (S7 Fig). Addition of 5 µM PIP_2_ increased the Tₘ to 43.1 °C, indicating that PIP_2_ binding alters the apparent thermal stability of ExoU under assay conditions. Zp decreased the Tₘ of ExoU by 1.8 °C in the absence of PIP_2_ and by 1.3 °C in its presence, suggesting a modest destabilising effect that is largely independent of lipid binding. Pol produced a more pronounced decrease in thermal stability, lowering the Tₘ by 3.8 °C without PIP_2_ and by 6.2 °C in its presence. Bis had no measurable effect on Tₘ in the absence of lipid, but in the presence of PIP_2_ reduced the Tₘ by ~2 °C. Collectively, these data indicate that all three compounds interact with ExoU and modulate its thermal stability in a PIP_2_-dependent (Bis) or PIP_2_-independent (Zp and Pol) manner.

**Fig 3:**
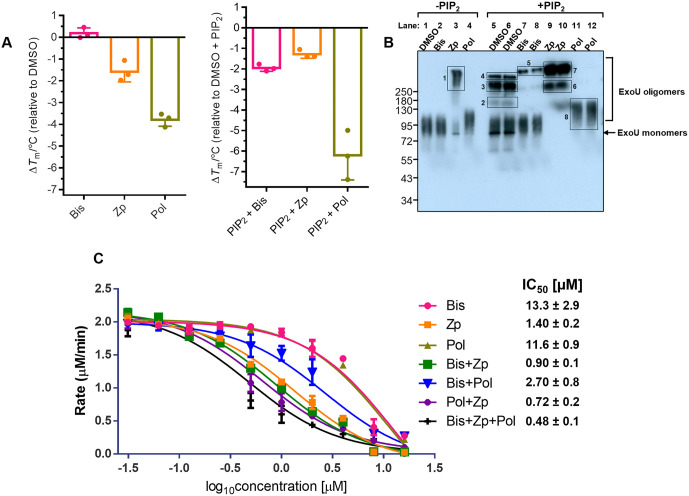
ExoU destabilisation, oligomerisation and inhibition in the presence of inhibitors and PIP_2_. **(A)** Nano differential scanning fluorimetry (nanoDSF) analysis of ExoU thermal stability in the presence of compounds (10 µM) without (left) and with (right) PIP_2_ (5 µM) present. Chart summarises ΔTm values with standard deviation. **(B)** BS^3^ cross-linking assay of recombinant ExoU followed by SDS-PAGE and anti-6 × His immunoblotting, showing compound-dependent changes in oligomerisation in the presence or absence of PIP_2_. ExoU was incubated with 10 µM inhibitor (Bis, Zp, or Pol) or DMSO control ± PIP_2_ for 30 min at 25 °C prior to cross-linking. Molecular weight markers (kDa) are indicated (maximum 250 kDa). Lanes 1-4: DMSO, Bis, Zp, Pol (−PIP_2_). Lanes 5-12: DMSO, Bis, Zp, Pol (+PIP_2_; conditions shown in duplicate). Monomeric ExoU migrates at ~74 kDa, with higher molecular weight species corresponding to dimeric (~150 kDa), tetrameric, and higher-order oligomeric complexes. **(C)** Combinatorial compound treatment enhances ExoU inhibition *in vitro*. Phospholipase activity of ExoU was measured in real-time assays in the presence of individual inhibitors (Bis, Zp, or Pol) or equimolar combinations of thereof. Dose-response curves were generated by fitting log[inhibitor] versus activity data using a three-parameter nonlinear regression model in GraphPad Prism. IC₅₀ were derived from the fitted curves to quantify the extent of ExoU inhibition.

After reacting ExoU with the homobifunctional cross-linker BS^3^, the protein migrated on SDS-PAGE as a smear around 74 kDa (Fig 3B). The smearing may have been the result of the reaction products, whereby BS^3^ may react with a single amino group (generally lysine side chains) or form a loop between two, so generating a range of products of different sizes. While Bis did not alter this profile, in the presence of Zp the ExoU migrated as a smear centred on a band >250 kDa, with only a small amount of monomer remaining. In contrast in the presence of Pol, ExoU migrated as a diffuse smear centred around ~100 kDa (Fig 3B). This likely represents a dimer which migrated anomalously due to BS^3^ cross linking.

In the presence of PIP_2_ (Fig 3B, lanes 5–12) BS^3^ crosslinking resulted in a dominant ExoU species migrating at ~300 kDa, with bands at ~400 kDa, ~ 150 kDa and ~74 kDa also present (Fig 3B, lanes 5–6).

In the presence of Bis and PIP_2_ (lanes 7–8) crosslinking of ExoU with BS^3^ resulted in a single discrete high‐molecular‐weight band matching the Zp (-PIP_2_) complex and a smear around ~74 kDa. The absence of lower‐order dimers or a smear around 300/400 kDa suggested that Bis constrained the PIP_2_‐induced assembly of ExoU into one oligomeric form. By contrast, in the presence of Zp and PIP_2_ (lanes 9–10) crosslinked ExoU migrated mainly as two uniform bands at ~300 kDa and >400 kDa, the latter being the more intense species and representing the bulk of crosslinked protein. Finally, in the presence of Pol and PIP_2_ (lanes 11–12) the smear observed in the absence of PIP_2_ migrated slower (~80–150 kDa), indicative of the presence of monomers and dimers.

### Compound combination enhances *in vitro* ExoU inhibition

Given that Bis, Pol, and Zp inhibited ExoU through distinct mechanisms, we hypothesized that combining these compounds could enhance inhibitory potency. To test this, we performed dose-response phospholipase assays in the presence of Bis, Zp, Pol, and equimolar compound combinations (Fig 3C). Individually, Bis, Zp, and Pol inhibited ExoU activity with IC₅₀ values of 13.3 ± 2.9 µM, 1.4 ± 0.2 µM, and 11.6 ± 0.9 µM. All pairwise combinations exhibited increased potency relative to single agents, with IC₅₀ values in the sub- to low-micromolar range. Notably, the triple combination displayed markedly enhanced inhibition, yielding an IC₅₀ of 0.48 ± 0.1 µM. These findings suggest that the compounds act through complementary mechanisms and provide a rationale for evaluating combination treatments in subsequent efficacy studies.

### Bis and Pol destabilise EGFP-tagged ExoU (S142A) expressed in HEK293T cells

**Fig 4:**
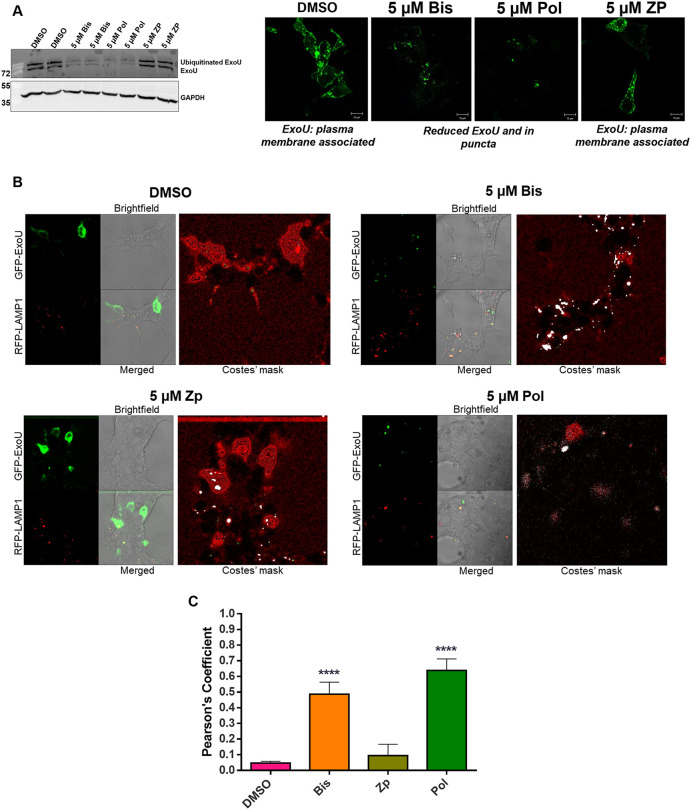
Bismuth subcitrate and polymyxin B promote lysosome localisation and degradation of GFP-S142A ExoU in HEK293T cells. **(A)** Left: HEK293T cells were transfected with pEGFP-C3, encoding S142A ExoU with an N-terminal EGFP tag, in the presence of bismuth subcitrate (Bis), polymyxin B (Pol) or zinc pyrithione (Zp) for 16 hours. Western blotting of whole cell lysates (two independent samples per group; three separate experiments) was used to detect abundance of GFP-S142A ExoU in transfected HEK293T cells, using an α-GFP antibody. Right: Confocal fluorescence microscopy was used to demonstrate plasma membrane distribution of GFP-S142A ExoU in the presence of DMSO and Zp but reduced in the presence of Bis and Pol (full z-stacks are available in supplementary). **(B)** HEK293T cells were co-transfected with GFP-S142A ExoU and the RFP-tagged lysosomal marker LAMP1, followed by 16 h compound treatment. Fluorescence microscopy with split-channel imaging (GFP, RFP, brightfield, and merged), together with Costes’ mask images (colocalised pixels shown in white), was used to visualise S142A ExoU-lysosome colocalization. **(C)** Quantification of ExoU-lysosome colocalization in the presence of DMSO, Bis Zp or Pol was performed from three independent experiments using the Coloc 2 plugin in Fiji (ImageJ) to calculate Pearson’s correlation coefficients. Data were plotted in GraphPad Prism, and statistical significance was assessed by one-way ANOVA with multiple comparisons to DMSO-treated controls. Original uncropped and unadjusted images underlying all blot and gel results are presented in S1 Raw Gel.

To examine the effects of Bis, Pol and Zp on ExoU in host cells, HEK293T cells were transfected with a plasmid encoding EGFP-tagged catalytically inactive ExoU (S142A), as WT is toxic to transfected cells. After 16 h transfection, detection of GFP-S142A ExoU by Western blotting and confocal fluorescence microscopy were used to establish inhibitor effects on ExoU abundance and localisation (Fig 4).

In HEK293T cells transfected with the plasmid encoding EGFP-tagged ExoU (S142A) produced consistent quantities of EGFP-tagged ExoU (S142A) (Fig 4A). The larger form was possibly ubiquitinated, consistent with previous studies, that found that ExoU undergoes modification within the cell by addition of two ubiquitin molecules at lysine-178 [33]. In transfected cells incubated with Zp, the amount of EGFP-ExoU (S142A) was similar. When the transfected cells were treated with 5 µM Bis or Pol, however, the level of EGFP-tagged ExoU (S142A) was substantially reduced (Fig 4A, left panel). Using confocal microscopy, EGFP-ExoU (S142A) fluorescence in control and Zp-treated cells was predominantly localised to the plasma membrane (Fig 4A, right panel). In contrast, treatment with Bis or Pol resulted in a marked reduction in EGFP–ExoU (S142A) fluorescence (S1 Video, S2 Video, S3 Video and S4 Video), consistent with the decreased protein abundance observed by Western blot (Fig 4A, left panel).

As we previously showed that ExoU turnover is mediated by PMSF-sensitive proteases rather than the proteasome [30], the reduction in ExoU levels suggested involvement of an alternative degradation pathway. Notably, prior studies have reported that ExoU (S142A) can traffic through endosomal and lysosomal compartments, where it accumulates in punctate structures [33, 34]. Based on these observations, we hypothesised that Bis and Pol promote trafficking of ExoU to lysosomes, leading to its degradation.

To test this, HEK293T cells were co-transfected with EGFP-ExoU (S142A) and RFP-tagged LAMP1, a lysosomal marker. In DMSO-treated cells, EGFP-ExoU (S142A) remained predominantly associated with the plasma membrane at 16 h post-transfection (Fig 4B). In contrast, Bis- or Pol-treated cells showed reduced membrane-associated fluorescence, with the remaining EGFP-ExoU (S142A) redistributed into punctate structures that co-localised with RFP-LAMP1 (Fig 4B), consistent with lysosomal trafficking.

Analysis of the images showed that Bis and Pol treatments (Pearson’s correlation coefficient r = 0.49 and r = 0.64, respectively) substantially increased colocalisation of ExoU with lysosomes compared with the DMSO control (Pearson’s correlation coefficient r = 0.05) (Fig 4C). In contrast, Zp treatment did not enhance colocalisation (Pearson’s correlation coefficient r = 0.09). Taken alongside the differences observed in the effects of each compound on the Tₘ of ExoU (Fig 3A), on the complexes observed upon BS^3^ crosslinking (Fig 4B) and the enhanced inhibition seen when the compounds were used together (Fig 3C), these data support the argument that the compounds inhibit ExoU through complementary mechanisms.

### Efficacy of ExoU inhibitors in a high-content microscopy screen of clinical *P. aeruginosa* keratitis isolates

Whereas Bis, Pol, and Zp reduce ExoU-mediated cytotoxicity in cell infection models using the laboratory PA103 strain, it was important to determine if they did likewise with clinically relevant strains. To this end we employed a fully sequenced, phenotyped, and genotyped library of 52 *P. aeruginosa* clinical keratitis isolates from the UK Microbiology Ophthalmic group [35]. High-throughput phenotypic screening used a CellDiscoverer 7 (CD7) platform for live-cell imaging.

**Fig 5:**
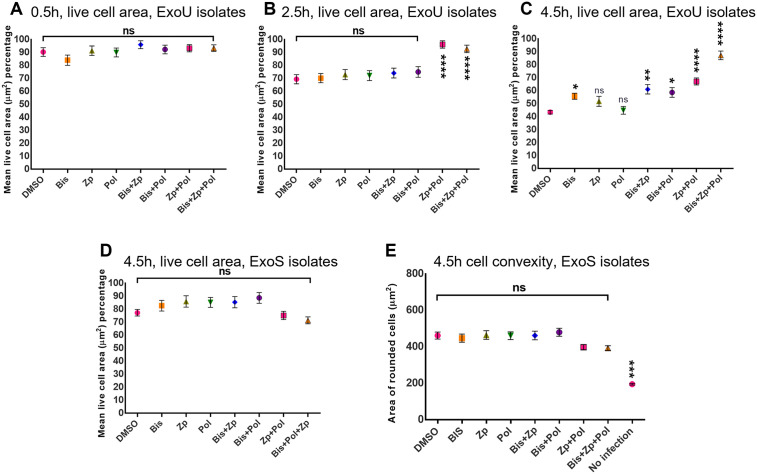
High-content imaging screen evaluating ExoU inhibitor efficacy across clinical *P. aeruginosa* keratitis isolates. A high-content microscopy screen using the Cell Discoverer 7 (CD7) platform was developed to assess the efficacy of ExoU inhibitors against PA103 and a panel of 29 *exoU*⁺ and 24 *exoS*⁺ clinical *P. aeruginosa* keratitis isolates. HCE-T cells were seeded into 96-well plates and cultured to ~80% confluence prior to infection at MOI 10. Cells were treated with 10 µM Bis, 2.5 µM zinc pyrithione (Zp), 1 µM polymyxin B (Pol), or the indicated combinations (Bis + Zp, Bis + Pol, Zp + Pol, and Bis + Zp + Pol). Cells were stained with calcein-AM and ethidium homodimer to quantify cell viability and cytotoxicity, enabling assessment of cell lysis (ExoU activity) or cell rounding (ExoS activity) over 4.5 **h. (A-C)** Mean live-cell area at 0.5, 2.5, and 4.5 h post-infection, expressed as a mean percentage across all isolates (± standard error of mean) relative to non-infected controls (live-cell area, µm^2^). **(D)** Live-cell area percentage across all conditions following infection with *exoS*⁺ clinical isolates, which do not induce rapid cell lysis and therefore serve as controls for ExoU-dependent cytotoxicity. **(E)** Quantification of cell rounding following infection with *exoS*⁺ clinical isolates at 4.5 h post-infection, expressed as rounded-cell area (convexity, µm^2^). For individual strain data, see S9 Fig. Statistical analysis was performed using one-way ANOVA with multiple-comparisons testing in GraphPad Prism, comparing each condition to infected vehicle (DMSO) controls.

HCE-T cells were seeded in 96-well plates, stained with Calcein-AM and ethidium homodimer, and infected at ~80% confluence with each clinical isolate (MOI 10) in the presence of individual inhibitors (Bis, Pol or Zp) and combinations thereof (Bis + Zp, Bis + Pol, Zp + Pol and Bis + Zp + Pol) (Fig 5). Importantly, none of the compounds, alone or in combination, affected bacterial viability at the assayed concentrations (S8 Fig).

Time-lapse imaging was performed every 0.5 h over 4.5 h. Among the 52 isolates, 28 were *exoU⁺* and 24 *exoS ⁺* . For nearly all *exoU⁺* strains, ExoU-mediated cytolysis was evident as progressive loss of Calcein-AM fluorescence and membrane rupture (S5 Video). The sole exception was isolate 68228, which displayed fewer lysed cells than uninfected controls (S9 Fig), indicating an absence of ExoU-dependent cytotoxicity. In contrast, *exoS⁺* isolates induced cell rounding while maintaining Calcein-AM signal, consistent with cytoskeletal disruption but preserved membrane integrity (S1 Video 6).

### Compound efficacy in ExoU producing clinical P. aeruginosa clinical isolates

To quantify inhibitor efficacy across the *exoU*⁺ cohort, live-cell area was measured over time and normalised to non-infected controls to account for inter-experimental variability (Fig 5A-C).

At early timepoints (0.5 h post-infection), minimal cytotoxicity was observed, with no significant differences between treatment conditions (mean live-cell area ~90% of non-infected controls; Fig 5A). By 2.5 h, cytotoxic effects became apparent (DMSO ~69%), with significant protection observed only for the combination treatments Zp + Pol and the triple combination (Bis + Zp + Pol), both of which showed significant improvements in cell viability (Fig 5B).

At 4.5 h post-infection, pronounced ExoU-dependent cytotoxicity was evident in DMSO-treated cells (~43% live-cell area). Of the individual compounds, only Bis conferred modest protection (55.8%, P < 0.05), whereas combination treatments resulted in progressively enhanced efficacy. Zp + Pol significantly improved cell viability (67.2%, P < 0.0001), while the triple combination (Bis + Zp + Pol) provided the greatest protection (87.3%, P < 0.0001), approaching levels observed in non-infected controls (Fig 5C). The other combinations (Bis + Zp and Bis + Pol) also showed significant, but less pronounced, effects, consistent with additive or complementary activity.

To more accurately assess the breadth of inhibitor efficacy across the *exoU*⁺ clinical isolate panel, we analysed live-cell area at the 4.5 h endpoint for each strain under all treatment conditions (S9 Fig). Substantial heterogeneity in ExoU-mediated cytotoxicity was observed across isolates, with variable responses to individual compounds. Notably, Zp retained unique activity against isolate 68130, which was unresponsive to other single-agent treatments. For the reference strain PA103, however, Zp and Pol alone did not confer significant protection (S9 Fig), in contrast to the scratch assay (Fig 1D). This likely reflects differences in assay format, with the high-content assay using sub-confluent cells and live-cell area as a readout, versus the scratch assay measuring acute lysis in fully confluent monolayers.

Combination therapy improved both the magnitude and breadth of protection. Dual combinations (Bis + Zp and Bis + Pol) increased the proportion of responsive isolates to 60%, while Zp + Pol provided further enhancement (70%), consistent with its strong efficacy in the primary screen.

Inclusion of Bis in the triple combination (Bis + Zp + Pol) yielded the most robust and consistent protective effect, increasing responder rates to 85% of isolates. Across many strains, live-cell area approached levels observed in non-infected controls, indicating near-complete suppression of ExoU-mediated cytotoxicity. In addition, the triple combination reduced inter-isolate variability and converted several previously non-responsive strains (76230, 86028, 68130, and 68168) into responders, demonstrating expanded coverage across the clinical panel.

Despite this, a small subset of isolates remained only weakly responsive, suggesting intrinsic differences in ExoU activity, expression, or host-pathogen interactions. Nevertheless, these data demonstrate that combination therapy, particularly the triple regimen, substantially enhances both the efficacy and consistency of ExoU inhibition across genetically diverse clinical isolates.

### Bis, Pol and Zp do not effectively reduce ExoS mediated cytotoxicity

Whereas the three compounds inhibited ExoU activity, it was possible that they also interfered with type III secretion. Therefore, we examined the 24 *exoS*⁺ isolates in the panel of clinical isolates and quantified host cell viability (Fig 5D), but more importantly, host cell convexity (Fig 5E) as a readout of ExoS-mediated cytotoxicity. ExoS promotes cell rounding through its GAP and ADP-ribosyltransferase domains, which inactivate Rho family GTPases, disrupt actin polymerisation and focal adhesion dynamics, and thereby increase cell convexity [36,37] (S2 Video).

No significant differences in live-cell area were observed across treatment conditions at 4.5 h (Fig 5D), indicating that the compounds do not broadly affect host cell survival under these conditions. Furthermore, quantification of cell rounding (convexity) demonstrated that compound treatment did not inhibit ExoS-mediated morphological changes (Fig 5E), supporting a mechanism specific to ExoU inhibition.

In addition, we evaluated whether Bis, Zp or Pol affected T3SS gene expression in *P. aeruginosa* PA103 and PAO1 [30]. Quantitative RT-PCR analysis (S10 Fig) revealed no significant changes in the expression of key T3SS-associated genes *exsA*, *pcrV*, and *exoU* in PA103, or *exsA*, *pcrV*, and *exoS* in PAO1, following treatment with the compounds at concentrations used in the cell-based assays. These results indicated that the compounds did not suppress the transcription of genes associated with T3SS or of the two exotoxins, further supporting that their protective effects observed in cell assays were due to direct inhibition of ExoU activity.

### Evaluation of ExoU inhibitors in a porcine *ex vivo* corneal infection model

Our previously developed *ex vivo* porcine corneal infection model [38] provided a means to measure the efficacy of Bis, Zp and Pol to mitigate *P. aeruginosa* PA103-induced damage in a living cornea. The ring-based setup allowed sustained exposure of dissected porcine corneas to both compound and pathogen at the infection site. Infection severity was quantified through measurements of corneal opacity, epithelial ulceration, bacterial burden (CFU per cornea), and further assessed by histological examination.

**Fig 6:**
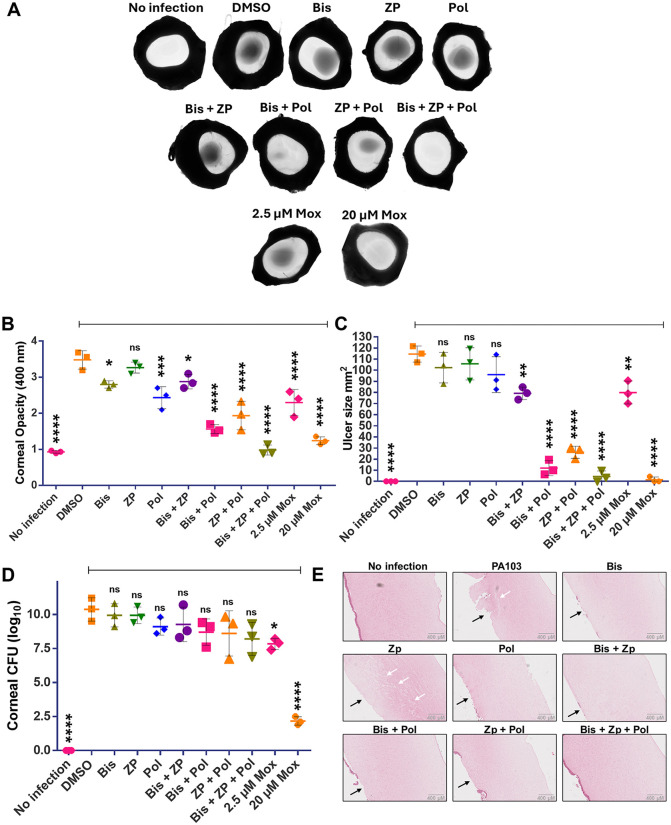
ExoU inhibitor combinations reduce *P. aeruginosa p*athogenicity in *ex vivo* porcine corneas. **(A)** Dissected porcine corneas were infected with 1 × 10⁵ CFU of PA103, followed by topical application of ExoU inhibitors individually (Bis: 5 µM, ZP: 2.5 µM, Pol: 1 µM) or in combination. A 20 µM moxifloxacin treatment served as a positive control. Corneas were imaged after 48 hours of infection using a BioRad ChemiDoc imaging system. **(B)** Corneal opacity caused by ulceration 48 hours post-infection was quantified by measuring optical density at 400 nm using a spectrophotometer. **(C)** The ulcerated area (mm^2^) of infected corneas was measured at 48 hours post-infection using ImageJ. **(D)** At 48 hours post-infection, corneas were washed with PBS, homogenized, and serially plated onto agar plates to determine the bacterial load (CFU) within the corneal tissue. **(E)** Infected corneas were fixed in paraformaldehyde (PFA), paraffin-embedded, sectioned, and stained with haematoxylin and eosin **(H&E)**. Sections were imaged using a Ventana DP 200 slide scanner. Black arrows indicate areas of epithelial erosion/ulceration; white arrows highlight regions of stromal oedema.

Dissected porcine corneas were topically infected with 50 µL solutions of *P. aeruginosa* strain PA103 (1 × 10⁵ CFU) mixed and treated topically with 50 µL solutions of Bis (5 µM), Zp (2.5 µM), or Pol (1 µM), either alone or in combination. After 48 hours, monotherapies had only modest effects on reducing corneal opacity, with Bis performing slightly better than Zp (Fig 6A). Combinatorial treatments yielded progressively improved outcomes: Bis + Zp showed minor improvement over single-agent treatments, while Zp + Pol and Bis + Pol produced greater reductions in opacity. Notably, the triple combination (Bis + Zp + Pol) resulted in corneas that closely resembled uninfected controls, suggesting near-complete protection (Fig 6A).

These qualitative observations were supported by quantitative measurement of corneal opacity (Fig 6B). While individual compounds modestly reduced opacity compared to DMSO-treated infected controls, combinations, particularly Bis + Pol and the Bis + Zp + Pol treatment, produced significantly greater improvement. The triple combination was comparable to 20 µM moxifloxacin, a clinically relevant positive control. Assessment of epithelial ulceration (Fig 6C), defined here as the combined loss of epithelial integrity and underlying stromal degradation following infection, mirrored these trends, mirrored these trends. Corneal damage was substantially reduced in combination treatments, with Bis + Pol and Bis + Zp + Pol showing the most dramatic reductions in ulcer size, approaching the resolution achieved with high-dose moxifloxacin. Bacterial loads within the corneas, measured by CFU (Fig 6D), were not significantly altered by compound treatment, indicating that ExoU inhibitors were not directly bactericidal under these conditions. As expected, 20 µM moxifloxacin markedly reduced bacterial burden but did not fully eradicate infection, consistent with previous reports [38].

Histological analysis of haematoxylin and eosin-stained sections (Fig 6E) corroborated these findings. Although the epithelium was initially partially disrupted during model establishment, DMSO-treated infected corneas showed complete epithelial loss, severe stromal erosion, and oedema. In contrast, Bis and Pol treated corneas showed partial epithelial preservation and less stromal damage. The triple combination conferred near-complete epithelial integrity and minimal stromal swelling, closely resembling uninfected tissue.

Together, these data demonstrated that the three ExoU inhibitors, particularly in combination, could significantly reduce the severity of *P. aeruginosa* PA103-induced keratitis in an *ex vivo* porcine model, even without directly affecting bacterial viability.

### Bis, Zp and Pol enhance survival of PA103 infected *Galleria mellonella* larvae

**Fig 7:**
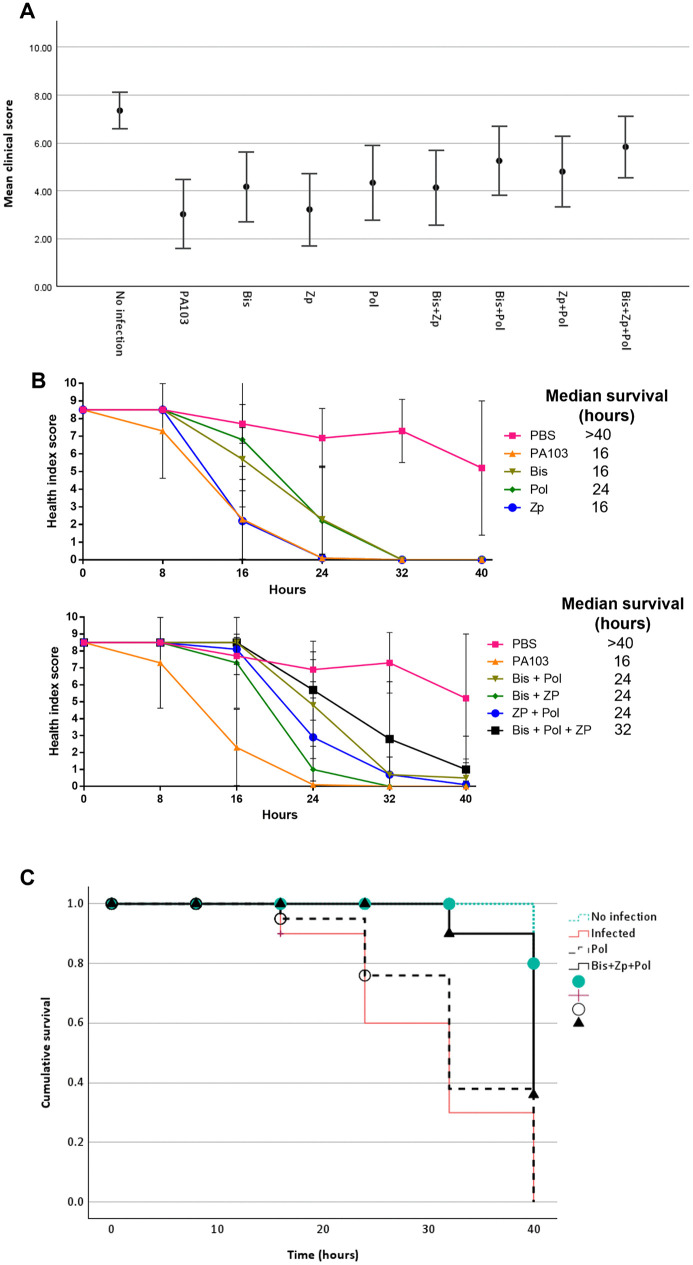
ExoU inhibitors enhance survival of *P. aeruginosa* PA103-infected *Galleria mellonella.*

To assess the therapeutic potential of ExoU inhibitors in vivo, *Galleria mellonella* larvae were infected with 1 × 10⁵ CFU of PA103 and treated with individual compounds or combinations thereof. Larval health was evaluated using a validated scoring system (0–9) [39], as cocoon formation was not observed within the 40 h experimental window. Mean health scores at 40 h post-infection were analysed using linear mixed-effects models with repeated measures and post hoc Tukey testing (Table 1; Fig 7A). In addition, larval health was monitored at 8 h intervals (Fig 7B), and survival was assessed by Kaplan–Meier analysis (Fig 7C).

**Table 1:** *Galleria* mean ± SD clinical health scores. The health scores of *Galleria* larvae (5 per group) were analysed using a validated (0-9) scoring system, across 40h of infection. A linear mixed model was used with repeated measures and post hoc Tukey tests. Statistical differences between all groups are shown (p < 0.05*).

	Clinical score: mean±SD	Condition							
		Bis	Zp	Pol	Bis + Zp	Bis + Pol	Zp + Pol	Bis + Zp + Pol	DMSO
**No infection (0.01% DMSO)**	7.35 ± 2.07	<0.01	<0.01	<0.01	<0.01	0.02	<0.01	0.27	<0.01
**Bis**	4.17 ± 3.89		0.54	1.00	1.00	0.27	0.89	<0.01	0.22
**Zp**	3.22 ± 4.05			0.49	0.70	<0.01	0.04	<0.01	1.00
**Pol**	4.33 ± 4.20				1.00	0.70	0.99	0.10	0.23
**Bis + Zp**	4.13 ± 4.20					0.36	0.91	0.02	0.39
**Bis + Pol**	5.25 ± 3.83						0.99	0.96	<0.01
**Zp + Pol**	4.80 ± 3.96							0.46	<0.01
**Bis + Zp + Pol**	5.83 ± 3.44								<0.01
**PBS (0.1% DMSO)**	3.21 ± 4.05								

*Galleria mellonella* larvae were infected with 1 × 10⁵ CFU of *P. aeruginosa* PA103 and treated with ExoU inhibitors or vehicle control (PBS + 0.1% DMSO). Treatments included bismuth subcitrate (Bis; 0.1 mg/g), zinc pyrithione (Zp; 0.01 mg/g), polymyxin B (Pol; 0.004 mg/g), or combinations thereof. Data represent five independent experiments (mean of 2 larvae per condition per experiment; total n = 10 per condition). Larval health was assessed using a modified 0–9 health index scoring system (maximum score reduced from 10 due to absence of cocoon formation within the 40 h assay period). (A) Mean ± SD health scores at 40 h post-infection. (B) Longitudinal health scores (mean ± SD) recorded at 8 h intervals. Top panel: single-agent treatments; bottom panel: combination treatments. (C) Kaplan–Meier survival curves showing the proportion of surviving larvae over time for vehicle control, polymyxin B alone, and the triple combination (Bis + Zp + Pol). Statistical analysis was performed using linear mixed-effects models with repeated measures and Tukey’s post hoc test (A,B), and log-rank (Mantel–Cox) test for survival (C). Significance thresholds: p < 0.05 (*), p < 0.01 (**), p < 0.001 (***), p < 0.0001 (****).

PBS-injected controls maintained relatively high health scores throughout the experiment (mean 7.4 ± 2.0), with a modest decline over time likely reflecting injection-associated stress rather than infection. In contrast, PA103 infection caused rapid deterioration in larval health, resulting in complete mortality by 32 h (Fig 7A-C). Consistent with this, infection significantly reduced health scores in mock-treated larvae (PBS + 0.1% DMSO; p < 0.01), as well as in all single-agent (Bis, Zp, Pol) and dual-treatment groups (Bis + Zp, Zp + Pol, Bis + Pol; p < 0.01) when compared to uninfected controls.

Single-agent treatments provided only modest and transient benefit. Bis (0.1 mg/g) and Zp (0.01 mg/g) delayed disease progression by approximately 8 h in a subset of larvae, while Pol (0.004 mg/g) showed slightly greater efficacy, with some larvae surviving beyond 24 h; however, none of the individual treatments prevented mortality by 40 h (Fig 7B).

Dual combinations improved outcomes relative to single agents but did not fully prevent disease progression. Bis + Zp delayed larval decline, though mortality still occurred by 32 h. Zp + Pol extended survival further, with some larvae retaining partial activity up to 40 h, while Bis + Pol provided the most consistent protection among dual treatments, with a small number of larvae maintaining measurable health scores at 40 h (Fig 7B).

The triple combination (Bis + Zp + Pol) conferred the greatest benefit. Larvae maintained high health scores through 16 h, followed by a progressive decline, although this was delayed relative to all other treatment groups. Several larvae retained moderate health scores at 24 h, and one larva maintained a score of 4.5 at 40 h (Fig 7B). At the 40 h endpoint, mean health scores for the triple combination (5.8 ± 3.4) did not reach statistical significance compared to uninfected controls (p = 0.27); however, variability between larvae remained high and outcomes did not fully match baseline health. Kaplan–Meier analysis supported an overall improvement in survival relative to infection alone (Fig 7C), but not complete restoration to uninfected levels.

Together, these data indicate that combination therapy, particularly the triple regimen, improves larval health and survival in this model, although protection remains partial under these conditions.

### ExoU inhibitor combinations improve clinical outcome in an *in vivo* mouse model of keratitis

**Fig 8:**
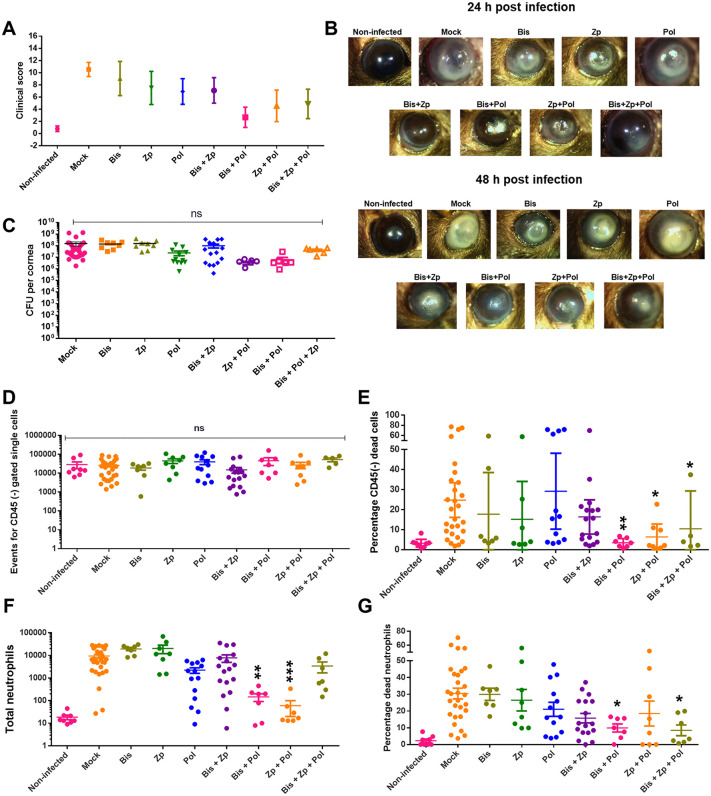
ExoU inhibitor combinations reduce disease severity and bacterial burden in a murine *P. aeruginosa* corneal infection model. Corneal infections were established in female C57BL/6J mice (8–10 weeks old) by creating three parallel scratches with a 25-gauge needle, followed by topical inoculation with 1 × 10⁴ CFU of *P. aeruginosa* strain PA48386. After 30 min, mice received topical treatment with ExoU inhibitors Bis (50 µM), Zp (20 µM), Pol (1 µM) or PBS vehicle control (5 µL per cornea). Treatments were repeated at 4 h and 24 h post-infection. Eyes were imaged at 24 h and 48 h, and corneas collected at 48 h for analysis. **(A)** Clinical severity of corneal infection at 48 h, scored on a standardized 16-point scale by two independent, masked assessors. Bar graphs show severity scores (mean and ±SD). (**B**) representative corneal images are shown below for each treatment at 24 h and 48 **h. (C)** Bacterial burden (CFU) in whole corneal homogenates at 48 **h. (D-G)** Flow cytometry of corneal single-cell suspensions. **(D)** Quantification of non-immune cells (CD45^−^). **(E)** Proportion of dead non-immune cells (Zombie Violet⁺). **(F)** Quantification of neutrophils (CD45 ⁺ Ly6B⁺F4/80^−^). **(G)** Proportion of dead neutrophils across treatment groups. Statistical significance for D-G was assessed by one-way ANOVA with post hoc correction for multiple comparisons against mock-treated controls. *p < 0.05, **p < 0.01, ***p < 0.001.

To evaluate the therapeutic potential of ExoU inhibitors during *P. aeruginosa* corneal infection, we used a murine scratch and infection model [40,41]. Mice received topical treatment with individual inhibitors or inhibitor combinations at 30 min, 4 h, and 24 h post-infection. Disease severity was evaluated at 24 and 48 h using a standard 16-point clinical scoring system (Fig 8A), with representative corneal images shown at 24 and 48 h (Fig 8B). Bacterial load was quantified by CFU enumeration (Fig 8C). In parallel, flow cytometric analysis of digested corneas was performed to assess host responses, including corneal cell viability, total leukocyte infiltration, and neutrophil recruitment (Fig 8D-G).

### Combination therapy, but not single agents, significantly reduced disease severity

Non-infected corneas exhibited minimal pathology, whereas mock-treated infected corneas showed severe disease (mean clinical score 10.53 at 24 h; Fig 8A; Table 2). Treatment with individual inhibitors (Bis, Zp, or Pol) did not significantly improve clinical scores at either 24 h or 48 h (Tables 2 and 3). In contrast, combination therapies significantly reduced disease severity at both timepoints, with Bis + Pol and Zp + Pol showing the greatest benefit. The triple combination (Bis + Zp + Pol) also improved outcomes compared to mock-treated controls, although it did not confer a significant advantage over the most effective dual combinations (Table 3).

**Table 2:** Clinical severity scores at 24 hours post-infection in a murine *P. aeruginosa* corneal infection model. Female C57BL/6J mice (8–10 weeks old) were scratched and topically inoculated with *P. aeruginosa* strain PA48386 (1 × 10⁴ CFU per cornea). Mice received topical treatment with ExoU inhibitors Bis (50 µM), Zp (20 µM), Pol (1 µM), or combinations thereof at 30 min, 4 h, and 24 h post-infection. Control animals received PBS with 0.1% (v/v) DMSO. Corneal images were captured 24 h post-infection and graded by two masked observers using a standardized 16-point scoring system. Values represent mean ± SD (median and interquartile range (IQR) from two independent, masked assessments. Statistical analysis was performed using a General Linear Model (GLM) followed by Tukey’s post hoc test (*p* < 0.05). The probability values for comparison of each condition are provided. Combination treatments reduced clinical severity compared to mock treatment, with the lowest scores observed in the Bis + Pol and Zp + Pol groups.

	Clinical score: mean ±SD (median, IQR) at 24h	Condition							
		Bis	Zp	Pol	Bis + Zp	Bis + Pol	Zp + Pol	Bis + Zp + Pol	No treatment
**No infection (n = 20)**	0.17 ± 0.35 (0, 0.13)	<0.01	<0.01	0.14	0.001	0.64	0.93	0.94	<0.01
**Bis (n = 16)**	4.99 ± 3.60(5.50, 5.69)		0.25	0.18	0.99	0.24	0.06	0.02	0.99
**Zp (n = 18)**	7.25 ± 1.96 (7.00, 2.13)			<0.01	0.007	<0.01	<0.01	<0.01	0.41
**Pol (n = 24)**	2.92 ± 2.46 (2.88, 4.56)				0.43	1.00	0.95	0.64	<0.01
**Bis + Zp (n = 29)**	4.30 ± 3.08 (4.63, 5.00)					0.52	0.15	0.005	0.18
**Bis + Pol (n = 16)**	2.45 ± 2.22 (2.50, 3.50)						1.00	0.99	0.007
**Zp + Pol (n = 10)**	1.75 ± 2.21 (1.55, 2.75)							1.00	<0.01
**Bis + Zp + Pol (n = 18)**	1.50 ± 1.54 (1.75, 2.75)								<0.01
**No treatment (n = 58)**	5.65 ± 2.83 (5.50, 3.25)								

**Table 3:** Clinical severity scores at 48 hours post-infection in a murine *P. aeruginosa* corneal infection model. Clinical scores were assessed 48 h post-infection in the same experimental cohort described in Table 2. Each image was independently graded twice by two masked observers (Cohen’s weighted κ = 0.74–0.84, indicating strong inter- and intra-rater agreement). Data represent mean ± SD (median and interquartile range (IQR). Statistical analysis was performed using a General Linear Model (GLM) with Tukey’s post hoc test (*p* < 0.05). The probability values for comparison of each condition are provided. Compared to mock-treated infected controls, combination treatments (Bis + Pol, Zp + Pol, Bis + Zp + Pol) significantly improved clinical outcome, achieving scores comparable to uninfected controls.

	Clinical score: mean at 48h Mean SD (Median, IQR)	Condition (treatment):							
Number of mice per group ()		Bis	Zp	Pol	Bis + Zp	Bis + Pol	Zp + Pol	Bis + Zp + Pol	No treatment
**No infection (n = 20)**	0.80 ± 0.96 (0.38,1.54)	<0.01	<0.01	<0.01	<0.01	0.95	0.46	0.13	<0.01
**Bis (n = 16)**	9.05 ± 5.27 (10.75(8.50)		0.99	0.87	0.90	0.003	0.26	0.17	0.97
**Zp (n = 18)**	7.50 ± 5.48 (7.38, 10.38)			1.00	1.00	0.06	0.78	0.73	0.25
**Pol (n = 24)**	6.91 ± 5.00 (6.50, 9.81)				1.00	0.10	0.91	0.88	0.03
**Bis + Zp (n = 29)**	7.09 ± 5.50 (7.75, 9.25)					0.053	0.84	0.79	<0.03
**Bis + Pol (n = 16)**	2.67 ± 3.13 (1.50,4.63						0.98	0.89	<0.01
**Zp + Pol (n = 10)**	4.55 ± 3.61 (5.50,6.81)							1.00	0.005
**Bis + Zp + Pol (n = 18)**	4.88 ± 4.86 (3.13, 9.00)								<0.01
**No treatment (n = 58)**	10.53 ± 4.40(12.13, 6.69)								

### Therapeutic benefit occurred independently of bacterial clearance

Bacterial burden, quantified by CFU at 48 h, was not significantly different between treatment groups (Fig 8C), indicating that the observed improvements in clinical outcome were not due to direct antibacterial effects.

### Combination therapy preserves corneal cell viability and modulates neutrophil responses

Flow cytometric analysis revealed no significant differences in total CD45^−^ cell numbers across groups (Fig 8D), indicating comparable tissue recovery. However, combination treatments (Bis + Zp, Zp + Pol, and Bis + Zp + Pol) significantly reduced the proportion of dead CD45^−^ cells relative to mock-treated controls (Fig 8E), consistent with protection of corneal epithelial cells.

Analysis of immune infiltration showed that neutrophil numbers were significantly reduced in the Bis + Pol and Zp + Pol groups (Fig 8F). In addition, the proportion of dead neutrophils was decreased in the Bis + Pol and triple combination groups (Fig 8G), suggesting improved neutrophil viability.

## Discussion

Microbial keratitis is a significant cause of blindness [42] with *P. aeruginosa*, in particular the ExoU positive strains, being a major pathogen [8]. Outcomes remain poor despite antimicrobials due in part to the bacterial toxins produced. Although *P. aeruginosa* produces several toxins, ExoU accounts for a significant morbidity. Targeting the phospholipase activity of ExoU in the host cell, therefore, would be a reasonable strategy [8,25]. In this study we incorporated further steps into our pipeline for the discovery of ExoU inhibitors. The first high throughput real-time-time phospholipase assay [25] was followed by a high content co-infection assay of *P. aeruginosa* cytotoxicity using the Incucyte and the CD7 platforms, which were more robust and higher throughput than the manual scratch wound assay used previously [25]. These were complemented by measurement of compound inhibitory action on the *ex vivo* porcine corneal model, on pathogen lethality in *Galleria* and on a murine model of microbial keratitis. This integrated pipeline, combining *in vitro* and cell-based analyses of ExoU inhibition, enabled the identification of Bis, Pol, and Zp as safe and effective inhibitors that significantly reduced ExoU-mediated corneal damage in mice. Moreover, the counter screens against human PLA_2_G7 and PLA_2_G4C (S4 Fig), demonstrated their ExoU selectivity, which reduces concerns over collateral impairment of host lipid signalling or membrane homeostasis, as found for some phospholipase inhibitors, such as darapladib [43].

The analysis of the likely pharmacophores of the three compounds demonstrated that the metal cations Bi^3+^ and Zn^2^ ^+^ were active inhibitors, either alone or as chelated species, *in vitro* (Fig 2B and 2C). In the case of polymyxin B, peptide length, charge complementarity, and lipid anchoring were essential for ExoU inhibitory activity. In HCE-T cells, Bis was not cytotoxic at the concentrations tested, whereas Zp and Pol exhibited measurable toxicity, with LD₅₀ values of 33 µM and 43 µM, respectively (S5 Fig). Notably, these concentrations remain below those reported to be tolerated *in vivo*. Zinc salts, particularly zinc sulphate, are widely used in ophthalmic formulations as mild astringents or antiseptics [44,45]; however, zinc sulphate alone did not reduce ExoU-mediated cytotoxicity in our scratch infection assay (S6 Fig). This suggests that intracellular delivery of Zn^2^ ^+^ is required for activity, consistent with the known ionophore properties of zinc pyrithione, which facilitates cellular zinc uptake [46].

While we did not directly measure intracellular accumulation, the requirement for a zinc carrier, together with the observed activity of Zp in host cell-based assays, supports a model in which Zp delivers Zn^2+^ into host cells to inhibit ExoU following translocation. We cannot exclude the possibility that Zp also interacts with ExoU within the bacterial cell prior to secretion. This is, however, less likely to be the dominant mechanism, as Bis, Zp, and Pol did not alter T3SS gene expression (S10 Fig), and no reduction in bacterial viability was observed under the conditions used. Together, these data are most consistent with inhibition of ExoU activity within the host cell. We cannot, however, exclude contributions from additional bacterial virulence determinants or host cell responses, particularly given the multi-parameter nature of the infection models used.

Commercial products containing zinc sulphate or zinc gluconate are marketed in several countries, often in combination with vasoconstrictors or soothing excipients. Bismuth containing ointments, such as bibrocatherol, are currently used in the management of eyelid inflammation [47] and polymyxin B is formulated in eye drops to treat microbial keratitis [48]. Direct clinical comparisons, however, between polymyxin B and commonly used first-line agents such as fluoroquinolones or aminoglycosides (e.g., tobramycin) in *P. aeruginosa*-specific keratitis are limited. Differences in pharmacokinetics, MIC distributions, and isolate-dependent susceptibility further complicate direct comparisons across antibiotic classes.

These considerations are particularly relevant in the context of the present study, where polymyxin B is not used for its bactericidal activity but rather as part of an antivirulence strategy targeting ExoU. In this setting, interactions between agents may range from additive to antagonistic, depending on both the bacterial strain and treatment conditions. Consistent with this, we maintained polymyxin B below its MIC to minimise confounding antimicrobial effects (S3 Fig).

The concentrations used in cell assays were below cytotoxic thresholds and within ranges considered safe *in vivo*. Consistent with this, increasing Zp to 20 µM *in vivo* produced no overt toxicity, while Pol was maintained below its MIC to avoid antimicrobial effects (S3 Fig). Although the safety of the triple combination remains to be established, each compound is individually well tolerated at higher concentrations, suggesting scope for dose optimisation.

Mechanistically, the compounds act through complementary pathways. Zinc-mediated active-site inhibition, bismuth-driven destabilisation, and peptide–lipid interactions, supporting their combined use. Notably, Bis and Pol also promote degradation of ExoU, indicating a dual mode of action involving both inhibition and toxin clearance. It may be that Bis and Pol amplify the known routing of ExoU to endosomes [33,49]. It cannot, however, be excluded that off-target effects of Bis and Pol cause a general upregulation of autophagy in the cells. Taken together, these data provided an important mechanistic framework for rational optimisation of ExoU inhibitors as a multicomponent formulation.

The analysis of compound efficacy across a panel of 52 clinical *P. aeruginosa* keratitis isolates provides important insight into the translational robustness of ExoU-targeted inhibition. While the laboratory strain PA103 offers a controlled proof-of-concept, clinical isolates capture the genetic and phenotypic diversity encountered in patients [35]. The high-content microscopy screen first demonstrated that the compounds are selective, with minimal impact on the 24 *exoS*⁺ isolates, consistent with a mechanism targeting ExoU-dependent cytotoxicity. In contrast, substantial heterogeneity was observed across *exoU*⁺ strains. Single-agent treatments conferred protection in approximately 45–55% of isolates, highlighting intrinsic variability in susceptibility.

Combination therapy improved both the magnitude and breadth of protection. Dual combinations (Bis + Zp, Bis + Pol, and Zp + Pol) increased coverage to ~70% of *exoU*⁺ isolates, with Zp + Pol showing the greatest efficacy among pairs. Notably, inclusion of all three compounds (Bis + Zp + Pol) further enhanced both the consistency and extent of protection, increasing the proportion of responsive isolates to ~85% and, in many cases, restoring cell viability to near non-infected levels. These findings demonstrate that while single agents exhibit variable efficacy across clinical isolates, combination therapy, particularly the triple regimen, substantially improves the breadth and robustness of ExoU inhibition.

In the porcine corneal infection model the Bis, Zp, nor Pol monotherapies did not fully prevent PA103 induced opacity or ulceration, whereas combination treatments, particularly Bis + Pol and the triple regimen Bis + Zp + Pol, markedly improved corneal clarity and epithelial integrity, achieving near complete protection comparable to high dose moxifloxacin (Fig 6). The absence of significant reductions in bacterial burden demonstrated that these inhibitors acted through antivirulence rather than bactericidal mechanisms, so preserving host tissue without driving pathogen clearance. This decoupling of therapeutic effect from antimicrobial activity is likely advantageous, as it may avoid selective pressures that contribute to antibiotic resistance [50].

While antivirulence approaches are often considered less prone to resistance than conventional antibiotics, resistance cannot be excluded. Mutations in components of the type III secretion system, such as the needle protein PscF, have been shown to confer resistance to small-molecule T3SS inhibitors [51].The compounds described here target ExoU activity within the host cell rather than the secretion apparatus itself, and act through complementary mechanisms, including disruption of oligomerisation and altered intracellular trafficking. This multi-target, host-directed mode of action may increase the barrier to resistance compared to single-target inhibitors. Nevertheless, the potential for adaptive responses, such as altered toxin expression, secretion dynamics, or host-pathogen interactions, will require further investigation.

The *Galleria mellonella* infection studies provided an *in vivo* proof‐of‐concept for ExoU inhibitor efficacy in a living host (Fig 7). Monotherapies with Bis, Zp, or Pol each only modestly delayed larval mortality, combination regimens, particularly Bis + Pol and the triple Bis + Zp + Pol cocktail significantly prolonged survival and health scores (Fig 7). These findings mirror our *ex vivo* corneal data and underscored the advantage of a therapeutic formulation consisting of all three compounds.

The murine scratch and-and infection model of *P. aeruginosa* keratitis provided key elements of the immune response, tear dynamics, and *in vivo* pharmacokinetics [38]. Consistent with *ex vivo* and *Galleria* results, individual treatments with Bis, Zp, or Pol failed to significantly reduce clinical scores or improve corneal pathology (Fig 8A). In contrast, combination regimens, particularly Bis + Pol and Zp + Pol, yielded marked reductions in clinical severity by 48 h, approaching scores observed in uninfected controls. The triple combination (Bis + Zp + Pol) further enhanced corneal protection, albeit without statistical advantage over the best dual therapies. Again, bacterial CFU counts remained unchanged across all inhibitor conditions, confirming an antivirulence rather than bactericidal mechanism (Fig 8C). Flow cytometry of digested corneas revealed that dual and triple inhibitor treatments preserved viability of epithelial cells included in the CD45^−^ population and reduced neutrophil infiltration and death, an indication that inhibition of ExoU mediated cytotoxicity helped maintain both barrier integrity and innate immune cell function (Fig 8D-G). The sparing of neutrophils, which play a key role in bacterial clearance and tissue repair, may further contribute to improved clinical outcomes.

Importantly, these experiments model early intervention following infection and therefore, do not fully recapitulate the clinical scenario in which patients typically present with established corneal disease. Nevertheless, the observed preservation of host tissue and immune cell viability, in the absence of direct bactericidal effects, supports the concept that ExoU inhibition could complement conventional antibiotics by limiting ongoing tissue damage during infection. Future studies incorporating delayed-treatment paradigms and combination therapy with standard-of-care antibiotics will be important to further define the therapeutic potential and translational applicability of this approach.

## Conclusions

Across biochemical, cellular, *ex vivo*, and *in vivo* models, we demonstrate that repurposed FDA-approved agents zinc pyrithione, bismuth subcitrate, and polymyxin B, effectively inhibit the *P. aeruginosa* virulence factor ExoU by distinct but complementary mechanisms. Monotherapies provided partial protection, while dual and triple combinations delivered considerable improvements in enzyme inhibition, cell viability, tissue integrity, and host survival. Importantly, these antivirulence effects occur without reducing bacterial burden or inhibiting T3SS gene expression, highlighting a strategy to neutralise pathogenicity without driving antibiotic resistance. Our work establishes a robust framework for combination anti-ExoU therapies and paves the way for clinical translation to improve outcomes in bacterial keratitis and highlights the potential of antivirulence strategies as complements to existing antibiotics [52].

## Materials and methods

### Ethics statement

Primary human corneal epithelial cells were derived from donor corneas obtained through the Liverpool Research Eye Bank with approval from the Central University Research Ethics Committee under the study “The Eye and its Repair” (Dr Carl Sheridan; IRAS project ID 239185). Written informed consent for research use was obtained from donors.

Porcine eyes were obtained from CS. Morphet & Sons abattoir (Widnes, UK) as by-products of routine commercial slaughter; no pigs were sacrificed specifically for this study. Work with *Galleria mellonella* does not currently require regulatory approval in the UK.

All mouse experiments were approved by the Cleveland Clinic Institutional Biosafety Committee (IBC protocol 1419) and the Cleveland Clinic Institutional Animal Care and Use Committee (IACUC protocol 0000–2324). Animal studies were performed in accordance with Public Health Service (PHS) Policy on Humane Care and Use of Laboratory Animals, National Institutes of Health Office of Laboratory Animal Welfare guidelines, and the Association for Research in Vision and Ophthalmology Statement for the Use of Animals in Ophthalmic and Vision Research. The Cleveland Clinic animal care and use program is accredited by the Association for Assessment and Accreditation of Laboratory Animal Care International (AAALAC International).

### ExoU inhibitors

The full FDA-approved compound library was purchased from Selleckchem, Houston, USA (3,034 compounds, Catalog No.L1300). Follow up hits for downstream analysis were also purchased separately from Selleckchem. Custom synthesised “Poltide” fatty acid peptide analogues were purchased from Biomatik, Kitchener, Ontario, Canada.

### Recombinant protein production

Full-length *exoU* was cloned into pUCP20T to generate a construct encoding ExoU with a C-terminal 6 × His tag. Human phospholipases *PLA2G7* and *PLA2G4C* were cloned into pET28a, each encoding an N-terminal 6 × His tag. All plasmid constructs were verified by Sanger sequencing (Eurofins Genomics, Ebersberg, Germany). For protein expression, transformed C43(DE3) *E. coli* were cultured in 8L Terrific broth (Melford Laboratories Ltd, Ipswich, UK) supplemented with ampicillin (for pUCP20T) (100 μg/mL) or kanamycin (for pET41a) (50 μg/mL) and grown to an optical density (OD_600_) of 0.8 before induction of recombinant protein expression using 0.4 mM isopropyl-β-d-thiogalactopyranoside (IPTG). ExoU was expressed for 3 hours at 30°C [25] and Lipoprotein-associated phospholipase A_2_ (PLA2G7) and cytosolic PLA_2_γ, (PLA2G4C) were expressed overnight at 18 ˚C. *E. coli* were isolated by centrifugation (5,000 g, 10 minutes) and lysed by sonication in 20 mM Tris-HCl pH 8.2, 300 mM NaCl, 0.1% (v/v) Triton-X-100, 10 mM imidazole, 10% (v/v) glycerol and a complete protease inhibitor cocktail tablet (Roche, Welwyn Garden City, UK). Proteins in the cleared lysate were initially purified immobilised nickel affinity chromatography, followed by size-exclusion chromatography (SEC) (16/600 Superdex 200, GE Healthcare, Amersham, UK) in 20 mM Tris-HCl pH 8.2, 100 mM NaCl and 10% (v/v) glycerol. Recombinant proteins were frozen in liquid nitrogen and stored at −80°C.

### Phospholipase assays

#### High throughput screening.

Screens were performed in 384-well, flatbottom Corning plates (Corning, Sunderland, UK) in a final volume of 20 µL per well. The reaction buffer was 20 mM -bottom Corning plates (Corning, Sunderland, UK) in a final volume of 20 µL per well. The reaction buffer was 20 mM TrisHCl-HCl (pH 8.2) and 100 mM NaCl. Each well contained: 175 nM recombinant His₆-ExoU, 5 µM bovine ubiquitin (Sigma-Aldrich, Gillingham, UK), 1 µM PIP_2_ (Avanti Polar Lipids, Alabaster, AL, USA), 0.6 mM arachidonoyl thiophosphatidylcholine-phosphatidylcholine (Cambridge Bioscience Ltd., Cambridge, UK), 1 mM DTNB (5,5′-dithiobis-(2-nitrobenzoic acid), Sigma) with DMSO at 0.5% (v/v).

Using an Echo 555 acoustic dispenser, 100 nL of each 10 mM compound stock from the full FDAapproved library (Selleck Chemicals, Houston, TX, USA) was added to assay plates to achieve a final concentration of 50 µM. Reactions were initiated by adding ExoU enzyme, incubated at 25 °C, and absorbance at 414 nm was measured after 1 h using a Hidex plate reader. Percent inhibition was calculated relative to DMSO controls.-approved library (Selleck Chemicals, Houston, TX, USA) was added to assay plates to achieve a final concentration of 50 µM. Reactions were initiated by adding

### IC₅₀ Determination

Lead compounds were assayed in a 9point serial dilution series in triplicate. Realtime absorbance readings at 414 nm were recorded every minute for 60 min. For each well, the initial linear portion of the A_414_ vs. time curve (5–15 min) was fitted by least squares regression to yield a slope (ΔA_414_/min). Product formation rates (µmol/min) were calculated using point serial dilution series in triplicate. Real-time absorbance readings at 414 nm were recorded every minute for 60 min. For each well, the initial linear portion of the A-squares regression to yield a slope (ΔA


$$\begin{array}{c} v(\mu mol/min)=\frac{\Delta A\text{4}\text{1}\text{4}/\Delta t}{\varepsilon \times path\;length}\times Vwell \end{array}$$

Rates were then normalized to ExoU mass in each well (µg) to give specific activity (µmol/min/µg). Normalized rates were plotted against the logarithm of compound concentration, and IC₅₀ values were determined by fitting a three-parameter nonlinear regression dose-response model in GraphPad Prism.

### Human phospholipase assays

Recombinant human PLA_2_G7 and PLA_2_G4C activities were measured in 96well, -well, UVtransparent microplates (Corning) in a final volume of 50 µL per well. Reactions were carried out in 20 mM -transparent microplates (Corning) in a final volume of 50 µL per well. Reactions were carried out in 20 mM TrisHCl-HCl (pH 8.2), 100 mM NaCl, and 0.01% Tween 20, with a 1% (v/v) final DMSO concentration. For PLA_2_G7, substrate cleavage was monitored using 0.6 mM 2-thio-PAF (Cambridge Bioscience Ltd), and for PLA_2_G4C, 0.6 mM arachidonoyl thio-bis-glycerophosphocholine (thioBGC-BGC; Cambridge Bioscience Ltd) was employed. In both assays, 1 mM DTNB (5,5′-dithiobis-(2nitrobenzoic acid), -nitrobenzoic acid), SigmaAldrich-Aldrich) served as the chromogenic reporter. Enzyme (500 nM final) was preequilibrated with buffer at 25 °C for 5 min before initiating-equilibrated with buffer at 25 °C for 5 min before initiating the reaction by substrate addition. Absorbance at 414 nm was recorded every minute for 1 h on a Hidex plate reader.

### Secondary Incucyte S3 screen to assess compound efficacy in a wound infection assay

Human corneal epithelial (HCE-T) cells (donated by Kaoru Araki-Sasaki, Japan) were seeded into ImageLock 96-well plates (Sartorius, Epsom, UK) and cultured until confluence in Dulbecco’s modified Eagle’s medium (DMEM)/F-12 medium (Gibco, Thermo Fisher Scientific, Loughborough, UK) with 10% (v/v) fetal bovine serum (FBS; Gibco). Uniform scratch wounds were generated using the IncuCyte WoundMaker (Sartorius) and plates were washed twice with warm phosphate buffered saline (PBS) to remove detached cells.

Cells were infected with *P. aeruginosa* strain PA103 (multiplicity of infection (MOI) 10). In some conditions, moxifloxacin (2.5 µM; 50% minimal inhibitory concentration (MIC)) was added to restrict bacterial overgrowth and extend the assay window [25,30]. Test compounds were added simultaneously at the following final concentrations: Bis 5 µM, Zp 1 µM, Pol 1 µM, cyclofenil (Cyclo) 5 µM, mivacurium chloride (MivC) 5 µM, and chlorhexidine (Chlorh) 5 µM. All compounds were diluted in DMSO, and vehicle controls (0.1% DMSO) were included in each plate.

To monitor cell viability and lysis, calcein-AM (1 µM; Invitrogen, Thermo Fisher Scientific, Loughborough, UK) and ethidium homodimer-1 (2 µM; Invitrogen) were added immediately following infection to stain live and membrane-compromised cells respectively. Plates were imaged every 30 minutes for 15 hours using the IncuCyte S3 live-cell imaging system (Sartorius) equipped with a 10x objective. Image acquisition and analysis were performed using IncuCyte S3 software. Quantitative metrics included total live cell area (calcein+) and dead cell count (ethidium+).

Each experimental condition was assayed in duplicate. Post-assay bacterial CFU enumeration was performed in parallel, by serial dilution and agar plating, to confirm that observed cytoprotective effects were not due to antimicrobial activity of the compounds at the concentrations used.

### Nano Differential Scanning Fluorimetry (nanoDSF)

Thermal stability of recombinant His₆-ExoU was assessed using a Prometheus NT.48 instrument (NanoTemper Technologies, Cambridge, UK) without extrinsic dyes, as these were not compatible with the use of PIP_2_. ExoU was diluted to 5 µM in 20 mM TrisHCl-HCl (pH 8.2), 100 mM NaCl, and 10% (v/v) glycerol. To evaluate ligand effects, four samples were prepared: ExoU alone, ExoU with 5 µM PIP_2_, ExoU with 10 µM inhibitor, and ExoU with both 5 µM PIP_2_ and 10 µM indicated inhibitor, each maintaining 1% (v/v) DMSO. Ten microliters of each mixture were loaded into highsensitivity-sensitivity nanoDSF glass capillaries and subjected to a thermal ramp from 25 °C to 95 °C at 1 °C/min. Intrinsic tryptophan fluorescence emission at 330 nm and 350 nm was recorded continuously, and the ratio F₃₅₀/F₃₃₀ was plotted against temperature. Melting temperatures (Tₘ) were determined from the inflection point of the first derivative of the fluorescence ratio using the manufacturer’s analysis software. All conditions were measured in triplicate. Changes in melting temperature (Δ Tₘ) compared to DMSO, or DMSO PIP_2_ controls were plotted using GraphPad.

### BS^3^ crosslinking and SDS-PAGE analysis of ExoU oligomerization

To assess the impact of PIP_2_ and small molecule inhibitors on ExoU oligomerization, recombinant His₆ ExoU (5 µM) was first buffer‐exchanged into 50 mM HEPES pH 7.4, 100 mM NaCl, 10% (v/v) glycerol using desalting spin columns to remove Tris. Four reaction mixtures (50 µL each) were prepared at room temperature: ExoU alone; ExoU with 5 µM PIP_2_; ExoU with 10 µM inhibitor; and ExoU with both 5 µM PIP_2_ and 10 µM inhibitor for 30 minutes at 25 ºC. Bis(sulfosuccinimidyl) suberate (BS^3^) was added fresh to 1 mM and the samples were incubated for 1 h at room temperature. Reactions were quenched by addition of Tris HCl pH 7.4 to 50 mM and a further 15 min incubation at room temperature. Each sample was then mixed 1:1 with SDS PAGE loading buffer and resolved on 8% (w/v) Bis Tris SDS-PAGE gels at 200 V. Proteins were transferred to nitrocellulose membranes in Tris-glycine-methanol transfer buffer at 100 V for 1 h at 4 °C. Membranes were blocked in TBST containing 5% (w/v) non-fat milk for 1 h, then incubated overnight at 4 °C with mouse anti-His monoclonal antibody (Bio Rad; 1:2,000). After washing in TBST, membranes were incubated with HRP-conjugated goat anti-mouse IgG for 1 h at room temperature. Signal was detected by chemiluminescence with X ray film exposure, and band patterns were analysed to determine the distribution of monomeric and oligomeric ExoU species under each condition.

### EGFP-ExoU (S142A) Transfections

HEK293T cells were maintained in Dulbecco’s Modified Eagle Medium (DMEM; Gibco) supplemented with 10% (v/v) FBS, 4 mM L-glutamine, 100 U/mL penicillin and 100 µg/mL streptomycin. The gene encoding the catalytically inactive ExoU S142A was synthesized by GenScript (Piscataway, NJ, USA) and cloned in-frame into the pEGFP-C3 vector using BamHI and NotI restriction sites. Correct insertion and sequence integrity were confirmed by Sanger sequencing (Eurofins Genomics). For analysis of total EGFP-ExoU (S142A) abundance, 2.2 × 10^6^ cells were seeded into 10 cm dishes 24 h prior to transfection. Cells were transfected with 30 µg of pEGFP-C3 vector encoding an N-terminal EGFP-tagged catalytically inactive ExoU (S142A) using polyethylenimine (PEI) (Sigma-Aldrich, Gillingham, UK) at a 3:1 PEI:DNA ratio. After 24 h, cells were lysed and subjected to SDS-PAGE followed by Western blotting using an anti-GFP antibody (Cell Signaling Technology, London, UK) and HRP-conjugated anti-goat secondary antibody. GAPDH was used as a loading control. Protein detection was performed using enhanced chemiluminescence and imaged with a Bio-Rad ChemiDoc imaging system.

### Confocal Microscopy of EGFP-ExoU and RFP-LAMP1 Co-Localization

HEK293T cells were seeded in 4-compartment glass-bottom imaging dishes (Ibidi, Gräfelfing, Germany) and transfected at approximately 70% confluency with constructs encoding EGFP-ExoU (S142A) (pEGFP-C3 backbone) using polyethylenimine (PEI) at a 3:1 PEI:DNA ratio, and RFP-LAMP1 (CellLight Lysosomes-RFP, BacMam 2.0, Thermo Fisher Scientific, Loughborough, UK) by modified baculovirus delivery. Two hours post-transfection, cells were treated with 0.1% (v/v) DMSO or the indicated compounds. Live-cell imaging was performed at 16 h post compound treatment using a Zeiss LSM780 confocal microscope equipped with a 63 × oil immersion objective (NA 1.4), a temperature and CO_2_ controlled incubation chamber (37°C, 5% (v/v) CO_2_), and laser lines at 488 nm and 561 nm for excitation of EGFP and RFP, respectively. Z-stacks were acquired at 0.5 µm intervals with 1024 × 1024 resolution and a pinhole size of 1 Airy unit.

Colocalisation between EGFP-ExoU(S142A) and lysosomes (Lysosome-RFP, BacMam) was quantified in ImageJ (Fiji) using the Coloc2 plugin. Images were background-subtracted using a 1-pixel rolling ball filter, and Pearson’s correlation coefficients were calculated between the green (ExoU) and red (lysosomal) channels. Data were obtained from three independent experiments.

### Inhibitor toxicity analysis in human primary corneal cells

Donor human corneas were obtained from the Liverpool Research Eye Biobank (LREB). Corneal rims were quartered and incubated with 1.2 U/mL Dispase II (Roche) in PBS for 2 h at 37 °C. The limbal region was gently scraped using fine forceps to isolate limbal epithelial cells, which were then resuspended and triturated in corneal epithelial cell medium (CECM; DMEM:F12 supplemented with 10% (v/v) FBS, penicillin-streptomycin (100 µg/mL), epidermal growth factor (10 ng/mL), hydrocortisone (0.4 mg/mL), insulin (5 mg/mL), adenine (0.18 mM), transferrin (5 mg/mL), triiodothyronine (T3, 2 nM), and cholera toxin (0.1 nM; Sigma-Aldrich)). Cells were seeded onto mitotically inactivated 3T3-J2 feeder layers, pretreated with 4 mg/mL mitomycin C for 2 h. Cultures were maintained in CECM, with medium changes three times per week. After 12–14 days of growth, epithelial cells were passaged using 0.5% trypsin-EDTA (Invitrogen) and transferred to 96-well plates for compound cytotoxicity assays.

To assess the cytotoxicity of candidate inhibitors (Bis, Zp, and Pol), lactate dehydrogenase (LDH) release was measured in primary human limbal epithelial cells following 48 hours of compound exposure. Cells were seeded into 96-well plates at a density of 8,000 cells/well in CECM and incubated overnight at 37 °C to allow attachment. After 48 hours of treatment with compounds diluted in CECM, LDH release into the culture supernatant was quantified using the Cytotoxicity Detection Kit (Thermo Fisher) according to the manufacturer’s protocol. Absorbance was measured at 490 nm with a reference wavelength of 620 nm using a Hidex microplate reader. Background signal was subtracted from all wells, and values were normalized to the maximal LDH release control provided in the kit. Each condition was tested in duplicate wells, and the experiment was repeated independently three times (n = 3). IC₅₀ values were calculated by fitting the data to a nonlinear regression model (inhibitor vs. response, variable slope) using GraphPad Prism.

### Tertiary CellDiscoverer 7 high-content microscopy screen of clinical *P. aeruginosa* keratitis isolates

HCE-T cells were seeded at 8,000 cells/well into clearbottom-bottom 96-well plates (Corning). Plates were incubated at 37 °C, 5% (v/v) CO_2_ and monitored until cultures reached ~80% confluence (~48 h). Immediately prior to infection, DMEM F12 was replaced with DMEM F12 containing 1 µM calceinAM-AM and 2 µM ethidium homodimer-1 and incubated for 30 min at 37 °C. A panel of 52 clinical *P. aeruginosa* keratitis isolates (28 *exoU*^+^; 24 *exoS*^+^) was used. Bacteria were grown to midlog-log phase in LB, washed, and resuspended in PBS. Cells were infected at MOI 10 in the presence of Bis 10 µM, Zp 2.5 µM, or Pol 1 µM or indicated combinations thereof. All compounds were prepared in DMSO and added to each well at 0.1% v/v DMSO; vehicle controls received 0.1% DMSO.

Plates were transferred to a Zeiss CellDiscoverer 7 (CD7, Carl Zeiss Microscopy GmbH, Germany) equipped with environmental control (37 °C, 5% (v/v) CO_2_) and imaged at 5 × magnification (0.25 NA). Three-channel images (transmitted light (oblique), Calcein AM and ethidium homodimer1) were captured automatically at 30-minute intervals over 4.5 h (Table 4). A bespoke image analysis pipeline developed by Dr. Marie Held at the University of Liverpool Centre for Cell Imaging (CCI) was used. This automated workflow, implemented in Zen Blue and Python, is freely available via Jupyter Notebook. All raw image data are deposited in the EMBL-EBI BioImage Archive: DOI: 10.6019/S-BIAD2625) [53].

**Table 4:** Imaging parameters for live/dead cell microscopy using the Zeiss CellDiscoverer 7: Detailed imaging settings used for automated time-lapse acquisition of transmitted light, Calcein AM, and ethidium homodimer-1 signals over 8 hours. The table lists excitation and emission filter specifications, objective and numerical aperture, detectors, and light source parameters for each imaging channel.

Microscope component	Calcein	Ethidium	Oblique (Transmitted light)
Light source	LED module 470 nm 10% intensity Excitation λ: 494 nm	LED module 520 nm 80% intensity Excitation λ: 520 nm	LED lamp 0.1% intensity
Excitation Emission optics	Widefield fluorescence Filter Excitation λ: 450–488 Beam Splitter: 394, 510, 673 Filter Emission λ: 412–433, 501–547, 617–758	Widefield fluorescence Filter Excitation λ: 498–533 Beam Splitter λ: 414, 529, 673 Filter Emission λ: 458–474, 546–564, 618–756	Transmitted Light - Oblique Filter Excitation λ: N/A Beam Splitter λ: 414, 529, 673 Filter Emission λ: 458–474, 546–564, 618–756
Objective lens	Plan-Achromat 5x/ 0.3 NA With a 1x tube lens Effective NA: 0.25	Plan-Achromat 5x/ 0.3 NA With a 1x tube lens Effective NA: 0.25	Plan-Achromat 5x/ 0.3 NA With a 1x tube lens Effective NA: 0.25
Detector Zeiss Axiocam 712 mono 1x camera adaptor	Exposure time: 80 ms Depth of focus: 8.39 µm Binning: 1, 1 Bit depth: 14 bit Scaling: 0.691 µm × 0.691 µm	Exposure time: 80 ms Depth of focus: 8.39 µm Binning: 1, 1 Bit depth: 14 bit Scaling: 0.691 µm × 0.691 µm	Exposure time: 3.5 ms Depth of focus: 8.98 µm Binning: 1, 1 Bit depth: 14 bit Scaling: 0.691 µm × 0.691 µm

Images were pre-processed and analysed in Zen Blue (3.7.5, Zeiss, Germany) using an automated macro (Github link to Zen macro). The image was split into the fluorescence channels and the oblique channel. The fluorescence channels were processed via background subtraction (radius = 30) and then merged again with the raw oblique channel. Segmentation was performed in Zen Blue (Github link to analysis settings) using the built-in multichannel segmentation algorithm with background subtraction and thresholding (Otsu method) applied to identify regions of interest (ROIs). Objects were filtered based on size, intensity, and shape metrics to ensure accurate quantification. A minimum object area of 50 µm^2^ and a maximum of 1000 µm^2^ were applied to exclude small artifacts and excessively large structures. Intensity-based filtering was performed on Calcein and EtHD1 fluorescence channels, where objects with mean intensity below 500 (Calcein) or above 2000 (EtHD1) were excluded to remove low-signal noise and overexposed regions. Additional morphological constraints were applied, including convexity, circularity, and compactness thresholds, to refine object selection and remove irregularly shaped debris. Objects overlapping by more than 60% were separated using a watershed-based approach to prevent merging of distinct structures. The final dataset included extracted features such as object count, mean area, relative image area coverage, and integrated density. Image processing and measurements were conducted uniformly across all images the Zen macro which calls the Zen analysis pipeline automatically.

Downstream processing of the measured features, e.g., population average and live/dead ratio calculations were performed in batch in Python, segmented live (calcein⁺) and dead (ethidium⁺) cells and computed total livecell area (µm^2^) for assessment of ExoU mediated cell lysis. Cell convexity measurements were performed to assess the impact of ExoS mediated cell rounding. Each condition was assayed in duplicate with data plotted as mean ± SD of livecell area (µm^2^) at 5 h. Figure generation was performed in Python and GraphPad Prism.

### Quantitative RT-PCR analysis of T3SS gene expression

*P. aeruginosa* strains PA103 and PAO1 were grown overnight in LB at 37 °C with shaking in the presence of Bis 10 µM, Zp 2.5 µM, or Pol 1 µM. EGTA (2.5 mM) was included as a positive control to induce T3SS expression.

Total RNA was extracted from cultures using the RNeasy Mini Kit (Qiagen) according to the manufacturer’s instructions, followed by DNase I treatment to remove residual genomic DNA. First-strand cDNA synthesis was performed using a Reverse Transcription kit (Promega). Quantitative PCR was carried out using SYBR Green Master Mix (Bio-Rad) on an Applied Biosystems StepOnePlus Real-Time PCR instrument.

As previously described, predesigned gene-specific primers were used to amplify *exsA*, *pcrV*, and *exoU* in PA103, and *exsA*, *pcrV*, and *exoS* in PAO1 [25,30]. RNA polymerase β-subunit gene (rpoB) was used as the internal reference for normalisation. Relative gene expression levels were calculated using the ΔΔCt method, and fold changes were expressed relative to vehicle-treated controls. All reactions were performed in technical triplicates from three independent cultures.

### *Ex vivo* porcine corneal infection studies

Porcine corneas were processed and infected as previously described [38]. Briefly, porcine eyes were obtained from a local abattoir (CS Morphet & Sons, Widnes, UK) within 6 h of slaughter and transported on ice. All procedures were conducted under sterile conditions, as described [38]. Corneas with a 2–3 mm scleral rim were excised, immersed in 10% (v/v) iodinated povidone for 2 min, then rinsed twice in PBS and blotted dry. A single flange of a sterile plumbing ring (B&Q, UK: inner diameter 10 mm) was coated with LIQUIBAND topical skin adhesive and pressed centrally onto the epithelial surface.

To support the stroma and endothelium, 0.5% (w/v) UltraPure agarose in DMEM was prepared by dissolving at 65.5°C, cooling to ~37°C, and pipetting 1 mL onto the endothelial side. Once solidified, each cornea was transferred, epithelial side up, into a 12‑well plate. A defined epithelial wound was generated by applying 10 µL 70% ethanol (v/v in PBS) into the ring (corneal apex) for 10 s, then gently debriding the treated area with a scalpel blade. Corneas were rinsed three times in PBS to remove debris and residual ethanol, and wells were filled with 1 mL DMEM, ensuring the epithelium remained hydrated without submerging the ring.

PA103 was cultured to an OD_600_ of 0.8 in LB, washed twice in PBS, and adjusted to 2 × 10⁶ CFU/mL. A 50 µL bacterial inoculum (1 × 10⁵ CFU) was combined with inhibitors Bis 5 µM, Zp 2.5 µM, Pol 1 µM, alone or in combination, or 20 µM moxifloxacin (positive control), all in 0.1% DMSO. Each treatment (or vehicle) was added into the ring, and corneas were incubated at 37°C and 5% CO_2_ for 48 h.

Corneal opacity was measured by scanning whole mounts at 400 nm on a Hidex microplate reader and expressed as optical density (OD_4_₀₀). Epithelial ulceration was quantified by photographing corneas on a Bio‑Rad ChemiDoc system and tracing wound areas in ImageJ.

For bacterial burden, rings and adhesive were removed, corneas rinsed in PBS, homogenized in 1 mL PBS using a tissue disruptor, and serial dilutions plated on LB agar. Plates were incubated overnight at 37 °C, and CFU per cornea were determined.

For histology, corneas were fixed in 10% (v/v) neutral‑buffered formalin for 24 h, dehydrated, and embedded in paraffin. Five‑micron sections were stained with hematoxylin and eosin and scanned on a VENTANA DP 200 slide scanner (Roche).

All conditions were tested in triplicate using corneas from at least three different animals. Data are presented as mean ± SD. Statistical analyses were performed by one‑way ANOVA with Tukey’s post hoc test.

### *Galleria mellonella* infection studies

To assess the therapeutic potential of ExoU inhibitors *in vivo*, *Galleria mellonella* larvae (Pets at Home, UK) weighing 250–300 mg were used. Larvae were stored at 15 °C in the dark and acclimated to room temperature prior to experimentation. Infections were carried out using PA103, grown to mid-log phase in LB, washed twice in PBS, and adjusted to 1 × 10⁷ CFU/mL. Using a 10 μL Hamilton syringe (Hamilton 702 N, 30 gauge), larvae were injected with 10 μL of bacterial suspension (1 × 10⁵ CFU) into the last left proleg. Control larvae were injected with 10 μL PBS.

Immediately following infection, larvae were treated topically at the injection site with individual or combined ExoU inhibitors: Bis 0.1 mg/g, Zp 0.01 mg/g, or Pol 0.004 mg/g, alone or in combination. Inhibitors were prepared in PBS containing 0.1% (v/v) DMSO, and 10 μL of each treatment was applied using a micropipette directly to the injection site. DMSO-only vehicle controls were included.

Larvae were incubated in the dark and monitored at 8 h intervals over 40 h. Health was assessed every 8 hours using a validated scoring system [39] which evaluates activity, melanisation, responsiveness to touch, and cocoon formation (maximum score = 10). Since the infection period in our assays was limited to 40 h, no cocoon formation was observed in either infected or control larvae. Consequently, cocoon formation (1 point) was excluded from the scoring, and the maximum achievable score in our experiments was 9. In parallel, survival was recorded for Kaplan-Meier survival analysis [54].

Clinical score data were analysed using a linear mixed-effects model with repeated measures and Tukey’s post hoc test for multiple comparisons. Survival differences were evaluated using the Kaplan-Meier method with log-rank testing.

### *In vivo* mouse keratitis studies

A previously established scratch infection model was employed in C57BL/6J mice [40]. Female mice (10–12 weeks old; Jackson Laboratory) were anesthetised via intraperitoneal injection (50 μL/25g body weight) of ketamine (50 mg/kg body weight) and dexmedetomidine (0.375 mg/kg body weight), followed by atipamezole (3.75 mg/kg body weight) for reversal. Ethiqa XR (extended-release buprenorphine) was administered to alleviate potential discomfort associated with infection.

A clinical *P. aeruginosa* keratitis isolate strain, PA48386, was cultured in LB broth overnight and then subcultured and grown to log phase (OD_600_ 0.8). PA48386 cells were collected by centrifugation (5,000 g for 5 min) and resuspended in PBS to yield 2 × 10⁶ CFU/mL suspension. On anesthetised C57BL/6J mice corneal infections were induced by creating three parallel epithelial scratches in the central cornea of one eye only using a 25-gauge needle, followed by topical application of 5 μL PA48386 in PBS (inoculum containing 1 × 10⁴ CFU). After 30 minutes, mice were treated topically with 5 μL of individual ExoU inhibitors or combinations: Bis 50 μM, Zp 20 μM, Pol 1 μM, or vehicle control (PBS with 0.1% v/v DMSO). Treatments were repeated at 4 h and 24 h post-infection.

Corneal images were acquired at 24 h and 48 h post-infection using a stereomicroscope (Amscope SM-2TZ) equipped with a 10 MP Aptina MT9J003 CMOS digital camera under anaesthesia, after which animals were euthanised for downstream analyses. A total of 564 images were collected across both timepoints.

Corneal disease severity was scored independently by two masked observers on two separate occasions using a previously described extended 16-point grading system [40,41,55]. This composite system evaluates four parameters: (i) area of opacity, (ii) density of central opacity, (iii) density of peripheral opacity, and (iv) epithelial surface integrity. Individual parameter scores were summed to generate a total score ranging from 0 (no infection) to 16 (severe infection).

Inter- and intra-observer reliability were assessed using Cohen’s weighted kappa (SPSS v25), demonstrating high reproducibility (intra-rater κ = 0.77–0.84; inter-rater κ = 0.74–0.78).

Statistical analysis was performed using a general linear model (GLM), with clinical score at 24 h (Table 2) and 48 h (Table 3) as dependent variables and treatment group as the independent factor. Post hoc comparisons were performed using Tukey’s multiple comparison test, with significance defined as p < 0.05.

### Flow cytometry

Mouse corneas were processed for flow cytometry analysis and CFU enumeration as previously described [41]. Mouse corneas were dissected and digested in collagenase type I (82 U/cornea in 150 μL PBS; Millipore Sigma) at 37 °C for 2 hours. A portion of the digest (5 μL) was serially diluted and plated on LB agar for CFU enumeration. The remaining suspension was processed for flow cytometry to assess immune cell infiltration and viability. Cells were washed and incubated on ice for 10 min in 100 μl ice cold FACS buffer (PBS with 1% FBS) containing 2 μg Fc blocker (anti-mouse CD16/CD32 antibody clone 93; Biolegend). Cell surface staining was conducted on ice for 1 h with APC/Cy7 anti-mouse CD45 (clone 30-F11; Biolegend), PE anti-mouse neutrophil Ly6B.2 (clone 7/4; Abcam), PE/Cy5 anti-mouse F4/80 (clone BM8; Biolegend) antibodies. All samples were also stained with Zombie Violet fixable dye (Biolegend) for dead cells. Stained cells were washed twice with FACS buffer and resuspended in 1% PFA, then detected by BD LSRFortessa flow cytometer. Cells were gated by forward and side scatter, viability, CD45^+^ population, and Ly6B.2^+^ and F4/80^+^ subpopulations. Unstained samples and fluorescence minus one (FMO) control were used to set boundaries for background and positive populations. Analysis of flow cytometric data was performed using BD FlowJo software (v.10). Neutrophils were defined as CD45^+^Ly6B.2^+^F4/80^−^, and non-immune cells as CD45^−^.

### Statistics

Results were obtained from at least three independent experiments unless otherwise stated and are presented as mean ± standard deviation (SD). Statistical analyses were performed using GraphPad Prism and SPSS (version 31). Where appropriate, one-way ANOVA was used to compare multiple groups, followed by Tukey’s post hoc test with correction for multiple comparisons. Significance was defined as * < 0.05, ** < 0.01, *** < 0.001, **** < 0.0001.

## Supporting information

S1 Table Primary high-throughput screening data for identification of ExoU inhibitors from the Selleckchem FDA-approved drug library. A high-throughput phospholipase assay using recombinant ExoU (175 nM; specific activity 5.85 ± 0.26 μmol/min/μg) was used to screen 3,042 FDA-approved compounds at 50 µM for inhibition of ExoU activity. Phospholipase activity was quantified by absorbance at 414 nm following a 1 h enzymatic reaction. Percentage ExoU activity for inhibitor conditions was calculated with reference to 0.1% (v/v) DMSO controls. The table includes the complete screening dataset comprising initial hits, compounds excluded due to interference with assay readouts, and dose-response validated compounds.
(XLSX)

S1 Fig Purification of and analysis of ExoU phospholipase activity: His-tagged ExoU was purified from C43(DE3) *E. coli* by Immobilised metal affinity chromatography (IMAC) followed by (A) size exclusion chromatography prior to (B) SDS-PAGE analysis. (**C**) ExoU hydrolysis of arachidonoyl Thio-PC substrate was analysed in the presence of activating co-factors PIP_2_ (0.5 µM) and mono-ubiquitin (10 µM).
(TIF)

S2 Fig Dose-response analysis to determine ExoU inhibitor Ki values. Phospholipase assays were performed with varying concentrations of identified inhibitors. Inhibition at each inhibitor concentration was plotted against log[inhibitor] and fitted to a four-parameter logistic model to obtain IC₅₀ values. Ki values were calculated using the Cheng–Prusoff equation, incorporating the independently determined Km for ExoU and the substrate concentration used in the assay. Each data point represents the mean ± SD of three independent experiments, each performed in triplicate. Curve fitting and Ki calculations were performed in GraphPad Prism 9.0.
(TIF)

S3 Fig ExoU inhibitors are not antimicrobial at concentrations used in cellular assays: (A) Dose-response growth curves of *P. aeruginosa* PA103 in LB broth treated with candidate inhibitors, measured by endpoint CFU enumeration after overnight incubation. (B) Endpoint CFU enumeration of *P. aeruginosa* PA103 cultures collected following IncuCyte S3 automated time-lapse microscopy analysis to monitor bacterial growth dynamics in with and without 2.5 µM moxifloxacin. Statistical analysis was performed using two-way ANOVA followed by multiple comparisons against DMSO-treated controls within each condition. While moxifloxacin significantly reduced bacterial counts, no significant differences were observed between inhibitor-treated and DMSO-treated samples in the presence of moxifloxacin. (C) Dose-response growth inhibition of PA103 and a panel of clinical keratitis isolates treated with Pol, determined by OD₆₀₀ measurements after overnight incubation in LB broth (top) and DMEM-F12 cell culture medium (bottom). CFU values were log₁₀-transformed prior to analysis. Data are presented as mean ± SD from three independent experiments. *P < 0.05, **P < 0.01, ***P < 0.001, ****P < 0.0001; ns, not significant.
(TIF)

S4 Fig Bis, Zp and Pol do not inhibit human PLA2 enzymes: (A) Activity of human lipoprotein-associated phospholipase A_2_ (PLA2G7) was measured using 2-thio-PAF as a substrate in the presence of 10 µM indicated inhibitor or 2% (v/v) DMSO controls. (B) ExoU activity was assessed using arachidonoyl thio-phosphatidylcholine (thio-PC) in the presence of 10 µM of the indicated compound. (C) Activity of human cytosolic phospholipase A_2_ (PLA2G4C) was measured using arachidonoyl thio-phosphatidylcholine (thio-BGPC) in the presence of Bis, Pol, or Zp (10 µM).
(TIF)

S5 Fig Toxicity analysis of ExoU inhibitors in human primary corneal cells: Lactate dehydrogenase (LDH) release was measured after 48 h of compound exposure to assess host cell compatibility. Cells were seeded at 8,000 cells/well, treated with compounds at the indicated concentrations, and LDH release was quantified using the Cytotoxicity Detection Kit (Roche). Values were background-subtracted, normalized to maximal LDH release controls, and expressed as mean ± SD (n = 3 independent experiments, each in duplicate). Nonlinear regression analysis (variable slope) was used to calculate LD₅₀ values.
(TIF)

S6 Fig ZnSO_4_ does not protect HCE-T cells from ExoU cell lysis during *in vitro* PA103 infection: Fully confluent HCE-T cells were scratched then infected with PA103 (MOI 10) for 4 hours in the presence of either DMSO (0.1% v/v), 2.5 µM ZnSO_4_ or 2.5 µM Zp, followed by LDH assay analysis to detect cell lysis. Percentage lysis was normalized to the maximal LDH release from the kit positive control (CyQUANT; Thermo Fisher).
(TIF)

S7 Fig Nano differential scanning fluorimetry (nanoDSF) analysis of ExoU thermal stability in the presence of compounds. Raw melt curves of recombinant ExoU are shown in the absence (left) and presence (right) of PIP_2_ (5 µM) from three independent (A-C) experiments. Compounds were tested at 10 µM. Intrinsic tryptophan fluorescence emission was continuously monitored at 330 nm and 350 nm as the temperature increased, and the fluorescence ratio (F₃₅₀/F₃₃₀) was plotted against temperature. Melting temperatures (Tₘ) were calculated from the average inflection point of the first derivative of the fluorescence ratio using the manufacturer’s analysis software.
(TIF)

S8 Fig Combination treatment with Bis, ZP, and Pol does not affect *P. aeruginosa* growth. PA103 was cultured in the presence of 10 µM bismuth subcitrate (Bis), 2.5 µM zinc pyrithione (Zp), 1 µM polymyxin B (Pol), 20 µM moxifloxacin (Mox; positive control) or 0.01% (v/v) DMSO vehicle control. PA103 was subcultured to 0.2 OD_600_ followed by 16h incubation at 37 °C in the presence of compounds, cultures were serially diluted, plated onto agar, incubated overnight at 37 °C, and colony-forming units (CFU) were enumerated to assess bacterial viability. Results shown are mean CFU values with standard deviation. Statistical analysis was performed using one-way ANOVA with multiple-comparisons testing relative to the DMSO control with post hoc Tukey correction in GraphPad Prism.
(TIF)

S9 Fig Broad and enhanced efficacy of ExoU inhibitor combinations across clinical *P. aeruginosa* isolates. A high-content microscopy screen using the Cell Discoverer 7 (CD7) platform was performed to assess the efficacy of ExoU inhibitors against a panel of 29 *exoU* ⁺ *P. aeruginosa* clinical keratitis isolates. HCE-T cells were stained with calcein-AM and ethidium homodimer, and live-cell area (µm²) was quantified at 4.5 h post-infection. Bar charts show live-cell area for each isolate under the indicated treatment conditions, expressed as a percentage relative to non-infected controls. Treatment conditions are shown as: (A) Bis, (B) Zp, (C) Pol, (D) Bis + Zp, (E) Bis + Pol, (F) Zp + Pol, and (G) Bis + Zp + Pol. Each bar represents an individual clinical isolate. Compounds were used at the following concentrations: Bis (10 µM), Zp (2.5 µM), and Pol (1 µM).
(TIF)

S10 Fig Quantitative RT-PCR analysis of Type III Secretion System (T3SS) gene expression. Gene expression of key T3SS components was assessed following bacterial growth in LB medium supplemented with bismuth subcitrate (Bis, 10 µM), zinc pyrithione (Zp, 2.5 µM), or polymyxin B (Pol, 1 µM). For *P. aeruginosa* PA103, *exsA* (T3SS master regulator), *pcrV* (translocon component), and *exoU* (effector) were analysed; for PA01, *exsA*, *pcrV*, and *exoS* were examined. Expression levels were normalised to RNA polymerase β-subunit (*rpoB*). EGTA (2.5 mM) treatment served as a positive control to induce T3SS expression.
(TIF)

S1 Video Live-cell confocal imaging of EGFP-ExoU(S142A) localisation in HEK293T cells following inhibitor treatment. HEK293T cells were seeded in 4-compartment glass-bottom dishes (Ibidi, Gräfelfing, Germany) and transfected at ~70% confluence with constructs encoding EGFP-ExoU(S142A) (pEGFP-C3 backbone) using polyethylenimine (PEI) at a 3:1 PEI:DNA ratio. Sixteen hours post-transfection, cells were treated with DMSO (S1 Video) 5 µM Bis (S2 Video), Pol (S3 Video) or Zp (S4 Video) and imaged live using a Zeiss LSM780 confocal microscope equipped with a 63× ×  oil immersion objective (NA 1.4) and environmental control (37 °C, 5% CO_2_). EGFP fluorescence was excited at 488 nm and z-stacks were acquired at 0.5 µm intervals.
(MP4)

S2 Video Live-cell confocal imaging of EGFP-ExoU(S142A) localisation in HEK293T cells following inhibitor treatment. HEK293T cells were seeded in 4-compartment glass-bottom dishes (Ibidi, Gräfelfing, Germany) and transfected at ~70% confluence with constructs encoding EGFP-ExoU(S142A) (pEGFP-C3 backbone) using polyethylenimine (PEI) at a 3:1 PEI:DNA ratio. Sixteen hours post-transfection, cells were treated with DMSO (S1 Video) 5 µM Bis (S2 Video), Pol (S3 Video) or Zp (S4 Video) and imaged live using a Zeiss LSM780 confocal microscope equipped with a 63 × oil immersion objective (NA 1.4) and environmental control (37 °C, 5% CO_2_). EGFP fluorescence was excited at 488 nm and z-stacks were acquired at 0.5 µm intervals.
(MP4)

S3 Video Live-cell confocal imaging of EGFP-ExoU(S142A) localisation in HEK293T cells following inhibitor treatment. HEK293T cells were seeded in 4-compartment glass-bottom dishes (Ibidi, Gräfelfing, Germany) and transfected at ~70% confluence with constructs encoding EGFP-ExoU(S142A) (pEGFP-C3 backbone) using polyethylenimine (PEI) at a 3:1 PEI:DNA ratio. Sixteen hours post-transfection, cells were treated with DMSO (S1 Video) 5 µM Bis (S2 Video), Pol (S3 Video) or Zp (S4 Video) and imaged live using a Zeiss LSM780 confocal microscope equipped with a 63 × oil immersion objective (NA 1.4) and environmental control (37 °C, 5% CO_2_). EGFP fluorescence was excited at 488 nm and z-stacks were acquired at 0.5 µm intervals.
(MP4)

S4 Video Live-cell confocal imaging of EGFP-ExoU(S142A) localisation in HEK293T cells following inhibitor treatment. HEK293T cells were seeded in 4-compartment glass-bottom dishes (Ibidi, Gräfelfing, Germany) and transfected at ~70% confluence with constructs encoding EGFP-ExoU(S142A) (pEGFP-C3 backbone) using polyethylenimine (PEI) at a 3:1 PEI:DNA ratio. Sixteen hours post-transfection, cells were treated with DMSO (S1 Video) 5 µM Bis (S2 Video), Pol (S3 Video) or Zp (S4 Video) and imaged live using a Zeiss LSM780 confocal microscope equipped with a 63 × oil immersion objective (NA 1.4) and environmental control (37 °C, 5% CO_2_). EGFP fluorescence was excited at 488 nm and z-stacks were acquired at 0.5 µm intervals.
(MP4)

S5 Video Time-lapse imaging of ExoU- and ExoS-mediated cytotoxicity in HCE-T cells infected with clinical *P. aeruginosa* isolates. HCE-T cells were seeded in 96-well plates, stained with Calcein-AM (live-cell marker) and ethidium homodimer (dead-cell marker), and infected at ~80% confluence with *P. aeruginosa* (MOI = 10). Time-lapse imaging was performed every 0.5 h over 4.5 h using a Zeiss CellDiscoverer 7 automated microscope. S5 Video: Infection with *ExoU⁺* clinical isolate 48386, showing progressive Calcein-AM signal loss and membrane rupture consistent with ExoU-mediated cytolysis. S6 Video: Infection with *ExoS⁺* clinical isolate 76026, showing characteristic cell rounding and retraction without loss of Calcein-AM fluorescence, consistent with ExoS-driven cytoskeletal disruption but preserved membrane integrity.
(MP4)

S6 Video Time-lapse imaging of ExoU- and ExoS-mediated cytotoxicity in HCE-T cells infected with clinical *P. aeruginosa* isolates. HCE-T cells were seeded in 96-well plates, stained with Calcein-AM (live-cell marker) and ethidium homodimer (dead-cell marker), and infected at ~80% confluence with *P. aeruginosa* (MOI = 10). Time-lapse imaging was performed every 0.5 h over 4.5 h using a Zeiss CellDiscoverer 7 automated microscope. S5 Video: Infection with *ExoU⁺* clinical isolate 48386, showing progressive Calcein-AM signal loss and membrane rupture consistent with ExoU-mediated cytolysis. S6 Video: Infection with *ExoS⁺* clinical isolate 76026, showing characteristic cell rounding and retraction without loss of Calcein-AM fluorescence, consistent with ExoS-driven cytoskeletal disruption but preserved membrane integrity.
(MP4)

S1 Raw Gel 1: Raw western blot images underlying data in Fig 4A. HEK293T cells were transfected with pEGFP-C3, encoding S142A ExoU with an N-terminal EGFP tag, in the presence of bismuth subcitrate (Bis), polymyxin B (Pol) or zinc pyrithione (Zp) for 16 hours. S1 Raw Images 2: Raw western blot underlying data in Fig 3B. BS^3^ cross-linking assay of recombinant ExoU followed by SDS-PAGE and anti-6 × His immunoblotting, showing compound-dependent changes in oligomerisation in the presence or absence of PIP_2_. S1 Raw Images 3: Raw Coomassie stained SDS-PAGE gel from S1B Fig. Purification of recombinant His-tagged ExoU from *E. coli* by size exclusion chromatography.
(PDF)
