# Supplementary material for: Topical formulation of repurposed FDA-approved compounds inhibits Pseudomonas aeruginosa ExoU and improves corneal infection outcomes
Source: PLoS Pathog. 2026 Aug 3;22(8):e1014474. doi: 10.1371/journal.ppat.1014474 (PMC13450841; doi:10.1371/journal.ppat.1014474)
Supplement: Supplementary file 1 [file ppat.1014474.s018.pdf]

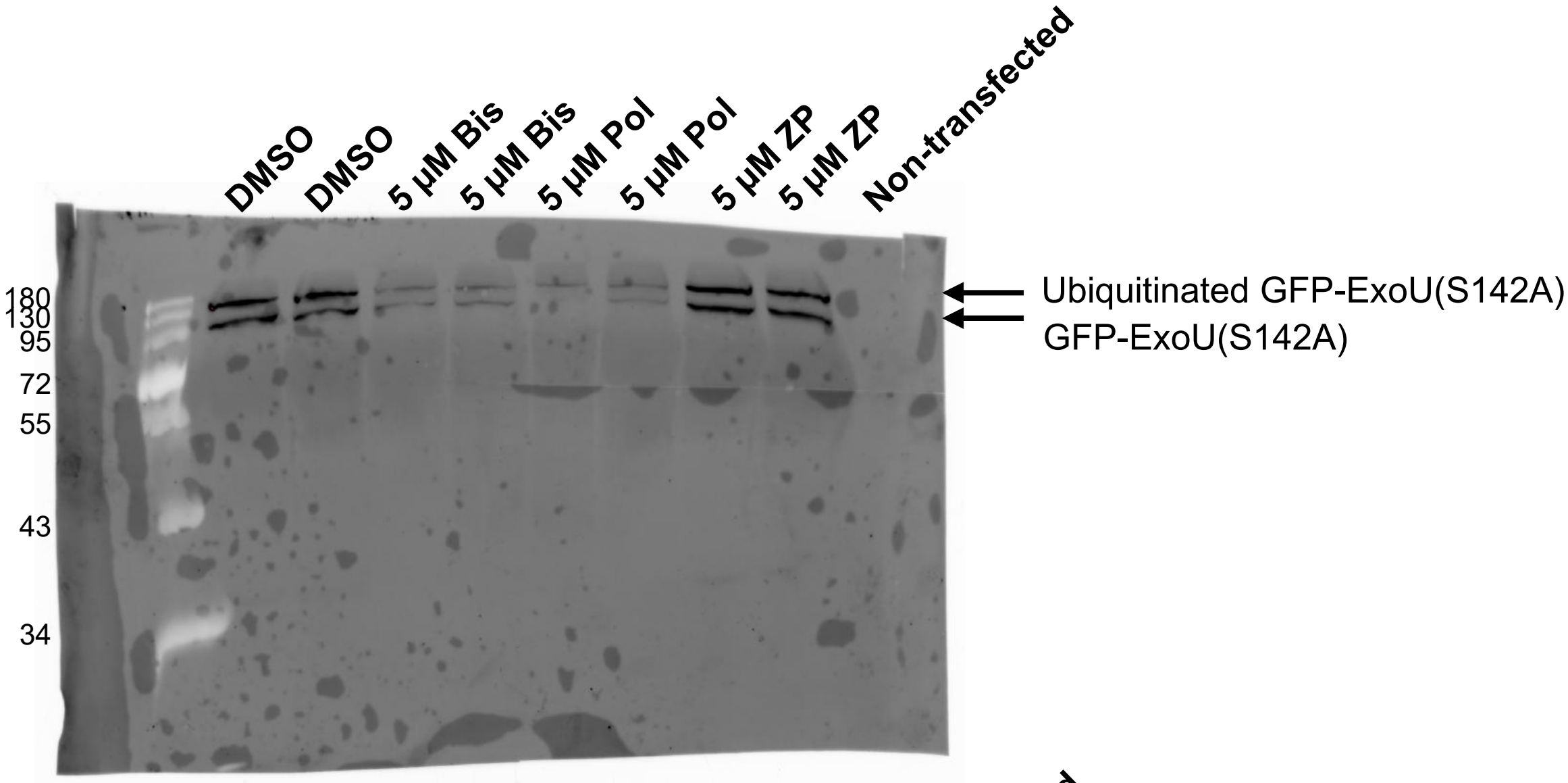

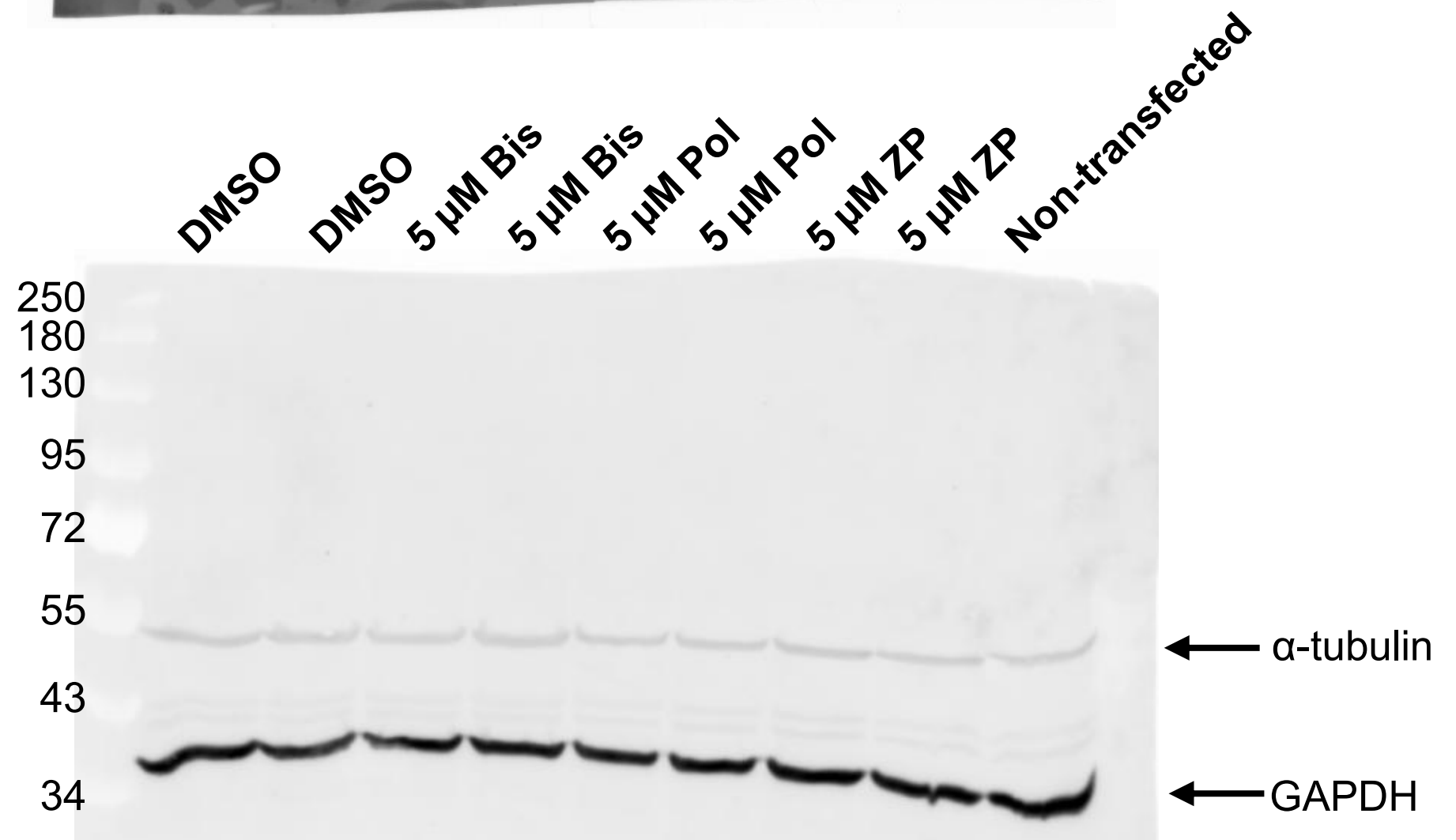

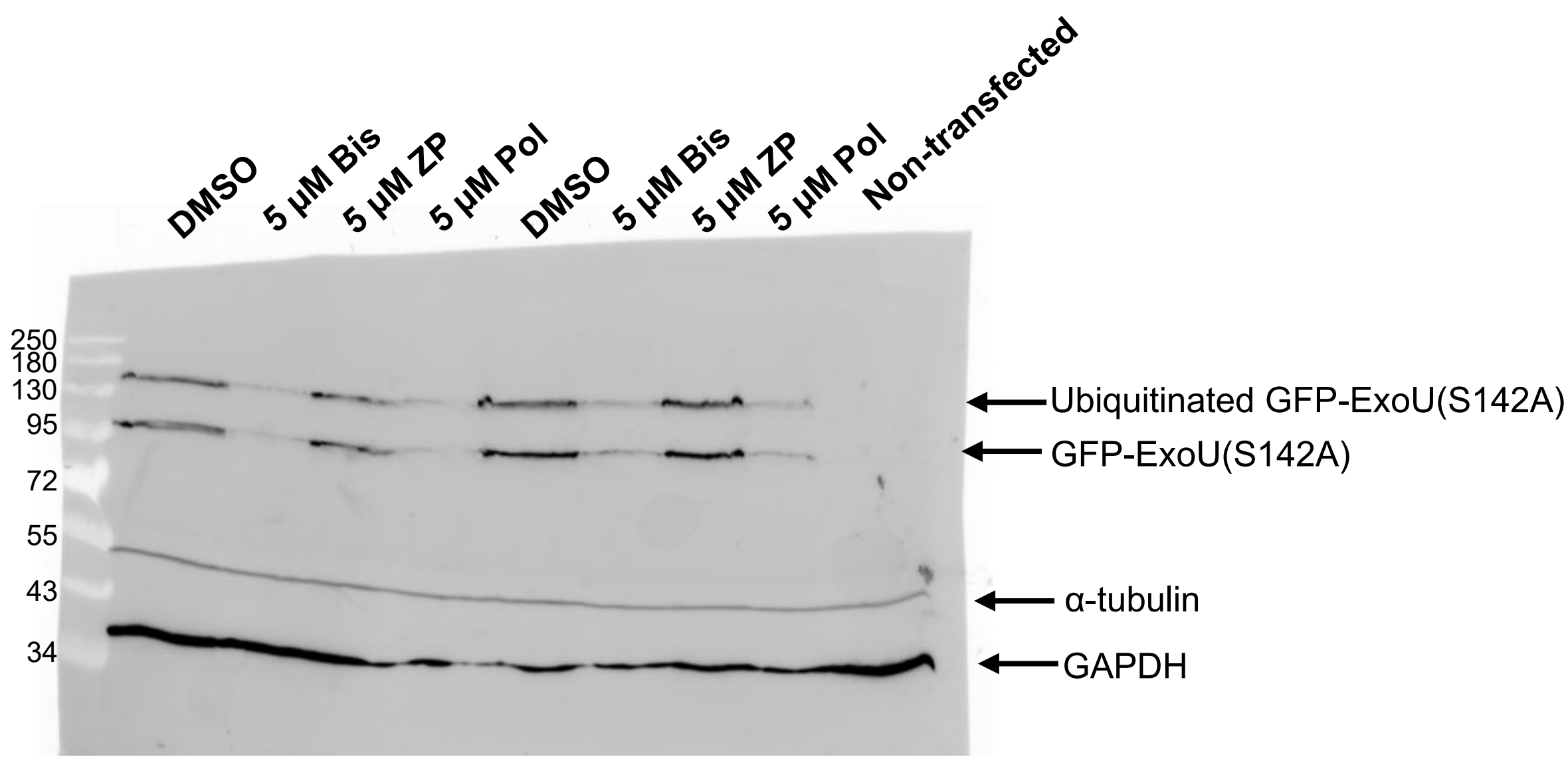

S1 Raw Images 1: Raw western blot images underlying data in Figure 4A. HEK293T cells were transfected with pEGFP-C3, encoding S142A ExoU with an N-terminal EGFP tag, in the presence of bismuth subcitrate (Bis), polymyxin B (Pol) or zinc pyrithione (Zp) for 16 hours.

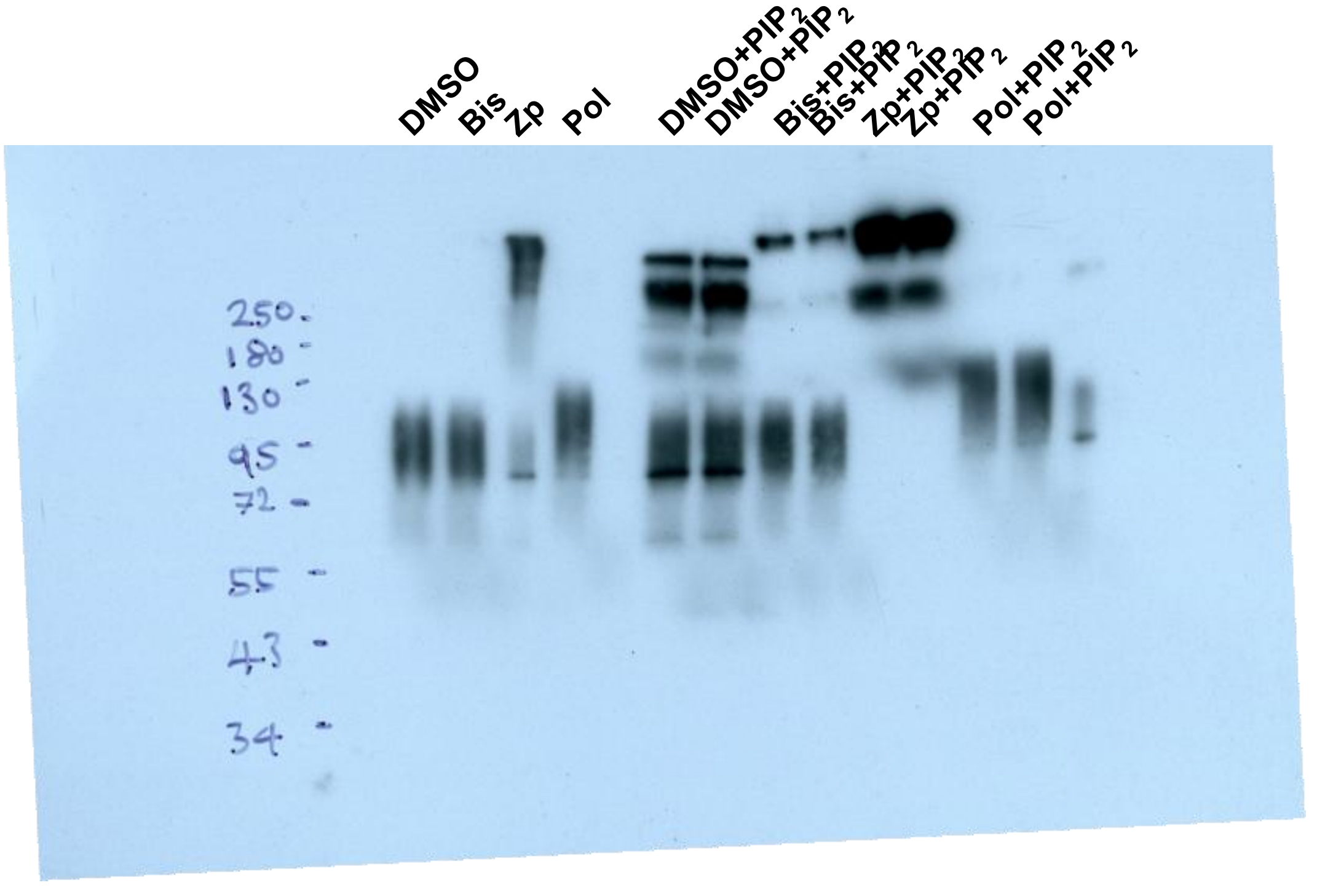

S1 Raw Images 2: Raw western blot underlying data in Figure 3B. BS³ cross-linking assay of recombinant ExoU followed by SDS-PAGE and anti-6×His immunoblotting, showing compound-dependent changes in oligomerisation in the presence or absence of PIP_2.

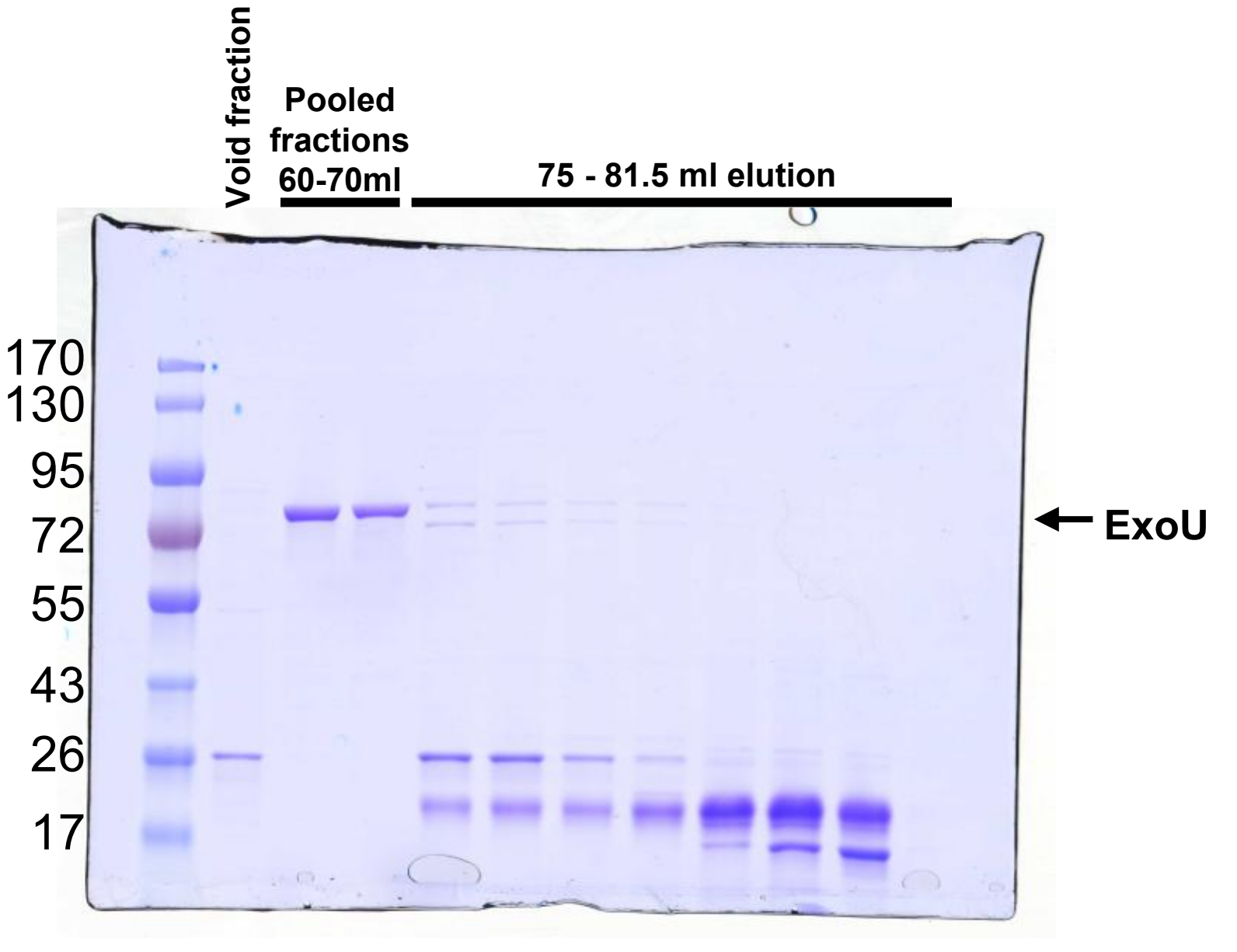

S1 Raw Images 3: Raw Coomassie stained SDS-PAGE gel from Supplementary Figure 1B. Purification of recombinant His-tagged ExoU from *E. coli* by size exclusion chromatography.
