# Supplementary material for: Topical formulation of repurposed FDA-approved compounds inhibits Pseudomonas aeruginosa ExoU and improves corneal infection outcomes
Source: PLoS Pathog. 2026 Aug 3;22(8):e1014474. doi: 10.1371/journal.ppat.1014474 (PMC13450841; doi:10.1371/journal.ppat.1014474)
Supplement: Supplementary file 2 [file ppat.1014474.s019.docx]

Dear Drs. Bernal, Guillard, Bhaduri-McIntosh and Malim,

Thank you for your careful assessment of our manuscript and for the constructive and encouraging feedback. We have undertaken substantial revisions, including additional experimental work, to address the concerns raised. These changes have significantly strengthened the manuscript, improving both its clarity and its translational framing.

In response to the comments from Reviewers #2 and #3 regarding the density and readability of the manuscript, we have extensively revised the text throughout. The Introduction has been streamlined, particularly paragraphs 3-5, to improve focus and accessibility. An overview paragraph has been added at the start of the Results section to guide the reader, with clearer subheadings and internal signposting used to improve structure and narrative flow. We have also reorganised the presentation of data to enhance the logical progression of the study. For example, selectivity data (now Supplementary Figure 4) are presented prior to cytotoxicity data (Supplementary Figure 5).

The high-content analysis of clinical isolates has been substantially revised and expanded. This now includes additional treatment combinations (Zp+Pol and the triple combination Bis+Zp+Pol), with results streamlined for clarity in the main figures (Figure 5A-E). Summary metrics across isolates are now presented in the main text, while detailed isolate-by-isolate responses have been moved to the Supplementary Information. The Discussion and Conclusion have also been refined and shortened to improve focus and reduce redundancy.

## **Response to Editor’s summary**

*First, there is a significant need to improve the translational relevance of the work; reviewers noted that the study currently lacks evidence for the efficacy of these inhibitors in established infections, particularly when used in combination with standard antibiotics, which is the stated clinical goa*l.

**Response:** We agree that this is an essential step in translation. The acquisition of data on efficacy with antibiotics for translation requires first regulatory preclinical toxicology safety data to be established. Otherwise, the data on efficacy may well be meaningless for translation. Preclinical regulatory safety is beyond the scope of this already substantial manuscript. It is correct that in an ongoing infection there is already tissue damage, but the contention is that the application of the inhibitors would help lessen further tissue damage due to continued release of ExoU. Although we have demonstrated in the *in vitro* scratch assays (Figure 1D), the added effect of the inhibitors when used in combination with subMIC_50_ (2.5 µM) doses of moxifloxacin, modelling delayed treatment *in vivo* introduces substantial complexity, including variability in infection kinetics and ethical considerations associated with prolonged untreated infection. We therefore consider such studies to be more appropriately addressed in future work, particularly in translational or clinical settings where time-to-treatment variability is inherent. To clarify this point, we have revised the Discussion to explicitly frame the current study as establishing a mechanistic and preclinical foundation for future translational studies, including delayed-treatment and combination therapy models.

*Second, the manuscript is considered overly dense and difficult to follow; the text should be reorganised to improve the focus and to clarify the rationale for using five different infection models and provide more direct interpretation of the data rather than leaving it to the reader. Finally, technical and mechanistic discrepancies must be addressed, including inconsistencies in figure labelling, reproducibility issues between different figures, and, more importantly, the lack of biochemical evidence regarding how these compounds interact with the enzyme.*

**Response:** We have substantially reorganised the manuscript to improve clarity, explicitly state the rationale for each experimental model, and provide more direct interpretation of the data throughout the Results section. Redundant methodological detail has been reduced or relocated, and figure legends have been expanded to include full statistical reporting and replicate information.

All figure labelling inconsistencies have been corrected, and discrepancies between datasets (including those noted between Figures 1 and 5) have been carefully reviewed and clarified within the revised text.

Regarding mechanism of action, we agree that this is an important aspect. While a complete structural or biochemical mechanism remains beyond the scope of the current study, we have strengthened this section by refining our interpretation of the existing biochemical and biophysical data and clearly delineating what is supported experimentally versus what remains speculative. This provides a more balanced and transparent account of the current level of mechanistic understanding while outlining directions for future work.

## **Responses to reviewer 1**

### Reviewer 1 major comments

*Unfortunately, when patients present to the ophthalmologist with a Pseudomonas aeruginosa corneal infection, there is already, most likely, a corneal infiltrate present. Therefore, immediate treatment after infection prevents corneal damage from the bacterial virulence factors rather than treating damage already done. It would be prudent to include studies in which there was an established infection in the mouse corneas to determine whether the ExoU inhibitors have any enhanced effect on corneas with and without concurrent antibiotic treatment. This is the stated goal for the use of these inhibitors.*

**Response:** We thank the reviewer for highlighting this important translational consideration and agree that evaluating efficacy in established infection is a key next step. We acknowledge that our current *in vivo* model reflects early intervention and does not fully recapitulate clinical scenarios. However, the therapeutic rationale for ExoU inhibition is to limit ongoing tissue damage driven by continued toxin delivery, even after infection has been initiated, rather than to reverse existing damage.

We considered incorporating delayed-treatment models *in vivo*; however, these introduce substantial experimental variability, including heterogeneity in infection progression and challenges in standardising the timing and severity of disease prior to treatment. In addition, such approaches raise ethical considerations regarding prolonged untreated infection and increased animal burden, which we sought to minimise in accordance with the principles of the 3Rs.

Our *in vitro* scratch assays (Figure 1D) partially address this point, demonstrating that ExoU inhibitors retain efficacy in combination with sub-MIC₅₀ moxifloxacin, supporting their compatibility with antibiotic therapy.

We agree that delayed treatment and combination therapy studies *in vivo* will be important to fully establish translational potential. To clarify this point, we have revised the Discussion (in particular, lines 921-929) to explicitly frame the present study as establishing a mechanistic and preclinical foundation for subsequent translational studies, including delayed-treatment paradigms and combination therapy with standard antibiotics.

### Reviewer 1 minor comments

**Comment**: *It appears that there is missing text between lines 88 and 89 in the Introduction.*

**Response**: We thank the reviewer for noting this omission. The missing text has now been added.

**Comment**: *Lines 86-88 in the Introduction are very similar to lines 75-77. One of these statements should be eliminated.*

Response: We agree and have removed the redundant text to improve clarity and conciseness.

**Comment**: *It appears that the labeling of Figures 2A, 2B, 2C, and 2D is incorrect compared to what is in the text and legend. Figure 2E is not labeled. Labeling is also incorrect for Figures 1A, 1B, 1C, and 1D compared to what is in the text and legend. Figure 1E is not labeled.*

**Response**: We thank the reviewer for highlighting these inconsistencies. All figure labels have been carefully reviewed and corrected to ensure consistency between figures, legends, and the main text.

**Comment:** *In the ex vivo porcine corneal model, it is unclear as to how epithelial ulceration can be assessed when the corneal epithelium is debrided. This must be clarified.*

**Response:** We agree that this required clarification. In our model, the corneal epithelium is partially disrupted using an ethanol treatment and brief debriding (<https://doi.org/10.1371/journal.pone.0319911>), which loosens but does not completely remove the epithelial layer. Importantly, in the viable *ex vivo* tissue, the epithelium retains the capacity to regenerate over time. Accordingly, “ulceration” in this context reflects a combination of epithelial disruption and underlying stromal degradation resulting from infection, which manifests as increased corneal opacity and structural disorganisation rather than complete epithelial loss alone. In lines 623-631 we have clarified this point.

**Comment***: Lines 645 and 676: There is discussion about “blinded” observers. “Blinded” should not be used in ophthalmic research papers. “Masked” is a more appropriate term.*

Response: We thank the reviewer for this correction and have replaced “blinded” with “masked” throughout the manuscript.

**Comment:** *The authors should also discuss the clinical use of polymyxin B, which is a treatment for P. aeruginosa keratitis, and whether clinical signs of infection are reduced more with polymyxin B compared with other common treatments such as topical ciprofloxacin or tobramycin.*

**Response:** We thank the reviewer for this important suggestion. We are not aware of studies directly comparing polymyxin B with fluoroquinolones (e.g., ciprofloxacin or moxifloxacin) specifically in *P. aeruginosa* keratitis. While these agents have been evaluated in broader microbial keratitis cohorts, pathogen-specific comparative data remain limited. Furthermore, differences in MIC values and pharmacodynamic properties between these agents complicate direct comparison across studies.

We have now expanded the Discussion (lines 829-837) to address the clinical use of polymyxin B, including its role in topical therapy for *P. aeruginosa* infections, and to highlight the challenges in comparing efficacy across antibiotic classes. We also note that variability between clinical isolates and potential interactions between agents (ranging from additive to antagonistic effects) further complicate interpretation. Where appropriate, we have referenced relevant literature, including our previous work examining aminoglycoside activity in this context.

## **Responses to reviewer 2**

*Overall this is a very exciting and interesting study which outlines the identification of novel antivirulence activities associated with several FDA approved drugs. This is frontier area of research and as a result this study with respect to design and application. The study is expansive and detailed in that the inhibitors are explored in detail, with suggested mechanisms of action provided and thorough in vivo and ex vivo evidence of efficacy. The manuscript overall however is poorly written with some sections having relatively little to know explanation as to why certain assays were being carried out and/or lack interpretation of the data. This makes it a very dense read with the reader having to constantly interpret to figures to attempt to deduce how conclusions are being drawn. This can be addressed however with a thorough rewriting of the manuscript with clarity as central focus. There are some areas when there is a lack of reproducibility in the efficacy of the combinations for example: In Figure 5 C and D there is no difference between the DMSO control and the Pol or Zp treatment for strain PA103 but in Figure 1D there is a clear difference.*

**Response**: We kindly thank the reviewer for their positive assessment of the study and for highlighting the need to improve clarity and data interpretation.

In response, we have undertaken extensive revision of the manuscript to improve readability and narrative flow. Specifically, we have: streamlined the introduction to better contextualise the study and its rationale, added an overview paragraph at the beginning of the results section to guide the reader, clarified the rationale for each assay within the results, and expanded data interpretation throughout. In addition, all figure legends have been revised to include full experimental details, replicate numbers, and statistical analyses.

Regarding the apparent discrepancy between Figure 1D and Figure 5C for strain PA103, we thank the reviewer for highlighting this point. These experiments employ fundamentally different assay formats, which account for the observed differences in treatment effects.

Figure 1D uses a scratch and infection assay in fully confluent epithelial monolayers, where mechanical disruption facilitates rapid bacterial access and enhances ExoU-dependent cytotoxicity. Cell death is quantified by ethidium homodimer uptake, providing a direct measure of membrane permeabilisation and acute lysis.

In contrast, Figure 5 employed a high-content imaging assay in subconfluent (~80%) epithelial cells, resulting in distinct infection dynamics. In this assay, viability was assessed via calcein-AM retention, which reflects live-cell area rather than direct lytic events. Fully confluent epithelial layers are relatively resistant to *P. aeruginosa* infection, necessitating this alternative format for high-throughput analysis across multiple clinical isolates.

These differences in cell density, infection dynamics, and readout (acute lysis vs. live-cell area) resulted in distinct sensitivity to ExoU-mediated effects and, consequently, to inhibitor activity. We have now clarified these distinctions in the results (lines 527-532).

### Reviewer 2 major comments

**Comment:** *There are some areas when there is a lack of reproducibility… e.g. Figure 5C/D vs Figure 1D for PA103.*

**Response:** We thank the reviewer for highlighting this point. The apparent discrepancy arises from differences in assay design rather than lack of reproducibility as detailed in our response above. We have now clarified these distinctions in the Results section to avoid misinterpretation.

**Comment:** *The narrative is difficult to follow. There is a lot of information given out of context, with no explanation of the rationale for experiments, explanation of techniques used, objective of the experiment or reference back to information that was previously provided (e.g. in the introduction section). Equally there is a lack of interpretation of the data within the results sections. For all figure legends please include the number of biological and technical replicates and apply statistical analysis to all data. Include in the legend what the statistical test was and what was considered significant. It is also a bit odd to present stats in (screenshot) tables instead of using the standard p-value indication with *.*

**Response:** We thank the reviewer for these important comments. The manuscript has been extensively revised to improve clarity, structure, and data presentation.

Specifically, we have: streamlined the Introduction to better contextualise the study, added an overview paragraph at the beginning of the results section to guide the reader, clarified the rationale and objective of each experimental approach and expanded interpretation within the results to reduce reliance on figure inspection,

All figure legends have been revised to include the number of biological and technical replicates, statistical tests used, and definitions of significance. Statistical presentation has been standardised throughout using conventional notation (e.g. *P* values and asterisk-based significance), and previously embedded “screenshot-style” tables have been removed.

**Comment:** *The 3 compounds selected, Pol, Zp and Bis, are randomly used in pairs (all possible or only some) or triple combination only (no pairs tested) without apparent reason. This has to be homogenised.*

**Response:** We agree with the reviewer and have addressed this by performing additional experiments to systematically evaluate all pairwise combinations (Bis+Zp, Bis+Pol, Zp+Pol), as well as the triple combination (Bis+Zp+Pol), across relevant assays, including the high-content microscopy screen to determine efficacy across clinical isolates (Figure 5 and Supplementary Figure 9), providing a more consistent and comprehensive assessment of combinatorial effects. The rationale for combination testing has also been clarified in the results section.

**Comment:** *Some controls are missing. In graphs, there are indications of non-infected controls, untreated infected controls or DMSO-treated controls. Non-infected, treated controls need to be added, even if just as supplementary figures.*

**Response:** We thank the reviewer for this suggestion. Non-infected controls for Figure 5 are now included in Supplementary Figures 9A-G. compound-treated controls have been included to assess any direct effects of the inhibitors on host cells in the absence of infection.

### Reviewer 2 minor comments

**Comment:** *2024 WHO priority list.*

**Response:** We thank the reviewer for this comment. We have updated the text and reference to reflect the 2024-2025 WHO priority pathogen list. Carbapenem-resistant *P. aeruginosa* remains classified as a critical priority pathogen, and the wording has been revised to reflect this distinction.

**Comment:** *Line 89 begins with citations*

**Response:** Corrected. The sentence has been rewritten to improve clarity and flow.

**Comment:** *T3SS literature expansion.*

**Response:** We agree and have expanded this section to better contextualise our findings within the broader field of T3SS inhibition. The suggested references (PMIDs: 38686217; 20176902; 25638499) have been incorporated (lines 112-131).

**Comment:** *Figure 1A compound cluster*

**Response:** We thank the reviewer for this observation. The identities of all screened compounds, including those within this cluster, are provided in Supplementary Table 1 (CSV format). This apparent cluster originates from a single screening plate that showed a modest shift in overall ExoU activity relative to the plate median, consistent with normal plate-to-plate variability; however, all plates met predefined quality control criteria. None of the compounds within this cluster met our hit-selection thresholds, and follow-up dose response assays did not confirm significant ExoU inhibition for these compounds. To improve clarity, we have now highlighted all validated hits in the supplementary table and explicitly refer to this in the main text (lines 214-217).

**Comment:** *Figure 1D abbreviations*

**Response:** All abbreviations are now defined in the figure legend and corrected in the text.

**Comment:** *Supplementary Figure S1 labelling.*

**Response:** Corrected. Gel lanes are now clearly labelled.

**Comment:** *ExoU concentration rationale*

**Response:** The ExoU concentration was selected based on prior optimisation to ensure robust and reproducible phospholipase activity within the linear range of the assay while avoiding substrate depletion. Increasing the enzyme concentration would cause the reaction to proceed too quickly.

**Comment:** *Full screen availability*

**Response:** We agree and have made the full screening dataset available in the Supplementary Table 1 (CSV format), including all initial and validated hits.

**Comment:** *Undefined abbreviations*

**Response:** All abbreviations are now defined at first use in the main text.

**Comment:** *Description of hits*

**Response:** We thank the reviewer for this suggestion. We have expanded the text to highlight polymyxin B’s clinical use as an antibiotic (in both the results and discussion) with known nephron/neurotoxicity at systemic levels, and that chlorhexidine is a widely used antiseptic.

**Comment:** *Use of moxifloxacin vs MOI reduction*

**Response:** We used sub-MIC₅₀ moxifloxacin to partially suppress bacterial growth while maintaining sufficient infection pressure to observe ExoU-dependent cytotoxicity. Reducing MOI alone does not equivalently modulate toxin delivery dynamics. To address potential confounding effects, we directly compared inhibitor activity in the presence and absence of moxifloxacin and observed no significant differences, indicating that the anti-virulence effects are not driven by synergistic antibacterial activity. Compounds in combination did not affect bacterial viability (supplementary Figure 3B). This has now been clarified in the results (lines 232-257).

**Comment:** *Use of ExoU-negative strains*

**Response:** We agree and note that ExoS⁺ (ExoU-negative) clinical isolates were included in our study. These strains did not exhibit rapid cytotoxicity and showed minimal response to inhibitor treatment, supporting the specificity of the compounds for ExoU-mediated effects. This has now been more clearly emphasised in the Results.

**Comment:** *Bactericidal activity conditions and presentation*

**Response:** We thank the reviewer for this comment. We acknowledge that cation-adjusted Mueller-Hinton broth is the standard for susceptibility testing; however, our primary objective was to assess compound activity under conditions relevant to infection assays (cell culture media). We have clarified this rationale in the results and discussion.

**Comment:** *Supplementary Figure 3 statistics.*

**Response:** Statistical analysis has now been applied.

**Comment:** *Zp cellular entry*

**Response:** This is an important question. Zinc pyrithione is well documented to function as a zinc ionophore, facilitating zinc entry into eukaryotic cells. We have now cited relevant literature supporting intracellular activity (lines 818-828). While entry into bacterial cells remains possible, this was not directly assessed in the current study and is now acknowledged as a limitation in the Discussion.

**Comment:** *Figure 2 labelling panels D and E.*

**Response:** Corrected.

**Comment:** *Bismuth vs zinc uptake*

**Response:** We agree that this is an important point. While our data suggest differential behaviour between bismuth- and zinc-based compounds, we have not directly assessed cellular uptake of bismuth species. We demonstrate that bismuth subcitrate induces lysosomal localisation of intracellular ExoU in transfected HEK293T cells, which strongly suggests that this compound engages ExoU within the cell. This has now been clarified, and the mechanistic interpretation has been tempered accordingly.

**Comment:** *Figure 3B clarity*

**Response:** We thank the reviewer for this detailed and helpful comment. We have revised both the figure and the accompanying text to improve clarity and interpretation. Lane numbers have now been added directly to the figure to align with references in the text. Molecular weight markers (kDa) are clearly indicated, with 250 kDa as the upper reference band. The figure legend has been expanded to fully describe all conditions. The Results section has been revised to provide clearer interpretation of the banding patterns and to explicitly link observed molecular weights to likely oligomerisation states.

**Comment:** *Missing pair combinations*

**Response:** We agree and have now included all pairwise combinations in Figure 3C.

**Comment:** *L347-348: what value of activity would be the control to compare these activities to?*

**Response:** This is relative to the DMSO-treated control (defined as 100% ExoU activity), which we have now clarified. When combined, the combination displayed markedly enhanced potency. This shift is consistent with complementary mechanisms of action and provided the rationale for assessing inhibitor combinations in subsequent efficacy studies.

**Comment:** *Bactericidal activity of combinations*

**Response:**
We have now assessed and included bactericidal activity data for the combinations (Supplementary Figure 8), demonstrating that the observed effects are not driven by enhanced bacterial killing.

**Comment:** *Figure 4 concentration choice*

**Response:** The concentration of 5 µM was selected based on our prior dose-response and cytotoxicity data, as a concentration at which all three compounds retain measurable inhibitory activity while remaining below cytotoxic thresholds in mammalian cells (Supplementary Figure 5). This allowed for direct comparison of their mechanistic effects under conditions that are both biologically relevant and non-toxic. These experiments were performed in a HEK293T transfection system in the absence of *P. aeruginosa*, and therefore were designed to interrogate compound effects on ExoU activity and localisation independently of bacterial infection dynamics. Using a single, sub-cytotoxic concentration across all treatments ensured consistency and enabled clearer interpretation of compound-specific mechanisms. We have now clarified this rationale in the Results section.

**Comment:** *Protein expression wording*

**Response:** Corrected.

**Comment:** *Ubiquitin interpretation*

**Response:** We have clarified the interpretation of ubiquitin species in the gel and revised the text.

**Comment:** *L396: what is the rationale for this hypothesis? Even though it is demonstrated how the authors get to this idea, it is very hard to follow if it is not better explained at this point.*

**Response:** We have now expanded this section to better explain the basis of our hypothesis (lines 440-446). Specifically, we observed that treatment with Bis and Pol reduced both the cellular abundance and the membrane localisation of GFP-ExoU. As previous studies have shown that ExoU turnover is not dependent on proteasomal degradation, we reasoned that an alternative degradation pathway must be involved. Given prior reports that ExoU traffics through endosomal and lysosomal compartments (PMID: 16882033; 22496657), we hypothesised that these compounds may promote lysosome-dependent degradation of ExoU. This provided the rationale for subsequently investigating lysosomal involvement in ExoU clearance.

**Comment:** *Line 413 Section starting here needs to be completely rewritten to accurately explain the assays and interpret the findings. Figure 5 needs statistics throughout. The differences look modest at best so it would be important to understand how the authors are determining an effect and what the threshold for protection is. For example in Figure 5D I am struggling to understand how 48% of these are demonstrating protection, particularly as error bars overlap on nearly all of the strains shown. The data in supplemental table 2 is not helpful for this. Pairwise comparisons of each isolate with and without treatment at 4.5 hours is needed*

**Response:** We agree and have substantially revised this section. Statistical analyses have been added throughout, and pairwise along with the triple combination comparisons for each isolate at 4.5 h are now included (Supplementary Data). Thresholds for defining “protection” have been clearly stated.

**Comment:** *Missing Pol+Zp combination*

**Response:** This has now been included in the revised manuscript.

**Comment:** *Why is there no difference in Figure 5 C and D for strain PA103? When in Figure 1D there is a clear difference. This is a concern.*

**Response:** As noted above, this reflects differences in assay format and has now been clarified in the Results.

**Comment:** *Triple combination omission*

**Response:** We have now included the triple combination in this assay.

**Comment:** *Mechanistic interpretation*

**Response:** We have revised this section to better reflect the possibility of additional mechanisms and have tempered our conclusions accordingly.

**Comment:** *Protection thresholds*

**Response:** Thresholds for protection have now been explicitly defined in the Methods and Results.

**Comment:** *Triple combination safety*

**Response:** We thank the reviewer for this important point and agree that evaluation of the safety of the triple combination will be an essential step for further translational development. In the current study, we assessed the cytotoxicity of each compound individually (Supplementary Figure S4), demonstrating that all three agents are well tolerated at concentrations exceeding those used in our functional assays.

We have not performed a formal assessment of the triple combination, as this would require a comprehensive and systematic toxicological evaluation that is beyond the scope of the present study. We note that the primary aim of this work is to establish mechanistic insight and proof-of-concept efficacy for ExoU inhibition, rather than to provide a full preclinical safety assessment.

Importantly, the concentrations used in combination experiments are below the individual cytotoxic thresholds, and no overt toxicity was observed across our in vitro, ex vivo, or in vivo models. We have now revised the Discussion to explicitly acknowledge that detailed safety evaluation of combination regimens, including the triple therapy, will be an important focus of future work.

**Comment:** *Supplementary Figure 8. Needs to be reformatted for greater clarity.*

**Response:** Supplementary Figure (now 9) has been completely reformatted for clarity.

**Comment:** *Supplementary figure 10 should say PAO1 not PA01.*

**Response:** Corrected.

**Comment:** *Figure 7: No details on how many replicates in legends and it appears only 5 Galleria in total were evaluated, whereas the standard in the field would be 10 Galleria per condition and three independent biological replicates resulting in n=30. If the maximum health index score was 10 as per methods then why is the Y axis going to 11? Also it appears that there is a significant decrease in the health index score with the mice given PBS only. This is a worry as typically you would expect <10% mortality in this group and due to overlapping bars in Panel C it is not possible to see what the final survival was for the PBS only group.*

**Response:** We thank the reviewer for this important comment regarding experimental design. *Galleria* experiments were performed using 2 larvae biological replicates per condition across 5 independent experiments. We acknowledge that this is lower than the number of larvae per condition typically used in some studies. However, the repeated independent experimental design provides reproducibility across experiments and supports the observed trends. We have revised the results to temper interpretation of these data as supportive of in vivo-like activity rather than definitive efficacy. Importantly, the conclusions of the study are additionally supported by complementary *ex vivo* and mouse infection models.

We have revised the figure legends to include replicate numbers and clarified experimental design in the text. Axis scaling and data presentation have been corrected. We note that PBS-injected controls modest decline likely reflected injection-associated stress rather than infection, which we have now explained in the results.

**Comment:** *L479-503: This section does not make much sense after presenting all the previous results (ExoU specificity, ExoS+ strains not affected, etc.).*

**Response:** This section has been rewritten to improve clarity.

**Comment:** *L516: Pa in italics.*

**Response:** Corrected.

**Comment:** *Fig7C: orphan symbols in the legend and explanation of this panel missing in the legend. For panel B, left and right in the legend should be top and bottom?*

**Response:** We thank the reviewer for this helpful suggestion. The Figure 7 legend has been revised to improve clarity. Specifically, we have replaced “left” and “right” with “top” and “bottom” to accurately reflect the layout of panel B. In addition, we have expanded the description of panel C to explicitly define the survival curves and treatment groups shown. All symbols and annotations in panel C are now clearly described in the legend to avoid ambiguity.

**Comment:** *L596, L611: This is odd because looking at the graph is seems that the final mortality is not very close to the uninfected control.*

**Response:** We agree that the original wording may have overstated the similarity between the triple combination treatment and uninfected controls. While the statistical analysis indicated no significant difference at the 40 h endpoint, the survival curves and variability between larvae clearly show that outcomes do not fully return to baseline.

We have therefore revised the Results section to clarify this point. Specifically, we now state that although the triple combination improved survival relative to infected controls, it did not fully restore outcomes to those observed in uninfected larvae, and that variability remained high. This change ensures that the interpretation more accurately reflects the data presented in Figure 7.

**Comment:** *Line 608: How can the authors claim all larvae in the triple treatment group remained healthy at 32 hours, when in Figure 7b Lower panel the HIS is at 3/10?*

**Response:** We thank the reviewer for highlighting this inconsistency. We agree that the original wording was inaccurate and may have been misleading. The term “healthy” was used too loosely and does not accurately reflect the health index scores shown in Figure 7B.

We have revised the text to clarify that, while the triple combination delayed disease progression and preserved larval viability for longer compared to other treatment groups, health scores had already declined by 32 h and did not indicate full health. The updated wording now more accurately describes partial protection rather than sustained health.

**Comment:** *Line 616: The writing, clarity and interpretation of the data needs to be greatly improved in this section.* 
**Response:** This section has been extensively revised.

**Comment:** *Overall, resistance to these approaches should also be dealt with properly in the discussion at least as while the probability of resistance is less, there is still evidence of resistance to T3SS inhibitors (PMID: 24468789).*

**Response:** We agree and have expanded the Discussion (888-898) to address the potential for resistance to antivirulence therapies.

**Comment:** *Missing Figure 9*

**Response:** This typo has been corrected.

## **Responses to reviewer 3**

*In this manuscript the authors present an extraordinary array of work and assays to screen for and characterize inhibitors of a T3SS-phospholipase encoded by Pseudomonas aeruginosa. The strengths of the paper are the quantitative aspects, the fact that the drugs are already approved for human use and the demonstration that they can be used synergistically in certain models. Importantly, they target drug use for topical application to eye infections, where the majority of isolates express this specific virulence determinant. The manuscript is well written but lengthy and dense. The organization is difficult to follow at times with a fair amount of materials and methods as well as discussion/speculation mixed into the results section. The rationale for using 5 different cellular and animal models of infection, each seemingly measuring a different parameter using different imaging analysis is unclear. Overall, the manuscript could be improved by a focused re-organization of streamlined data that can be mechanistically interpreted.*

**Response**: We kindly thank the reviewer for their thoughtful and constructive assessment of our work, and for highlighting its strengths, particularly the quantitative approach and repurposing of clinically approved compounds.

We agree with the concerns raised regarding the density, organisation and clarity of the manuscript. We also agree that the rationale for the use of multiple infection models needed to be discussed and articulated better. In response, we have undertaken a substantial revision of the manuscript. This includes extensive re-writing to improve clarity and readability and clearer separation of results, discussion and methods.

We have restructured the results section to provide a more coherent narrative, including the addition of an introductory overview to guide the reader through the experimental workflow and rationale for each model system. We have also streamlined the presentation of data and expanded interpretation within the results to ensure that key mechanistic insights are clearly conveyed, rather than inferred.

We believe these revisions have substantially improved the accessibility, focus, and overall narrative flow of the manuscript.

### Reviewer 3 major comments

**Comment:** *Figure 1D. It seems Mox inclusion does make a difference, especially in combination with other compounds in terms of reducing cellular lysis. Could polymyxin B and the moxifloxacin be working synergistically to kill bacteria? Inhibition of replication is a known inhibitor of T3SS expression. It appears that the IC50 of polymyxin B for OD600 reduction, and in theory, bacterial lysis for PA103 is 0.75 /2.1 µM and the dose in the cell assay (1D) is 1 µM. These concentrations are close so the interpretation should be tempered.*

**Response**: This is an important observation. We agree that, given the proximity of the polymyxin B concentration used in the cellular assay (1 µM) to its measured IC₅₀ for growth inhibition, it is important to carefully exclude potential antibacterial or synergistic effects as an explanation for the observed protection. To address this, we performed additional bacterial growth assays in the presence of subMIC moxifloxacin (2.5 µM) with and without ExoU inhibitors (Supplementary Figure 3B). These data show that the inclusion of Bis, Zp, or Pol does not significantly alter bacterial viability beyond the effect of subMIC moxifloxacin alone, indicating that the observed reduction in cytotoxicity is not due to enhanced bactericidal or bacteriostatic activity.

Furthermore, consistent with this, we observe no reduction in bacterial burden in *ex vivo* and *in vivo* infection models (Figures 6 and 8), despite clear protection of host cells and tissues. Together, these findings support the interpretation that the effects observed in Figure 1D primarily reflect inhibition of ExoU-mediated cytotoxicity rather than indirect effects on bacterial growth or T3SS expression. We have clarified this point in the Results and Discussion and tempered the interpretation accordingly.

**Comment:** *Line 193 "none of the inhibitors displayed bactericidal activity at the concentrations tested..." While this statement is true, it can be misleading. Perhaps it might be best to state that yes some of the inhibitors displayed bactericidal activity, but the phospholipase inhibition occurred at inhibitor concentrations that exhibited no or minimal anti-bacterial activity.*

**Response**. We thank the reviewer for this helpful suggestion and agree that the original phrasing could be misleading. While we did not observe bactericidal activity at the concentrations used in the ExoU inhibition and cellular assays, we acknowledge that some compounds, particularly Zp and Pol, exhibit antibacterial activity at higher concentrations.

We have therefore revised the text to clarify that ExoU inhibition occurs at concentrations that do not measurably impact bacterial viability under the conditions tested. This distinction is now made explicit to emphasise that the protective effects observed are attributable to inhibition of ExoU-mediated cytotoxicity rather than antibacterial activity.

**Comment:** *Thermal stability curves are difficult to interpret because they depend greatly on the environment. The conditions the authors use are a relativey high pH (>8) with glycerol. PIP2 is unlikely to form micelles at 5 µM and there is no ubiquitin in the mix. PIP2 could be stabilizing a folded structure or aggregating the enzyme making it appear to be more or less stable since this assay measures intrinsic fluorescence. Can the authors truly tell the difference between aggregation and oligomerization and how each impacts the enzymatic activity?*

**Response:** We thank the reviewer for these insightful comments and agree that DSF data must be interpreted with caution, particularly given their inability to directly distinguish between aggregation and defined oligomerisation states.

The assay conditions used here (pH 8.2) are within the range commonly employed for protein stability measurements and were selected to maintain ExoU solubility and activity. However, we acknowledge that factors such as PIP₂ behaviour in solution and the absence of ubiquitin may influence the observed thermal profiles. In particular, we agree that changes in intrinsic fluorescence could reflect altered folding, aggregation, or higher-order assembly rather than discrete oligomerisation alone.

For this reason, we have interpreted the DSF data conservatively and in conjunction with orthogonal approaches. Specifically, BS³ crosslinking experiments (Figure 3B) provide independent evidence of compound-dependent changes in ExoU assembly state, allowing us to distinguish between monomeric, dimeric, and higher-order species under comparable conditions. Importantly, these structural changes correlate with functional inhibition of phospholipase activity, supporting a mechanistic link between altered assembly and reduced enzymatic activity.

We have revised the text to clarify these limitations and to avoid overinterpretation of the DSF data in isolation.

**Comment**: *Purification and enzyme activity on the oligomeric forms would likely answer the question especially since an enhancer of activity also seems to augment oligomerization (aggregation). The activity assay seems to be sensitive enough to perform this critical assessment and although activities are shown, the physical nature of the oligomer or aggregate has not been characterized.*

**Response**: This is an insightful suggestion and agree that direct isolation and biochemical characterisation of defined ExoU oligomeric species would provide valuable mechanistic insight into how these compounds modulate enzyme activity.

However, the isolation of discrete oligomeric versus aggregated species under these conditions is technically challenging, particularly given the dynamic and potentially heterogeneous nature of ExoU assemblies in the presence of lipids and small molecules.

Instead, we have taken a complementary approach by combining orthogonal assays. BS³ crosslinking experiments (Figure 3B) provide evidence for compound-dependent shifts in ExoU assembly state, while phospholipase activity assays demonstrate corresponding functional inhibition. The correlation between altered assembly profiles and reduced enzymatic activity supports a mechanistic link between compound-induced changes in ExoU organisation and loss of function, even without physical separation of individual oligomeric species.

We agree that detailed biochemical characterisation of isolated oligomeric states represents an important avenue for future work.

**Comment**: *Live cell area seems to contradict some of the early data suggesting inhibition of toxicity. Is this due to the multiple effectors being injected? For example, Bis is highly effective at maintenance of the live cell area while Pol and Zp aren't. Pol and Zp seemed to work better in other assays.*

**Response:** We thank the reviewer for this perceptive observation. The apparent differences in inhibitor efficacy across assays reflect the distinct experimental formats and readouts used rather than contradictory biological effects. In the live-cell area assay (Figure 5), HCE-T cells are infected at ~80% confluence and cytotoxicity is quantified based on calcein-AM retention, providing a measure of overall cell viability in a partially confluent monolayer. In contrast, the earlier assays (scratch-based infection models) use fully confluent epithelial monolayers subjected to mechanical disruption, with cytotoxicity assessed via ethidium homodimer uptake, which specifically reports acute membrane rupture and cell lysis.

These differences in cell density, infection dynamics, and assay sensitivity can influence the apparent relative efficacy of individual compounds. In particular, Bis appears more effective in preserving global monolayer integrity under partially confluent conditions, whereas Zp and Pol show stronger effects in assays that more directly measure acute ExoU-driven lysis. Importantly, all assays consistently show enhanced protection with combination treatments, which is the central conclusion of the study. We have clarified these differences in the results.

**Comment:** *The authors test several compounds, salts or derivatives to address the mechanisms* *mediating inhibition of phospholipase or toxic activity in cells. This approach eliminates compounds or eliminates chemical entities but seems to never address the biochemical mechanisms mediating enzymatic inactivation. How do these compounds interact with the enzyme? There is reasonable speculation (e.g. "both peptide length and charge, as well as the cyclic structure are critical for ExoU inhibition") but no definitive series of experiments or models to drive inhibition to the mechanistic level.*

**Response:** We agree with the reviewer that a definitive, atomic-level mechanism of inhibition would ideally be established through structural and/or biophysical studies of ExoU in complex with each compound. However, this lies beyond the scope of the current study, which focuses on functional and mechanistic inference across biochemical, cellular, and infection models.

At present, structural information for ExoU remains limited to the ExoU-SpcU chaperone complex, and to our knowledge no high-resolution structure of full-length, activated ExoU (membrane-bound, ubiquitin- or PIP₂-bound states) is available. This, combined with the known conformational plasticity of ExoU during activation and membrane association, makes co-structural characterisation of inhibitor-bound states particularly challenging at this stage.

To address mechanism as far as experimentally feasible within these constraints, we have instead combined multiple orthogonal approaches (phospholipase activity assays, cellular toxicity readouts, BS³ crosslinking, and trafficking/localisation studies). Together, these data support distinct inhibitory behaviours consistent with disruption of catalytic activity, altered oligomerisation states, and/or mislocalisation within host cells.

We have revised the discussion to clarify that these mechanistic models are inferred from integrated functional datasets.

**Comment:** *Lines 294-306. The discussion of changes in stability during heat denaturation in the presence and absence of compounds and PIP2 suggests that PIP2 is absolutely required for a stable conformation and hence enzymatic activity. Is this true as there is biophysical literature characterizing this enzyme in which no PIP2 is included in their enzyme assays. This is confusing.*

**Response:** We thank the reviewer for this important clarification. We agree that our original wording could be misinterpreted as implying that PIP₂ is absolutely required for ExoU to adopt a stable or active conformation, which is not consistent with prior biochemical studies.

Previous work has shown that ExoU retains measurable, albeit far weaker phospholipase activity in the absence of PIP₂. More broadly, PIP₂ is now understood to function primarily as an activation and membrane-targeting cofactor that promotes productive assembly at the membrane, rather than being strictly required for global protein stability.

We have revised this section to clarify that the thermal shift assay reports changes in apparent stability under assay conditions, which likely reflects PIP₂-dependent effects on conformational state, membrane-associated organisation, and/or oligomerisation propensity rather than absolute protein folding stability.

**Comment**: *Testing many clinical isolates is laudable. It may be too simplistic, however, to bin them into ExoS and ExoU expression strains. Other toxic factors and metabolites could be expressed during in vitro and in vivo infection. The inhibitors could be having effects on the eukaryotic cells or tissues, which is not discussed. Since the authors use many tissues and cells and different measurement parameters, it complicates the interpretation of the outcomes. Carefully consider the use of each model and provide some rationale for incorporating those data into the paper.*

**Response:** We agree that clinical *P. aeruginosa* isolates differ in multiple virulence determinants beyond ExoU/ExoS status, and that additional bacterial factors and host responses may contribute to the observed phenotypes in both in vitro and in vivo models.

In our study, we do not intend to imply that ExoU or ExoS alone fully explains isolate-specific pathogenicity. Rather, we use ExoU/ExoS classification as a functional framework to stratify isolates based on their dominant T3SS effector background, which is strongly associated with distinct and reproducible host cell phenotypes in our assay system. In particular, across the infection timeframes used here, ExoU+ isolates consistently induce rapid host cell lysis, whereas ExoS+ isolates predominantly induce cell rounding without acute lysis (Supplementary Videos 5-6). We have revised the results and discussion to clarify that other bacterial factors and host cell responses may contribute to the overall phenotype, and that our conclusions regarding inhibitor activity are based on comparative effects within this defined infection context rather than attribution of all cytotoxicity exclusively to ExoU. We also now more explicitly justify the use of multiple model systems as complementary readouts.

### Reviewer 3 minor comments

**Comment:** *The authors state that rExoU was made with a 6x-his tag at the N-terminus (lines 117-118) yet in the materials and methods they state the rExoU was fused to a C-terminal 6x His tag (lines 867-686). Please clarify or correct.*

**Response:** Thank you for spotting this. For ExoU, it has been corrected to C-terminal 6xhis tag.

Comment: *Line 89 begins with 2 citations?* 
**Response:** Corrected

**Comment:** *Formatting PLA2G4C (lines 213-226) and likely throughout.*

**Response:** Corrected

Comment: *Line 244-246. Might the Zp also need a carrier to enter PA103?*

**Response:** This is an insightful point. Zinc pyrithione (Zp) is a membrane-permeable metal ionophore and can enter Gram-negative bacteria, including *P. aeruginosa*, where it perturbs metal homeostasis. Although we demonstrate that Zp had no effect on T3SS gene expression (Supplementary Figure 10), bacterial uptake of Zp could, in principle, influence virulence mechanisms. With concentrations used in our study, Zp did not significantly affect bacterial growth or CFU. Furthermore, Zp did not inhibit ExoS-mediated phenotypes in *exoS⁺* strains, suggesting that general T3SS-dependent effector delivery remains functional. Our data support a model in which Zp primarily inhibits ExoU-mediated cytotoxicity, although we cannot exclude additional contributions from effects on bacterial physiology or effector delivery. We have clarified this point in the revised manuscript.

**Comment:** *Figure 2 may be mislabeled? No 2D, Line 270, 276 and no 2E, Line 272.*

**Response:** Corrected. 

**Comment:** *Line 292, weather?*

**Response:** Corrected.
