# Supplementary material for: Topical formulation of repurposed FDA-approved compounds inhibits Pseudomonas aeruginosa ExoU and improves corneal infection outcomes
Source: PLoS Pathog. 2026 Aug 3;22(8):e1014474. doi: 10.1371/journal.ppat.1014474 (PMC13450841; doi:10.1371/journal.ppat.1014474)
Supplement: Supplementary file 3 [file ppat.1014474.s020.docx]

We kindly thank the Editors and reviewers for their thorough and comprehensive second round of reviewing. We hope that our additional changes and inclusions have made our manuscript acceptable for publication.

**Response to Editors' Comments**

We have amended our Financial Disclosure statement. As the MRC grants (MR/X013502/1 and MR/K015931/1) supported the imaging infrastructure and equipment used in the study, these have instead been mentioned in the Acknowledgments section.

We have included all unaltered underlying western blot and gel images in the PDF file S1_raw_images.

**Response to reviewers' Comments**

**Part I – Summary**

Reviewer #1: Details regarding the mouse keratitis model are lacking as well as an inappropriate statistical analysis of the clinical data.

*Response. Please see responses below and in the methods of the manuscript. The statistical tests used were appropriate according to the experimental design and the distribution of the data.*

Reviewer #2: All my concerns have been dealt with and additional data has been included to support claims.

*Response. Many thanks.*

Reviewer #3: The authors have revised the manuscript to where a cursory reading allows a casual reader to pick up the main points. The testing is clearly comprehensive and the conclusions very similar: multiple drug combinations work better, the drugs inhibit ExoU phospholipase activity but don't affect bacterial growth or T3SS expression and have varying efficacy depending on the strain and the specific test. New data has been added making it a somewhat different paper than initially submitted. It is still quite a long read and has minor issues that should be corrected. While the authors are addressing an important human health problem, the significance seems limited to the use of drug cocktails rather than pursuing one of the mechanisms of action and improving drug efficacy.

*Response. Thank you. We have made further minor corrections*

**Part II – Major Issues**

**Reviewer #1**: This reviewer has concerns regarding the mouse keratitis model studies.
1. The number of animals per group is not stated.

*Response: the number animals per condition is now provided in tables 2 and 3.*

2. Were both eyes or one eye infected and treated?

*Response: one eye was infected and treated as per ethical practice. This has now been clarified in the methods.*

3. For the clinical scoring of the eyes, mean ± SD scores should not be presented for graded data. Median scores with interquartile ranges should be presented.

4. The clinical data for is not continuous. There are only 16 scores plus zero that can be used. Therefore, a nonparametric analysis should be used.

*Responses to 3 and 4.*

*We have, as requested, also included the median and IQRs in the tables and retained the mean (see below). Please note the closeness of the mean and median for the infected eyes.*

*We acknowledge that our data can only take on values 0, 0.25, 0.5.... 16, thus strictly speaking they are discrete. However, our data are on a wide scale (from 0 to 16, that is 64 values), i.e., in technical terms, the response random variable is defined on a sample space of 64 possible values. In such situations parametric methods are suitable, and often desired as they typically provide higher power. Therefore, in our case we used general linear model, and as per standard protocols, we also did a goodness-of-fit analysis of the model. Such analysis includes a residual analysis as a check for normality assumption, see the Q-Q plot below which shows the residuals are approximately on the 45 degree line, which is expected when the residuals are normally distributed.*

5. It is unclear what the values represent under “Condition” in Tables 2 and 3. These values must be defined.

*Response: As per the table legend, these are the probability values comparing the different treatment conditions.*

6. Figure 8A. What is the clinical score? Mean? Median? What do the error bars represent.

*Response. They are the mean value and standard deviation (now included in the legend and tables 2 and 3).*

7. How were the ulcer sizes determined? Was fluorescein staining used?

*Response. Please see the methods. Corneal disease severity was scored independently by two masked observers on two separate occasions using a previously described extended 16-point grading system [39, 40, 53]. This composite system evaluates four parameters: (i) area of opacity, (ii) density of central opacity, (iii) density of peripheral opacity, and (iv) epithelial surface integrity. Individual parameter scores were summed to generate a total score ranging from 0 (no infection) to 16 (severe infection). Fluorescein in not used in this grading system.*

Reviewer #2: none

Reviewer #3: NA

**Part III – Minor Issues**

Reviewer #1: For the *Galleria mellonella* studies, were the larvae injected with the ExoU inhibitors without infection to determine any potential toxicity?

*Response. The larvae were only injected with PBS-DMSO vehicle as this study was not a toxicokinetic study.*

Reviewer #2: None

Reviewer #3: 1.Line 103-104. Reference?

*We have now provided an appropriate references.*

2. A surprising number of compounds inhibit ExoU activity over 100%. For example, ibruprofen, 105.3%. Is this actually % ExoU activity rather than % inhibition?

*Referring to figure 1A and supplementary table 1, the values represent percentage ExoU activity, not inhibition. This is a comparison of ExoU activity in the presence of inhibitors with reference to DMSO controls, which represents 100% activity. We have now further clarified this in the figure and table legends.*

3. Line 339. The authors need to check the changes in thermal stability calculations. Either that or something is mislabeled (Supplementary Fig 7).

*The labels are correct. In the revised Supplementary Fig 7, we have provided all thermal denaturation profiles across our three experiments.*

4. Figure 3A. ∆Tm/˚C? How does panel A differ from the information shown in S Fig 7? ∆Tm
for Pol (relative to DMSO = -1.8˚C) -1.8/40.86 = -0.044? What does the Y axis mean?

*Supplementary figure 7 shows thermal denaturation profiles highlighting inflection points that were used to deduce melting temperature (T_m_). The bar chart in Figure 3A allows direct observation, across all repeat experiments, in the change in melting temperature (Δ T_m_), relative to stated controls (1% [v/v] DMSO ± PIP2).*

5. Supplementary Figure 10. Quantitative RT-PCR analysis suggest that the T3SS is transcribing at a basal level in LB medium. How does this relate to T3SS being expressed in cells and under tissue culture conditions? What is Vald?

*This is correct, we do observe a basal transcribing level of the T3SS in LB medium, which is enhanced by EGTA. We chose to perform these experiments in LB medium because it provides a controlled environment in which the direct effects of the inhibitors on T3SS gene expression can be assessed without the additional complexity introduced by host cells. Tissue culture systems contain numerous host-derived factors that can influence bacterial transcriptional responses, making it more difficult to distinguish direct effects on the bacterium from indirect effects mediated through host-pathogen interactions. Our intention was therefore to establish whether the compounds directly affect T3SS-associated transcription under defined conditions. We thank the reviewer for highlighting the "Vald" issue. Analysis of valdecoxib was included in the original version of Supplementary Figure 10 in error and does not form part of the final inhibitor panel evaluated in this study. The label has been removed from the revised figure.*
