# Supplementary material for: Topical formulation of repurposed FDA-approved compounds inhibits Pseudomonas aeruginosa ExoU and improves corneal infection outcomes
Source: PLoS Pathog. 2026 Aug 3;22(8):e1014474. doi: 10.1371/journal.ppat.1014474 (PMC13450841; doi:10.1371/journal.ppat.1014474)
Supplement: Peer review history

# Peer review: Decision Letter 0

**DOI:** 10.1371/journal.ppat.1014474.r001

**Authors:** Thomas Guillard, Patricia Bernal

24 Dec 2025

-->PPATHOGENS-D-25-02908

Topical formulation of repurposed FDA-approved compounds inhibits *pseudomonas aeruginosa*  ExoU and improves corneal infection outcomes

PLOS Pathogens

Dear Dr. Foulkes,

Thank you for submitting your manuscript to PLOS Pathogens. After careful consideration, we feel that it has merit but does not fully meet PLOS Pathogens's publication criteria as it currently stands. Therefore, we invite you to submit a revised version of the manuscript that addresses the points raised during the review process.

The reviewers agree that the study represents an exciting and comprehensive effort to identify novel antivirulence activities for FDA-approved drugs, describing it as a "frontier area of research" with a strong array of quantitative assays and detailed in vivo/ex vivo evidence. However, several major concerns must be addressed before acceptance. First, there is a significant need to improve the translational relevance of the work; reviewers noted that the study currently lacks evidence for the efficacy of these inhibitors in established infections, particularly when used in combination with standard antibiotics, which is the stated clinical goal. Second, the manuscript is considered overly dense and difficult to follow; the text should be reorganised to improve the focus and to clarify the rationale for using five different infection models and provide more direct interpretation of the data rather than leaving it to the reader. Finally, technical and mechanistic discrepancies must be addressed, including inconsistencies in figure labelling, reproducibility issues between different figures, and, more importantly, the lack of biochemical evidence regarding how these compounds interact with the enzyme.

Please submit your revised manuscript by Feb 22 2026 11:59PM. If you will need more time than this to complete your revisions, please reply to this message or contact the journal office at plospathogens@plos.org. When you're ready to submit your revision, log on to https://www.editorialmanager.com/ppathogens/ and select the 'Submissions Needing Revision' folder to locate your manuscript file.

Please include the following items when submitting your revised manuscript:

* A letter that responds to each point raised by the editor and reviewer(s). You should upload this letter as a separate file labeled 'Response to Reviewers'. This file does not need to include responses to any formatting updates and technical items listed in the 'Journal Requirements' section below.

* A marked-up copy of your manuscript that highlights changes made to the original version. You should upload this as a separate file labeled 'Revised Manuscript with Track Changes'.

* An unmarked version of your revised paper without tracked changes. You should upload this as a separate file labeled 'Manuscript'.

If you would like to make changes to your financial disclosure, competing interests statement, or data availability statement, please make these updates within the submission form at the time of resubmission. Guidelines for resubmitting your figure files are available below the reviewer comments at the end of this letter.

We look forward to receiving your revised manuscript.

Kind regards,

Patricia Bernal

Academic Editor

PLOS Pathogens

Thomas Guillard

Section Editor

PLOS Pathogens

Sumita Bhaduri-McIntosh

Editor-in-Chief

PLOS Pathogens

orcid.org/0000-0003-2946-9497

-->Michael Malim

Editor-in-Chief

PLOS Pathogens

orcid.org/0000-0002-7699-2064

**Journal Requirements:**

If the reviewer comments include a recommendation to cite specific previously published works, please review and evaluate these publications to determine whether they are relevant and should be cited. There is no requirement to cite these works unless the editor has indicated otherwise.

1) Please ensure that the CRediT author contributions listed for every co-author are completed accurately and in full.

At this stage, the following Authors/Authors require contributions: Daniel Morgan Foulkes, David G. Fernig, Keri McLean, Marie Held, John A. Harris, Yan Sun, Joanne L. Fothergill, Valerie Price, Dominic P. Byrne, Gabriela Czanner, Connie Tam, and Stephen B. Kaye. Please ensure that the full contributions of each author are acknowledged in the "Add/Edit/Remove Authors" section of our submission form.

The list of CRediT author contributions may be found here: https://journals.plos.org/plospathogens/s/authorship#loc-author-contributions

2) We ask that a manuscript source file is provided at Revision. Please upload your manuscript file as a .doc, .docx, .rtf or .tex. If you are providing a .tex file, please upload it under the item type u2018LaTeX Source Fileu2019 and leave your .pdf version as the item type u2018Manuscriptu2019.

3) Please provide an Author Summary. This should appear in your manuscript between the Abstract (if applicable) and the Introduction, and should be 150-200 words long. The aim should be to make your findings accessible to a wide audience that includes both scientists and non-scientists. Sample summaries can be found on our website under Submission Guidelines:

https://journals.plos.org/plospathogens/s/submission-guidelines#loc-parts-of-a-submission

4) We do not publish any copyright or trademark symbols that usually accompany proprietary names, eg ©,  ®, or TM  (e.g. next to drug or reagent names). Therefore please remove all instances of trademark/copyright symbols throughout the text, including:

- TM on page: 51.

5) Please upload all main figures as separate Figure files in .tif or .eps format. For more information about how to convert and format your figure files please see our guidelines:

https://journals.plos.org/plospathogens/s/figures

6) Some material included in your submission may be copyrighted. According to PLOSu2019s copyright policy, authors who use figures or other material (e.g., graphics, clipart, maps) from another author or copyright holder must demonstrate or obtain permission to publish this material under the Creative Commons Attribution 4.0 International (CC BY 4.0) License used by PLOS journals. Please closely review the details of PLOSu2019s copyright requirements here: PLOS Licenses and Copyright. If you need to request permissions from a copyright holder, you may use PLOS's Copyright Content Permission form.

Please respond directly to this email and provide any known details concerning your material's license terms and permissions required for reuse, even if you have not yet obtained copyright permissions or are unsure of your material's copyright compatibility. Once you have responded and addressed all other outstanding technical requirements, you may resubmit your manuscript within Editorial Manager.

Potential Copyright Issues:

i) Please confirm (a) that you are the photographer of 8B, or (b) provide written permission from the photographer to publish the photo(s) under our CC BY 4.0 license.

7) Please note that your Data Availability Statement is currently missing the DOI/accession number of each dataset OR a direct link to access each dataset. If your manuscript is accepted for publication, you will be asked to provide these details on a very short timeline. We therefore suggest that you provide this information now, though we will not hold up the peer review process if you are unable.

8) Please amend your detailed Financial Disclosure statement. This is published with the article. It must therefore be completed in full sentences and contain the exact wording you wish to be published.

1) State the initials, alongside each funding source, of each author to receive each grant. For example: "This work was supported by the National Institutes of Health (####### to AM; ###### to CJ) and the National Science Foundation (###### to AM)."

2) State what role the funders took in the study. If the funders had no role in your study, please state: "The funders had no role in study design, data collection and analysis, decision to publish, or preparation of the manuscript."

3) If any authors received a salary from any of your funders, please state which authors and which funders..

If you did not receive any funding for this study, please simply state: u201cThe authors received no specific funding for this work.u201d

**Reviewers' Comments:**

Reviewer's Responses to Questions

**Part I - Summary**

Please use this section to discuss strengths/weaknesses of study, novelty/significance, general execution and scholarship.

Reviewer #1: This manuscript is well-written and comprehensively performed. Unfortunately, the study is basic science in nature and does not have the translational aspect that many in ophthalmology demand. The goal is to use these inhibitors to supplement antibiotic treatment of Pseudomonas aeruginosa keratitis, which is not done. It is unfortunate that these studies were not included in this manuscript. Those studies would strengthen the manuscript.

Reviewer #2: Overall this is a very exciting and interesting study which outlines the identification of novel antivirulence activities associated with several FDA approved drugs. This is frontier area of research and as a result this study with respect to design and application. The study is expansive and detailed in that the inhibitors are explored in detail, with suggested mechanisms of action provided and thorough in vivo and ex vivo evidence of efficacy. The manuscript overall however is poorly written with some sections having relatively little to know explanation as to why certain assays were being carried out and/or lack interpretation of the data. This makes it a very dense read with the reader having to constantly interpret to figures to attempt to deduce how conclusions are being drawn. This can be addressed however with a thorough rewriting of the manuscript with clarity as central focus. There are some areas when there is a lack of reproducibility in the efficacy of the combinations for example: In Figure 5 C and D there is no difference between the DMSO control and the Pol or Zp treatment for strain PA103 but in Figure 1D there is a clear difference.

Reviewer #3: In this manuscript the authors present an extraordinary array of work and assays to screen for and characterize inhibitors of a T3SS-phospholipase encoded by Pseudomonas aeruginosa. The strengths of the paper are the quantitative aspects, the fact that the drugs are already approved for human use and the demonstration that they can be used synergistically in certain models. Importantly, they target drug use for topical application to eye infections, where the majority of isolates express this specific virulence determinant. The manuscript is well written but lengthy and dense. The organization is difficult to follow at times with a fair amount of materials and methods as well as discussion/speculation mixed into the results section. The rationale for using 5 different cellular and animal models of infection, each seemingly measuring a different parameter using different imaging analysis is unclear. Overall, the manuscript could be improved by a focused re-organization of streamlined data that can be mechanistically interpreted.

**********

**Part II – Major Issues: Key Experiments Required for Acceptance**

Please use this section to detail the key new experiments or modifications of existing experiments that should be absolutely required to validate study conclusions.

Generally, there should be no more than 3 such required experiments or major modifications for a "Major Revision" recommendation. If more than 3 experiments are necessary to validate the study conclusions, then you are encouraged to recommend "Reject".

Reviewer #1: Unfortunately, when patients present to the ophthalmologist with a Pseudomonas aeruginosa corneal infection, there is already, most likely, a corneal infiltrate present. Therefore, immediate treatment after infection prevents corneal damage from the bacterial virulence factors rather than treating damage already done. It would be prudent to include studies in which there was an established infection in the mouse corneas to determine whether the ExoU inhibitors have any enhanced effect on corneas with and without concurrent antibiotic treatment. This is the stated goal of for the use of these inhibitors.

Reviewer #2: • There are some areas when there is a lack of reproducibility in the efficacy of the combinations for example: In Figure 5 C and D there is no difference between the DMSO control and the Pol or Zp treatment for strain PA103 but in Figure 1D there is a clear difference. This needs to be clarified.

• The narrative is difficult to follow. There is a lot of information given out of context, with no explanation of the rationale for experiments, explanation of techniques used, objective of the experiment or reference back to information that was previously provided (e.g. in the introduction section). Equally there is a lack of interpretation of the data within the results sections. For all figure legends please include the number of biological and technical replicates and apply statistical analysis to all data. Include in the legend what the statistical test was and what was considered significant. It is also a bit odd to present stats in (screenshot) tables instead of using the standard p-value indication with *.

• The 3 compounds selected, Pol, Zp and Bis, are randomly used in pairs (all possible or only some) or triple combination only (no pairs tested) without apparent reason. This has to be homogenised.

• As far as I can see, some controls are missing. In graphs, there are indications of non-infected controls, untreated infected controls or DMSO-treated controls. Non-infected, treated controls need to be added, even if just as supplementary figures.

Reviewer #3: Substantive concerns

1. Figure 1D. It seems Mox inclusion does make a difference, especially in combination with other compounds in terms of reducing cellular lysis. Could polymyxin B and the moxifloxacin be working synergistically to kill bacteria? Inhibition of replication is a known inhibitor of T3SS expression. It appears that the IC50 of polymyxin B for OD600 reduction, and in theory, bacterial lysis for PA103 is 0.75 /2.1 µM and the dose in the cell assay (1D) is 1 µM. These concentrations are close so the interpretation should be tempered.

2. Line 193 "none of the inhibitors displayed bactericidal activity at the concentrations tested..." While this statement is true, it can be misleading. Perhaps it might be best to state that yes some of the inhibitors displayed bactericidal activity, but the phospholipase inhibition occurred at inhibitor concentrations that exhibited no or minimal anti-bacterial activity.

3. Thermal stability curves are difficult to interpret because they depend greatly on the environment. The conditions the authors use are a relativey high pH (>8) with glycerol. PIP2 is unlikely to form micelles at 5 µM and there is no ubiquitin in the mix. PIP2 could be stabilizing a folded structure or aggregating the enzyme making it appear to be more or less stable since this assay measures intrinsic fluorescence. Can the authors truly tell the difference between aggregation and oligomerization and how each impacts the enzymatic activity?

4. Purification and enzyme activity on the oligomeric forms would likely answer the question especially since an enhancer of activity also seems to augment oligomerization (aggregation). The activity assay seems to be sensitive enough to perform this critical assessment and although activities are shown, the physical nature of the oligomer or aggregate has not been characterized.

5. Live cell area seems to contradict some of the early data suggesting inhibition of toxicity. Is this due to the multiple effectors being injected? For example, Bis is highly effective at maintenance of the live cell area while Pol and Zp aren't. Pol and Zp seemed to work better in other assays.

6. The authors test several compounds, salts or derivatives to address the mechanisms mediating inhibition of phospholipase or toxic activity in cells. This approach eliminates compounds or eliminates chemical entities but seems to never address the biochemical mechanisms mediating enzymatic inactivation. How do these compounds interact with the enzyme? There is reasonable speculation (e.g. "both peptide length and charge, as well as the cyclic structure are critical for ExoU inhibition") but no definitive series of experiments or models to drive inhibition to the mechanistic level.

7. Lines 294-306. The discussion of changes in stability during heat denaturation in the presence and absence of compounds and PIP2 suggests that PIP2 is absolutely required for a stable conformation and hence enzymatic activity. Is this true as there is biophysical literature characterizing this enzyme in which no PIP2 is included in their enzyme assays. This is confusing.

8. Testing many clinical isolates is laudable. It may be too simplistic, however, to bin them into ExoS and ExoU expression strains. Other toxic factors and metabolites could be expressed during in vitro and in vivo infection. The inhibitors could be having effects on the eukaryotic cells or tissues, which is not discussed. Since the authors use many tissues and cells and different measurement parameters, it complicates the interpretation of the outcomes. Carefully consider the use of each model and provide some rationale for incorporating those data into the paper.

**********

**Part III – Minor Issues: Editorial and Data Presentation Modifications**

Please use this section for editorial suggestions as well as relatively minor modifications of existing data that would enhance clarity.

Reviewer #1: It appears that there is missing text between lines 88 and 89 in the Introduction.

Lines 86-88 in the Introduction are very similar to lines 75-77. One of these statements should be eliminated.

It appears that the labeling of Figures 2A, 2B, 2C, and 2D is incorrect compared to what is in the text and legend. Figure 2E is not labeled.

Labeling is also incorrect for Figures 1A, 1B, 1C, and 1D is incorrect compared to what is in the text and legend. Figure 1E is not labeled.

In the ex vivo porcine corneal model, it is unclear as to how epithelial ulceration can be assessed when the corneal epithelium is debrided. This must be clarified.

Lines 645 and 676: There is discussion about “blinded” observers. “Blinded” should not be used in ophthalmic research papers. “Masked” is a more appropriate term.

The authors should also discuss the clinical use of polymyxin B, which is a treatment for P. aeruginosa keratitis and whether clinical signs of infection are reduced more with polymyxin B compared with other common treatments of P. aeruginosa keratitis such as topical ciprofloxacin or tobramycin.

Reviewer #2: L64: Reference 4 needs to be updates, in the 2024 WHO priority list P. aeruginosa does not appear, hence the sentence needs correction.

Line 89 Starts with citations so needs to be rewritten.

Line 94 This section would benefit from a more expansive overview of the literature with respect to the inhibition of the T3SS as it would contextualize the findings to come and actually highlight the potency of the combination in comparison to prior approaches. These could include PMID: 38686217; 20176902; 25638499.

Figure 1A: There is a cluster of compounds at around 2000 that all appear to reduce activity. Can the authors expand on what these were, were they part of the same family of compounds? It would be useful in this figure also if the 35 hits that were further evaluated were coloured in red so they activity can be contextualized.

Figure 1D: Why abbreviate Chlorhexidine to Chlora? Mox is not defined in the legend. I would also include in which samples Pa was added, it is not understood at a glance.

L119-120: Sup. Fig. S1, lanes of the gel not labelled.

L126: any rationale for using this ExoU concentration?

Line 144: Could the full screen be published as part of supplemental or at least the other 35 hits?

•L149-150: abbreviations not defined in the text (only in figure legend).

Line 156: I think this section would benefit from a more detailed description of each of the hits. It feels glossed over that some of these are antibiotics, in particular polymyxin. Why for example mention that cyclo has adverse side effects but not highlight the toxicity associated with Polymyxin? Or that Chlorhexidine is a commonly used antiseptic?

Line 169: why not just use a lower MOI rather than introducing another variable in moxifloxacin? How can the authors rule out synergistic effects between Bis, Zp, Pol and mox?

Line 169 Given that some of the hits are known antibiotics or antiseptics, an non-exoU producing strain should be used here (isogenic mutant in PA103 or if necessary another ExoU- strain) to delineate between the antimicrobial and antivirulence effects of each these leads?

Line 193: Bactericidal activity should be measured in Cation Adjusted Muller Hinton Broth as per standard testing guidelines, this is particularly important for Polymyxin. The concentrations discussed here are also quite confusing for example Line 185 suggests Pol was effective at 1um in the infection model but in Line 196 the IC50 was 0.75 um in Lb but 2.1 um in DMEM/F-12, however how this is plotted in figure Supplementary Figure 3 C is not easy to interpret. Given the concentrations between bactericidal and no bactericidal are relatively close, the units on the X axis should be changed to make this easier to interpret, uM instead of Log10 for example. It would also be beneficial if they were plotted us ug/ml as this will align better with traditional susceptibility profiling.

Supplementary Figure 3. Statistics need to be applied throughout.

Line 245: Do the authors have any evidence that the Zp is entering the host cell? Is it possible it is interacting with ExoU in the bacterial cell?

Fig2: panels D and E not labelled.

L262-266: so the bismuth salt would not need to get into the cell? Any idea of possible reasons for this difference with Zn?

Figure 3B what do the boxes signify and why are they number? The figure legend and the corresponding results text needs to be improved here as there are several leaps made without the necessary explanation and interpretation of the data. Lane should also be numbered in the figure as they are consistently referred to in the text. Units of the marker are missing. What oligomerisation states would the molecular weights indicate theoretically?

Fig3C. Why were the pairs not tested?

L347-348: what value of activity would be the control to compare these activities to?

Line 351: Bactericidal activity of the combinations needs to be included as this is important for later experiments.

Figure 4: This results section again needs to improve to enable clarity and easier interpretation of the data. Why is the concentration used in all these assays now 5 um?

L382-383: a bit pedantic, but expression is applied to genes. Proteins are produced.

L385: then would there be one or two ubiquitins in the gel shown in Fig4A?

L396: what is the rationale for this hypothesis? Even though it is demonstrated how the authors get to this idea, it is very hard to follow if it is not better explained at this point.

Line 413 Section starting here needs to be completely rewritten to accurately explain the assays and interpret the findings. Figure 5 needs statistics throughout. The differences look modest at best so it would be important to understand how the authors are determining an effect and what the threshold for protection is. For example in Figure 5D I am struggling to understand how 48% of these are demonstrating protection, particularly as error bars overlap on nearly all of the strains shown. The data in supplemental table 2 is not helpful for this. Pairwise comparisons of each isolate with and without treatment at 4.5 hours is needed.

L424: why was the combination Pol+Zp not tested?

Figure 5: Why is there no difference in Figure 5 C and D for strain PA103? When in Figure 1D there is a clear difference. This is a concern.

Line 472: why was the triple combination not tried here?

L468-469: I would be more inclined to think this is additional mechanism rather then the others.

Line 488 again thresholds need to be set here as to what was considered protection is needed.

L474-477: to complete claim how promising this is, the safety of the triple combination has to be shown as in sup. Fig. S4.

Supplementary Figure 8. Needs to be reformatted for greater clarity

Supplementary figure 10 should say PAO1 not PA01.

Figure 7: No details on how many replicates in legends and it appears only 5 Galleria in total were evaluated, whereas the standard in the field would be 10 Galleria per condition and three independent biological replicates resulting in n=30. If the maximum health index score was 10 as per methods then why is the Y axis going to 11? Also it appears that there is a significant decrease in the health index score with the mice given PBS only. This is a worry as typically you would expect <10% mortality in this group and due to overlapping bars in Panel C it is not possible to see what the final survival was for the PBS only group.

L479-503: This section does not make much sense after presenting all the previous results (ExoU specificity, ExoS+ strains not affected, etc.).

L516: Pa in italics.

Fig7C: orphan symbols in the legend and explanation of this panel missing in the legend. For panel B, left and right in the legend should be top and bottom?

L596, L611: This is odd because looking at the graph is seems that the final mortality is not very close to the uninfected control.

Line 608: How can the authors claim all larvae in the triple treatment group remained healthy at 32 hours, when in Figure 7b Lower panel the HIS is at 3/10?

Line 616: The writing, clarity and interpretation of the data needs to be greatly improved in this section.

Overall, resistance to these approaches should also be dealt with properly in the discussion at least as while the probability of resistance is less, there is still evidence of resistance to T3SS inhibitors (PMID: 24468789).

L690-691: no Fig9 shown

Reviewer #3: Minor issues

1. The authors state that rExoU was made with a 6x-his tag at the N-terminus (lines 117-118) yet in the materials and methods they state the rExoU was fused to a C-terminal 6x His tag (lines 867-686). Please clarify or correct.

2. Line 89 begins with 2 citations?

3. Formatting PLA2G4C (lines 213-226) and likely throughout.

4. Line 244-246. Might the Zp also need a carrier to enter PA103?

5. Figure 2 may be mislabeled? No 2D, Line 270, 276 and no 2E, Line 272.

6. Line 292, weather?

**********

PLOS authors have the option to publish the peer review history of their article (what does this mean?). If published, this will include your full peer review and any attached files.

If you choose “no”, your identity will remain anonymous but your review may still be made public.

**Do you want your identity to be public for this peer review?**  For information about this choice, including consent withdrawal, please see our Privacy Policy.

Reviewer #1: No

Reviewer #2: No

Reviewer #3: No

**Figure resubmission:**

-->While revising your submission, we strongly recommend that you use PLOS’s NAAS tool (https://ngplosjournals.pagemajik.ai/artanalysis) to test your figure files. NAAS can convert your figure files to the TIFF file type and meet basic requirements (such as print size, resolution), or provide you with a report on issues that do not meet our requirements and that NAAS cannot fix.

After uploading your figures to PLOS’s NAAS tool - https://ngplosjournals.pagemajik.ai/artanalysis, NAAS will process the files provided and display the results in the "Uploaded Files" section of the page as the processing is complete. If the uploaded figures meet our requirements (or NAAS is able to fix the files to meet our requirements), the figure will be marked as "fixed" above. If NAAS is unable to fix the files, a red "failed" label will appear above. When NAAS has confirmed that the figure files meet our requirements, please download the file via the download option, and include these NAAS processed figure files when submitting your revised manuscript.

**Reproducibility:**

To enhance the reproducibility of your results, we recommend that authors of applicable studies deposit laboratory protocols in protocols.io, where a protocol can be assigned its own identifier (DOI) such that it can be cited independently in the future. Additionally, PLOS ONE offers an option to publish peer-reviewed clinical study protocols. Read more information on sharing protocols at https://plos.org/protocols?utm_medium=editorial-email&utm_source=authorletters&utm_campaign=protocols

# Author comment: Author response to Decision Letter 1

**DOI:** 10.1371/journal.ppat.1014474.r002

8 May 2026

Attachment Submitted filename: response to reviewers.docx

# Peer review: Decision Letter 1

**DOI:** 10.1371/journal.ppat.1014474.r003

**Authors:** Thomas Guillard, Patricia Bernal

8 Jun 2026

PPATHOGENS-D-25-02908R1

Topical formulation of repurposed FDA-approved compounds inhibits *pseudomonas aeruginosa*  ExoU and improves corneal infection outcomes

PLOS Pathogens

Dear Dr. Foulkes,

Thank you for submitting your manuscript to PLOS Pathogens. Your manuscript has been evaluated by the same three reviewers who assessed your original submission. Whilst the reviewers noted the significant amount of work you have put into this revision, they still have some remaining concerns that must be addressed. The main focus of this revision should be on providing missing methodological details regarding your in vivo models, clarifying specific data presentation points, and addressing Reviewer 1's concerns regarding the statistical analysis of the clinical scores in the mouse keratitis model.

Please submit your revised manuscript by Aug 07 2026 11:59PM. If you will need more time than this to complete your revisions, please reply to this message or contact the journal office at plospathogens@plos.org. When you're ready to submit your revision, log on to https://www.editorialmanager.com/ppathogens/ and select the 'Submissions Needing Revision' folder to locate your manuscript file.

Please include the following items when submitting your revised manuscript:

* A letter that responds to each point raised by the editor and reviewer(s). You should upload this letter as a separate file labeled 'Response to Reviewers'. This file does not need to include responses to any formatting updates and technical items listed in the 'Journal Requirements' section below.

* A marked-up copy of your manuscript that highlights changes made to the original version. You should upload this as a separate file labeled 'Revised Manuscript with Track Changes'.

* An unmarked version of your revised paper without tracked changes. You should upload this as a separate file labeled 'Manuscript'.

If you would like to make changes to your financial disclosure, competing interests statement, or data availability statement, please make these updates within the submission form at the time of resubmission. Guidelines for resubmitting your figure files are available below the reviewer comments at the end of this letter.

As the corresponding author, your ORCID iD is verified in the submission system and will appear in the published article. PLOS supports the use of ORCID, and we encourage all coauthors to register for an ORCID iD and use it as well. Please encourage your coauthors to verify their ORCID iD within the submission system before final acceptance, as unverified ORCID iDs will not appear in the published article. Only the individual author can complete the verification step; PLOS staff cannot verify ORCID iDs on behalf of authors.

We look forward to receiving your revised manuscript.

Kind regards,

Patricia Bernal

Academic Editor

PLOS Pathogens

Thomas Guillard

Section Editor

PLOS Pathogens

Sumita Bhaduri-McIntosh

Editor-in-Chief

PLOS Pathogens

orcid.org/0000-0003-2946-9497

Michael Malim

Editor-in-Chief

PLOS Pathogens

orcid.org/0000-0002-7699-2064

**Additional Editor Comments (if provided):**

**Journal Requirements:**

If the reviewer comments include a recommendation to cite specific previously published works, please review and evaluate these publications to determine whether they are relevant and should be cited. There is no requirement to cite these works unless the editor has indicated otherwise.

1) Please amend your detailed Financial Disclosure statement. This is published with the article. It must therefore be completed in full sentences and contain the exact wording you wish to be published.

i) Please clarify all sources of financial support for your study. List the grants, grant numbers, and organizations that funded your study, including funding received from your institution. Please note that suppliers of material support, including research materials, should be recognized in the Acknowledgements section rather than in the Financial Disclosure

ii) State the initials, alongside each funding source, of each author to receive each grant. For example: "This work was supported by the National Institutes of Health (####### to AM; ###### to CJ) and the National Science Foundation (###### to AM).".

If you did not receive any funding for this study, please simply state: u201cThe authors received no specific funding for this work.u201d

iii)Your current Financial Disclosure states, “This study was supported by the Fight for Sight (grant numbers 5175 and 5176 awarded to S.K.). D.F. received salary support from these grants. Additional support was provided by the Biotechnology and Biological Sciences Research Council (BBSRC grant BB/X002780/1 supporting J.H.). Imaging experiments were performed using equipment within the University of Liverpool Centre for Cell Imaging, including instrumentation funded by Medical Research Council grant MR/X013502/1 and MR/K015931/1. The funders had no role in study design, data collection and analysis, decision to publish, or preparation of the manuscript.”. However, your funding information on the submission form indicates mismatched funder name and includes fields for grant recipient information. Please indicate by return email the full and correct funding information for your study and confirm the order in which funding contributions should appear. Please be sure to indicate whether the funders played any role in the study design, data collection and analysis, decision to publish, or preparation of the manuscript.

2) PLOS now requires that authors provide the original uncropped and unadjusted images underlying all blot or gel results reported in a submission’s figures or Supporting Information files. This policy and the journal’s other requirements for blot/gel reporting and figure preparation are described in detail at https://journals.plos.org/plosone/s/figures#loc-blot-and-gel-reporting-requirements and https://journals.plos.org/plosone/s/figures#loc-preparing-figures-from-image-files.

When you submit your revised manuscript, please ensure that your figures adhere fully to these guidelines and provide the original underlying images for all blot or gel data reported in your submission. See the following link for instructions on providing the original image data: https://journals.plos.org/plosone/s/figures#loc-original-images-for-blots-and-gels.

In your cover letter, please note whether your blot/gel image data are in Supporting Information or posted at a public data repository, provide the repository URL if relevant, and provide specific details as to which raw blot/gel images, if any, are not available. Email us at customercare@plos.org if you have any questions.

**Reviewers' Comments:**

Reviewer's Responses to Questions

**Part I - Summary**

Please use this section to discuss strengths/weaknesses of study, novelty/significance, general execution and scholarship.

Reviewer #1: Details regarding the mouse keratitis model are lacking as well as an inappropriate statistical analysis of the clinical data.

Reviewer #2: All my concerns have been dealt with and additional data has been included to support claims.

Reviewer #3: The authors have revised the manuscript to where a cursory reading allows a casual reader to pick up the main points. The testing is clearly comprehensive and the conclusions very similar: multiple drug combinations work better, the drugs inhibit ExoU phospholipase activity but don't affect bacterial growth or T3SS expression and have varying efficacy depending on the strain and the specific test. New data has been added making it a somewhat different paper than initially submitted. It is still quite a long read and has minor issues that should be corrected. While the authors are addressing an important human health problem, the significance seems limited to the use of drug cocktails rather than pursuing one of the mechanisms of action and improving drug efficacy.

**********

**Part II – Major Issues: Key Experiments Required for Acceptance**

Please use this section to detail the key new experiments or modifications of existing experiments that should be absolutely required to validate study conclusions.

Generally, there should be no more than 3 such required experiments or major modifications for a "Major Revision" recommendation. If more than 3 experiments are necessary to validate the study conclusions, then you are encouraged to recommend "Reject".

Reviewer #1: This reviewer has concerns regarding the mouse keratitis model studies:

1. The number of animals per group is not stated.

2. Were both eyes or one eye infected and treated?

3. For the clinical scoring of the eyes, mean ± SD scores should not be presented for graded data. Median scores with interquartile ranges should be presented.

4. The clinical data for is not continuous. There are only 16 scores plus zero that can be used. Therefore a nonparametric analysis should be used.

5. It is unclear what the values represent under “Condition” in Tables 2 and 3. These values must be defined.

6. Figure 8A. What is the clinical score? Mean? Median? What do the error bars represent.

7. How were the ulcer sizes determined? Was fluorescein staining used?

Reviewer #2: none

Reviewer #3: NA

**********

**Part III – Minor Issues: Editorial and Data Presentation Modifications**

Please use this section for editorial suggestions as well as relatively minor modifications of existing data that would enhance clarity.

Reviewer #1: 1. For the Galleria mellonella studies, were the larvae injected with the ExoU inhibitors without infection to determine any potential toxicity?

Reviewer #2: (No Response)

Reviewer #3: 1.Line 103-104. Reference?

2. A surprising number of compounds inhibit ExoU activity over 100%. For example, ibruprofen, 105.3%. Is this actually % ExoU activity rather than % inhibition?

3. Line 339. The authors need to check the changes in thermal stability calculations. Either that or something is mislabeled (Supplementary Fig 7).

4. Figure 3A. ∆Tm/˚C? How does panel A differ from the information shown in S Fig 7? ∆Tm

for Pol (relative to DMSO = -1.8˚C) -1.8/40.86 = -0.044? What does the Y axis mean?

5. Supplementary Figure 10. Quantitative RT-PCR analysis suggest that the T3SS is transcribing at a basal level in LB medium. How does this relate to T3SS being expressed in cells and under tissue culture conditions? What is Vald?

**********

PLOS authors have the option to publish the peer review history of their article (what does this mean?). If published, this will include your full peer review and any attached files.

If you choose “no”, your identity will remain anonymous but your review may still be made public.

**Do you want your identity to be public for this peer review?**  For information about this choice, including consent withdrawal, please see our Privacy Policy.

Reviewer #1: No

Reviewer #2: No

Reviewer #3: No

[NOTE: If reviewer comments were submitted as an attachment file, they will be attached to this email and accessible via the submission site. Please log into your account, locate the manuscript record, and check for the action link "View Attachments". If this link does not appear, there are no attachment files.]

**Figure resubmission:**

-->While revising your submission, we strongly recommend that you use PLOS’s NAAS tool (https://ngplosjournals.pagemajik.ai/artanalysis) to test your figure files. NAAS can convert your figure files to the TIFF file type and meet basic requirements (such as print size, resolution), or provide you with a report on issues that do not meet our requirements and that NAAS cannot fix.

After uploading your figures to PLOS’s NAAS tool - https://ngplosjournals.pagemajik.ai/artanalysis, NAAS will process the files provided and display the results in the "Uploaded Files" section of the page as the processing is complete. If the uploaded figures meet our requirements (or NAAS is able to fix the files to meet our requirements), the figure will be marked as "fixed" above. If NAAS is unable to fix the files, a red "failed" label will appear above. When NAAS has confirmed that the figure files meet our requirements, please download the file via the download option, and include these NAAS processed figure files when submitting your revised manuscript.

**Reproducibility:**

To enhance the reproducibility of your results, we recommend that authors of applicable studies deposit laboratory protocols in protocols.io, where a protocol can be assigned its own identifier (DOI) such that it can be cited independently in the future. Additionally, PLOS ONE offers an option to publish peer-reviewed clinical study protocols. Read more information on sharing protocols at https://plos.org/protocols?utm_medium=editorial-email&utm_source=authorletters&utm_campaign=protocols

# Author comment: Author response to Decision Letter 2

**DOI:** 10.1371/journal.ppat.1014474.r004

24 Jun 2026

Attachment Submitted filename: Response_to_reviewers_auresp_2.docx

# Editor report: Decision Letter 2

**DOI:** 10.1371/journal.ppat.1014474.r005

**Authors:** Thomas Guillard, Patricia Bernal

16 Jul 2026

Dear Dr Foulkes,

We are pleased to inform you that your manuscript 'Topical formulation of repurposed FDA-approved compounds inhibits *pseudomonas aeruginosa*  ExoU and improves corneal infection outcomes' has been provisionally accepted for publication in PLOS Pathogens.

Before your manuscript can be formally accepted you will need to complete some formatting changes, which you will receive in a follow up email. A member of our team will be in touch with a set of requests.

Please note that your manuscript will not be scheduled for publication until you have made the required changes, so a swift response is appreciated.

IMPORTANT: The editorial review process is now complete. PLOS will only permit corrections to spelling, formatting or significant scientific errors from this point onwards. Requests for major changes, or any which affect the scientific understanding of your work, will cause delays to the publication date of your manuscript.

Should you, your institution's press office or the journal office choose to press release your paper, you will automatically be opted out of early publication. We ask that you notify us now if you or your institution is planning to press release the article. All press must be co-ordinated with PLOS.

Thank you again for supporting Open Access publishing; we are looking forward to publishing your work in PLOS Pathogens.

Best regards,

Thomas Guillard, PharmD, PhD

Section Editor

PLOS Pathogens

Thomas Guillard

Section Editor

PLOS Pathogens

Sumita Bhaduri-McIntosh

Editor-in-Chief

PLOS Pathogens

orcid.org/0000-0003-2946-9497

Michael Malim

Editor-in-Chief

PLOS Pathogens

orcid.org/0000-0002-7699-2064

***********************************************************

Reviewer Comments (if any, and for reference):

# Editor report: Acceptance letter

**DOI:** 10.1371/journal.ppat.1014474.r006

**Authors:** Thomas Guillard, Patricia Bernal

Dear Dr Foulkes,

We are delighted to inform you that your manuscript, "Topical formulation of repurposed FDA-approved compounds inhibits *Pseudomonas aeruginosa*  ExoU and improves corneal infection outcomes," has been formally accepted for publication in PLOS Pathogens.

We have now passed your article onto the PLOS Production Department who will complete the rest of the pre-publication process. All authors will receive a confirmation email upon publication.

The corresponding author will soon be receiving a typeset proof for review, to ensure errors have not been introduced during production. Please review the PDF proof of your manuscript carefully, as this is the last chance to correct any scientific or type-setting errors. Please note that major changes, or those which affect the scientific understanding of the work, will likely cause delays to the publication date of your manuscript. Note: Proofs for Front Matter articles (Pearls, Reviews, Opinions, etc...) are generated on a different schedule and may not be made available as quickly.

Soon after your final files are uploaded, the early version of your manuscript, if you opted to have an early version of your article, will be published online. The date of the early version will be your article's publication date. The final article will be published to the same URL, and all versions of the paper will be accessible to readers.

For Research Articles, you will receive an invoice from PLOS for your publication fee after your manuscript has reached the completed accept phase. If you receive an email requesting payment before acceptance or for any other service, this may be a phishing scheme. Learn how to identify phishing emails and protect your accounts at https://explore.plos.org/phishing.

Thank you again for supporting open-access publishing; we are looking forward to publishing your work in PLOS Pathogens.

Best regards,

Sumita Bhaduri-McIntosh

Editor-in-Chief

PLOS Pathogens

orcid.org/0000-0003-2946-9497

Michael Malim

Editor-in-Chief

PLOS Pathogens

orcid.org/0000-0002-7699-2064
